# Supplementary figures and images for: Was Hupehsuchus a baleen whale-style filter feeder in the Early Triassic? A re-examination of the evidence
Source: PeerJ. 2025 Jul 4;13:e19666. doi: 10.7717/peerj.19666 (PMC12232927; doi:10.7717/peerj.19666)

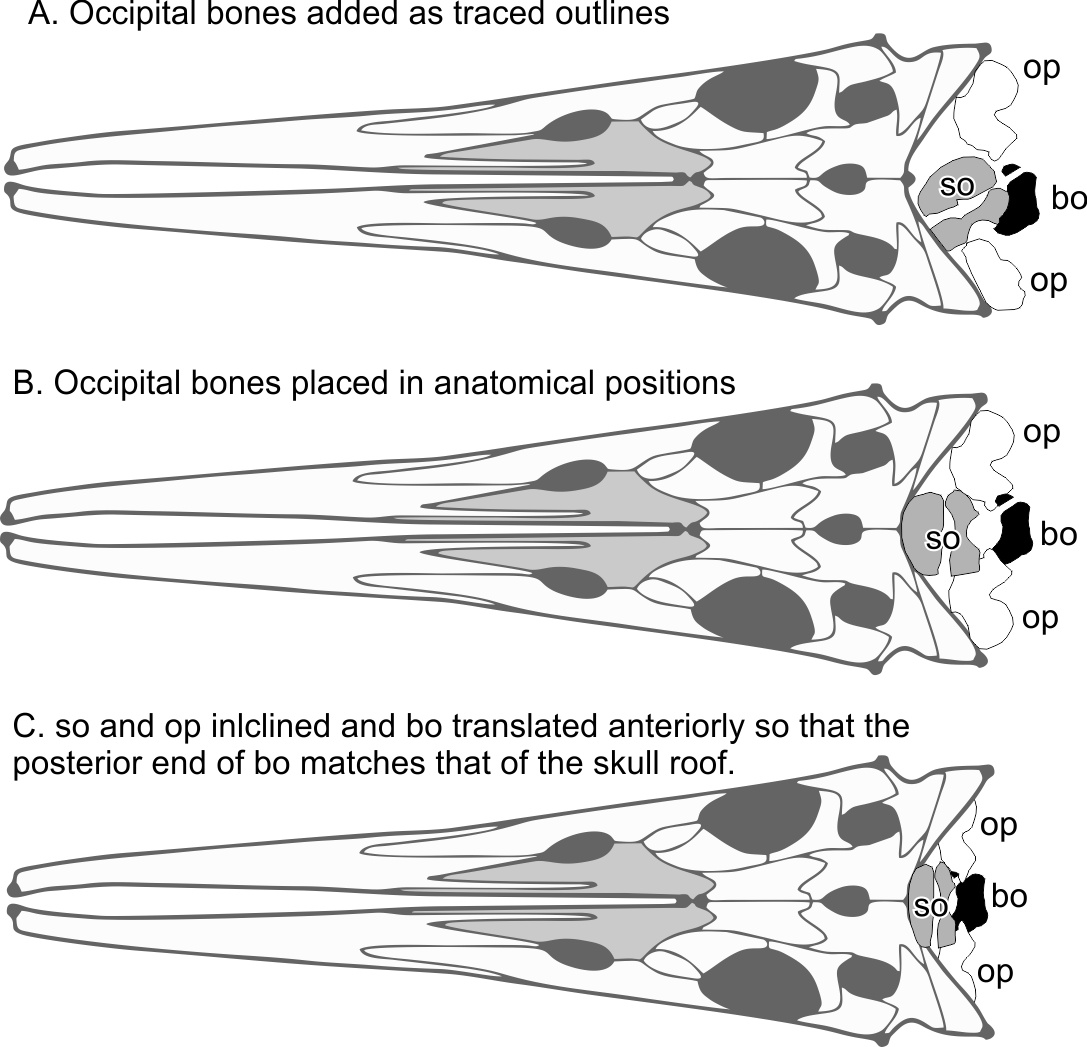

Supplement: Supplemental Information 1 — A, occipital bones were outlined based on fig. 3 of Fang et al. (2023) and some identifications were revised. B, the bones were rearranged in a presumed articulation arrangement. C, considering the vertical spread of the bone in life, the bones in the second step were linearly compressed antero-posteriorly. Skull roof is modified from an image in fig. 2 of Fang et al. (2023). [file peerj-13-19666-s001.jpg]

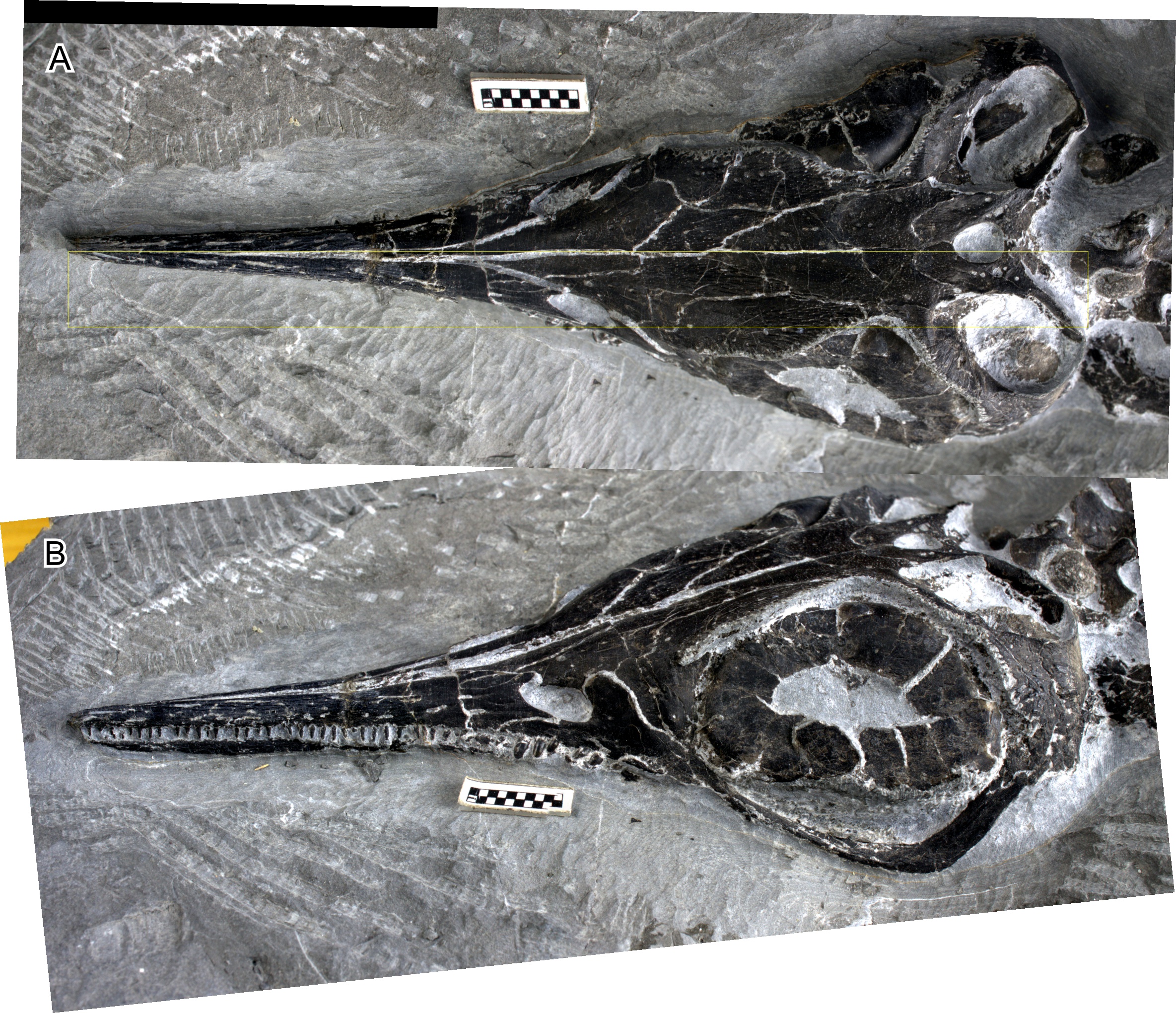

Supplement: Supplemental Information 2 — A, Dorsal view. B, Left lateral view. Scale is 1 cm. [file peerj-13-19666-s002.jpg]

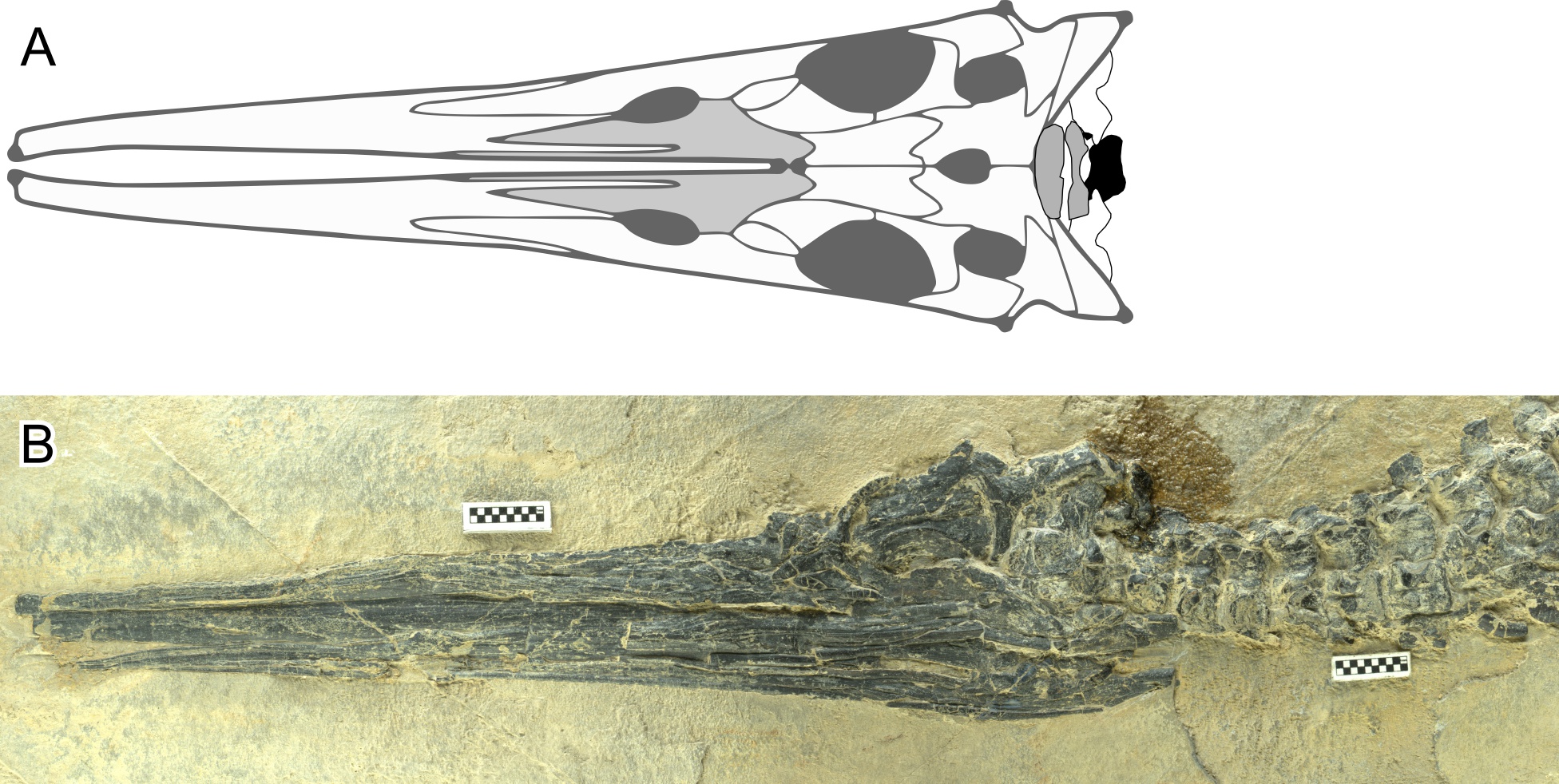

Supplement: Supplemental Information 3 — A, Dorsal view taken from fig. 2 of Fang et al. (2023). B, Left lateral view, based on WGSC V26005. Scale is 1 cm. [file peerj-13-19666-s003.jpg]
