# Supplementary material for: Was Hupehsuchus a baleen whale-style filter feeder in the Early Triassic? A re-examination of the evidence
Source: PeerJ. 2025 Jul 4;13:e19666. doi: 10.7717/peerj.19666 (PMC12232927; doi:10.7717/peerj.19666)
Supplement: Supplemental Information 4 [file peerj-13-19666-s004.pdf]

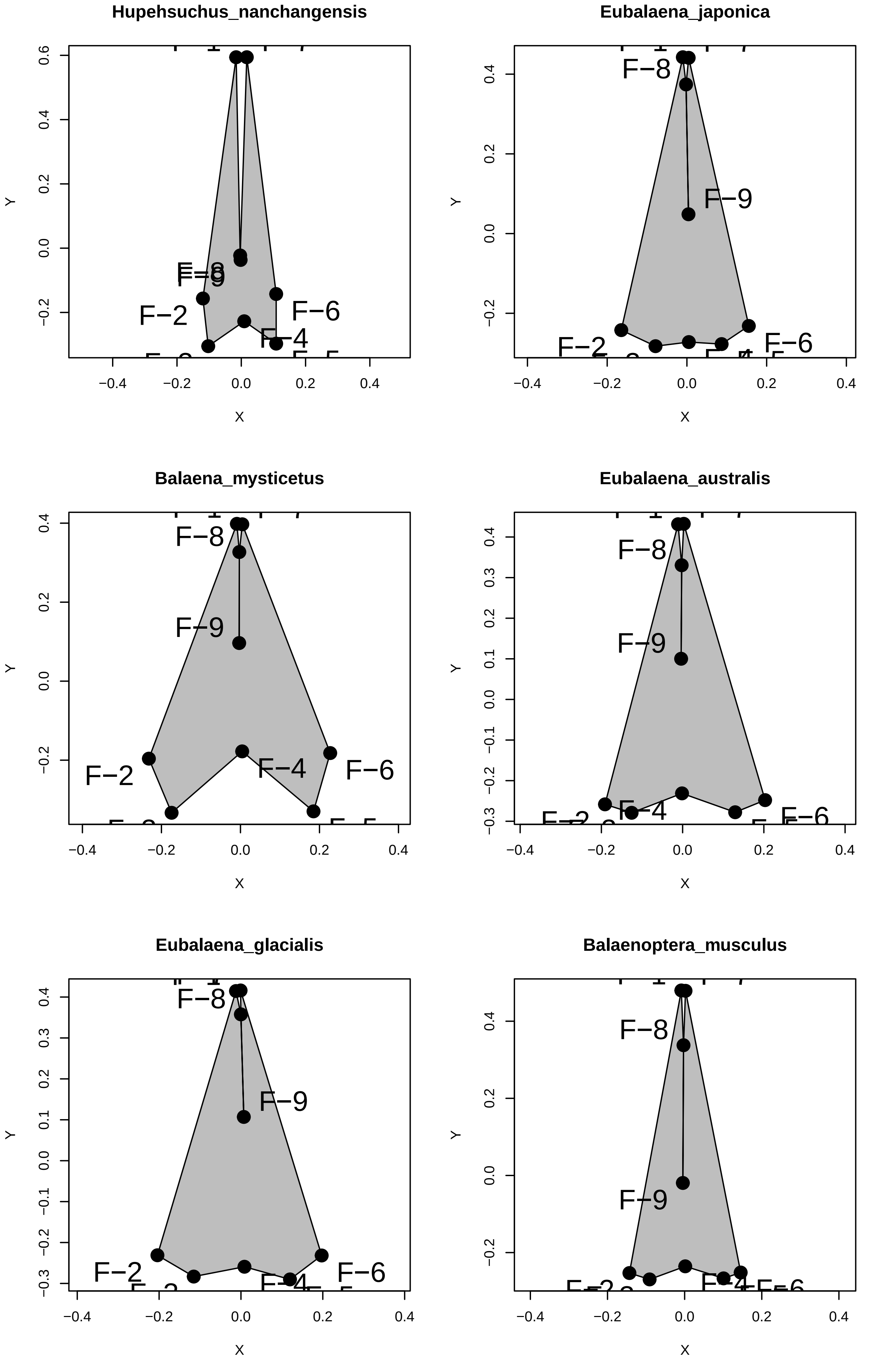

Hupehsuchus_nanchangensis
Eubalaena_japonica
Balaena_mysticetus
Eubalaena_australis
Eubalaena_glacialis
Balaenoptera_musculus
X
Y
F−2
F−4
F−6
F−8
F−9

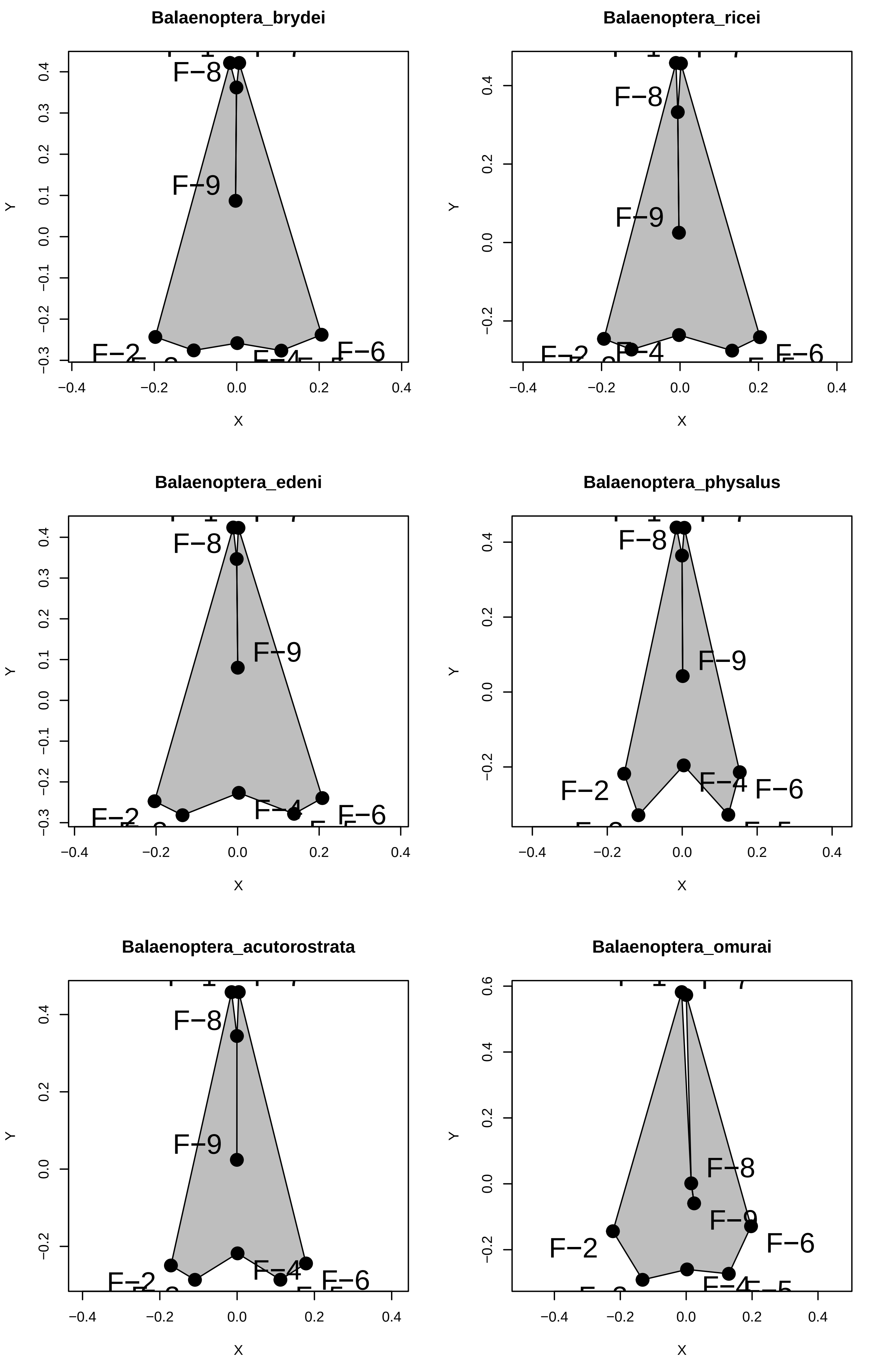
Balaenoptera_brydei
F−8
F−9
F−2
F−6
Balaenoptera_ricei
F−8
F−9
F−4
F−6
Balaenoptera_edeni
F−8
F−9
F−2
F−4
F−6
Balaenoptera_physalus
F−8
F−9
F−2
F−4
F−6
Balaenoptera_acutorostrata
F−8
F−9
F−2
F−4
F−6
Balaenoptera_omurai
F−8
F−9
F−2
F−6
X
Y

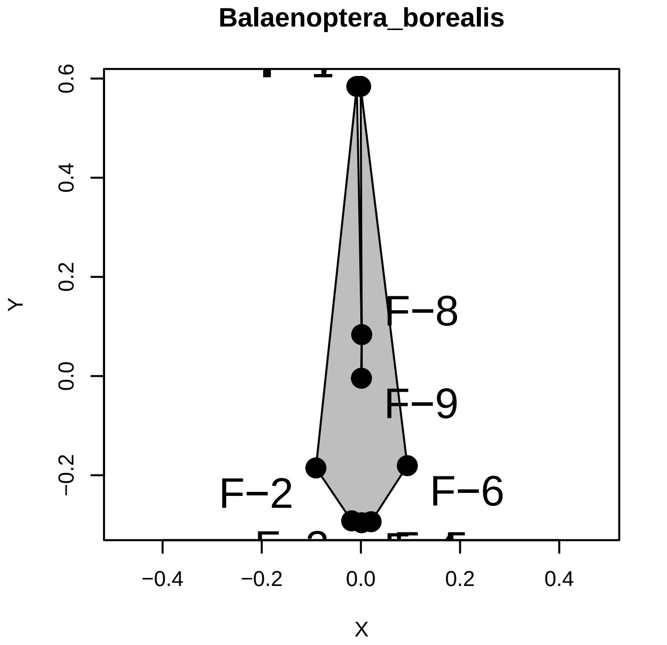
Balaenoptera_borealis
F−8
F−9
F−2
F−6
Y
X
0.6
0.4
0.2
0.0
−0.2
−0.4
−0.2
0.0
0.2
0.4

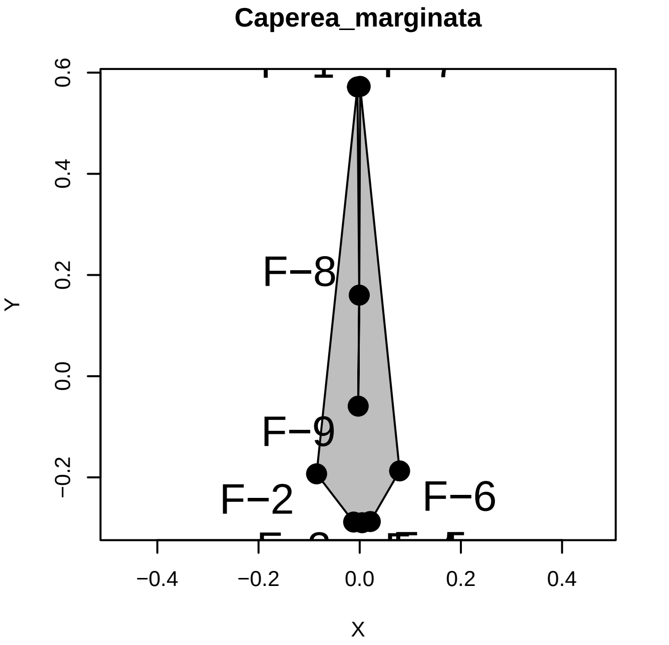
Caperea_marginata
F−8
F−9
F−2
F−6
Y
X
0.6
0.4
0.2
0.0
−0.2
−0.4
−0.2
0.0
0.2
0.4

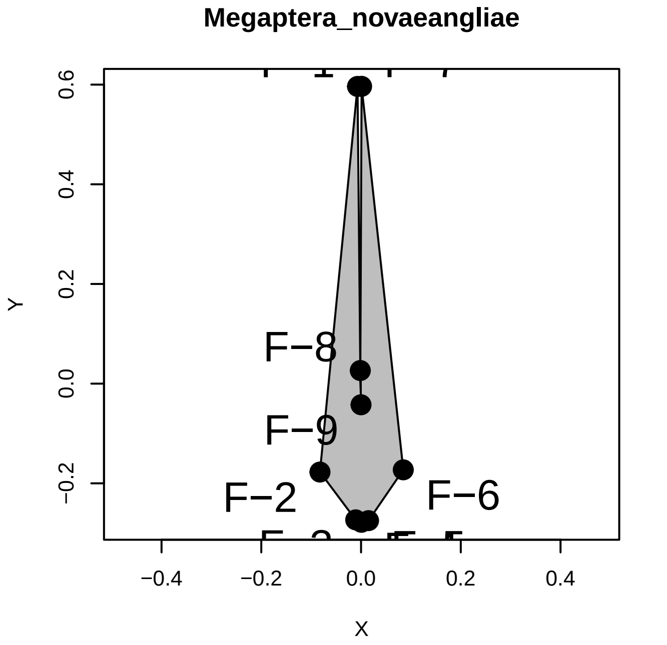
Megaptera_novaeangliae
F−8
F−9
F−2
F−6
Y
X
0.6
0.4
0.2
0.0
−0.2
−0.4
−0.2
0.0
0.2
0.4

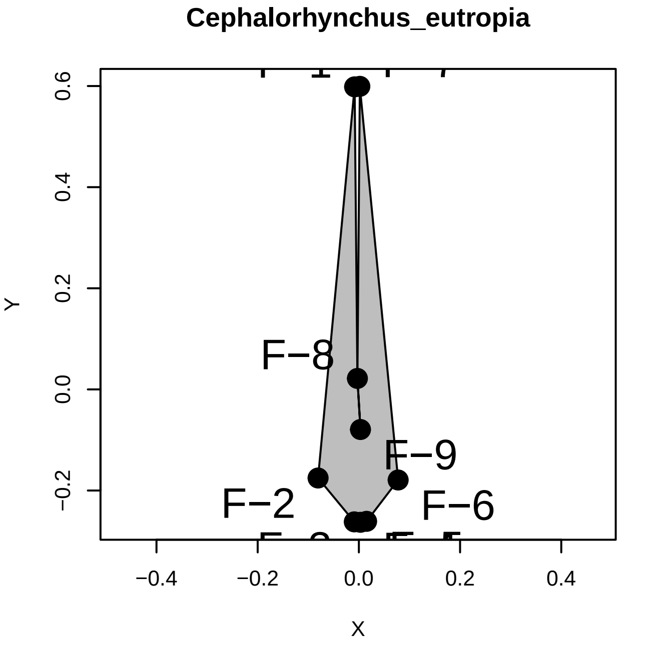
Cephalorhynchus_eutropia
F−8
F−9
F−2
F−6
Y
X
0.6
0.4
0.2
0.0
−0.2
−0.4
−0.2
0.0
0.2
0.4

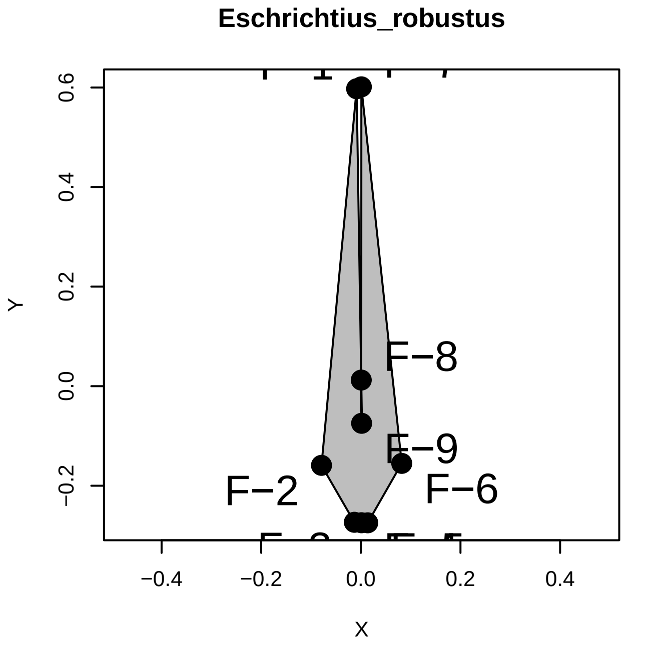
Eschrichtius_robustus
F−8
F−9
F−2
F−6
Y
X
0.6
0.4
0.2
0.0
−0.2
−0.4
−0.2
0.0
0.2
0.4

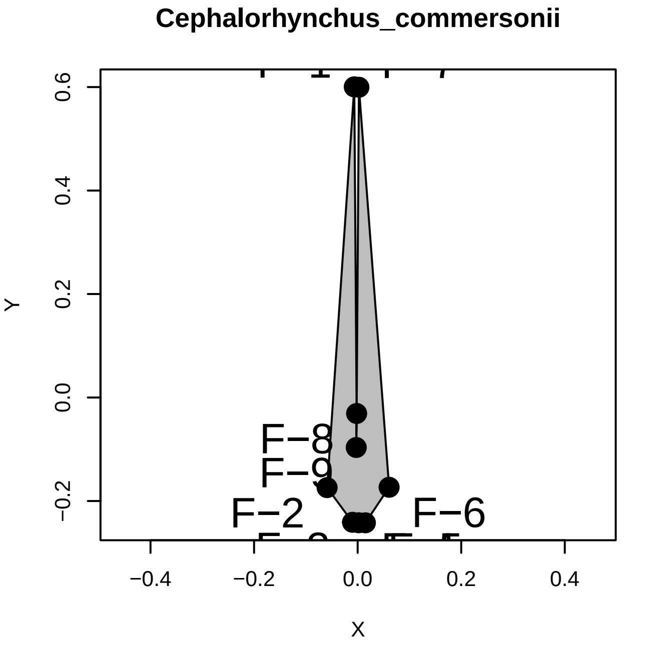
Cephalorhynchus_commersonii
F−8
F−9
F−2
F−6
Y
X
0.6
0.4
0.2
0.0
−0.2
−0.4
−0.2
0.0
0.2
0.4

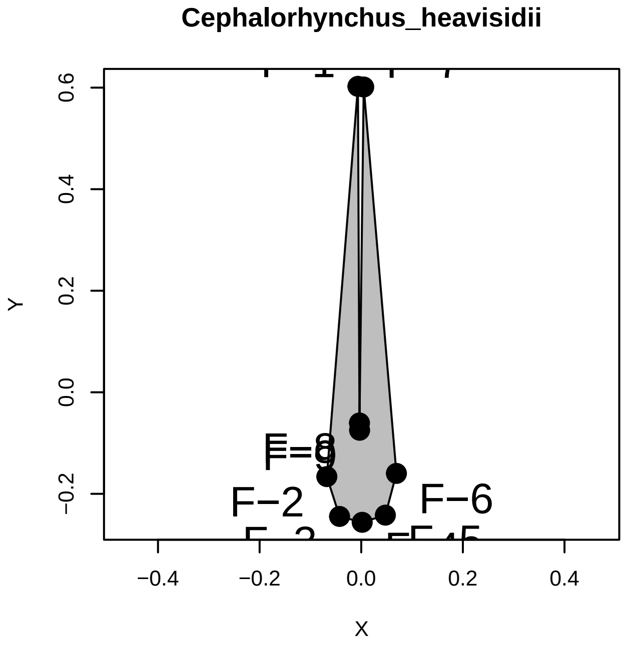
Cephalorhynchus_heavisidii
F−2
F−6
X
Y

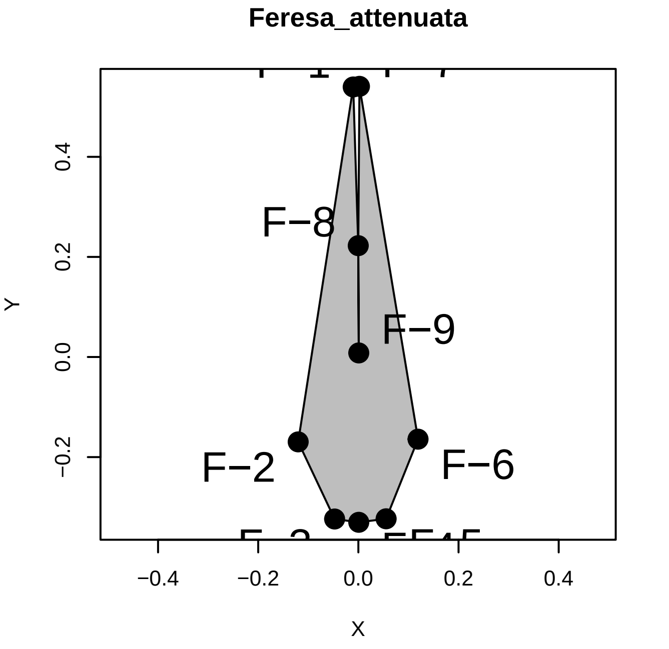
Feresa_attenuata
F−8
F−9
F−2
F−6
X
Y

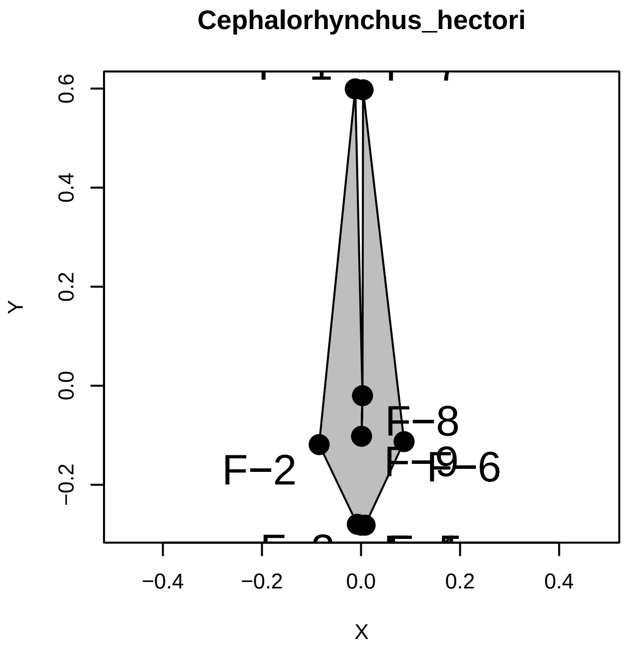
Cephalorhynchus_hectori
F−8
F−2
X
Y

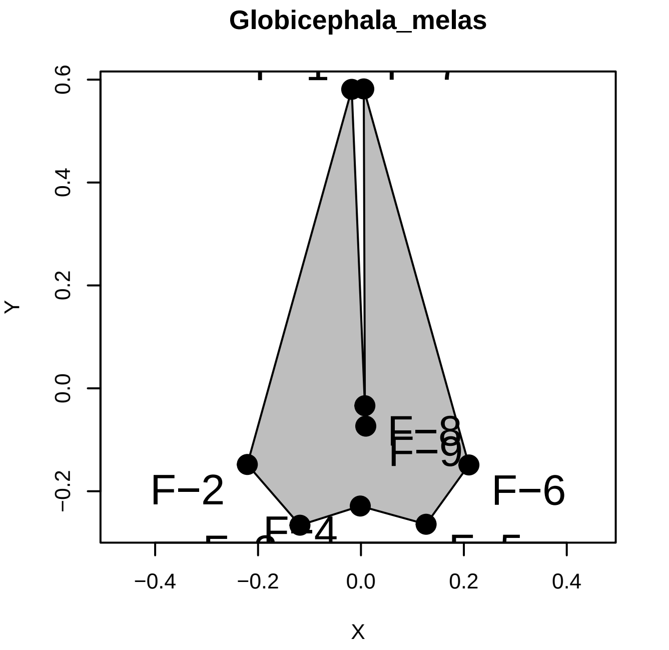
Globicephala_melas
F−2
F−6
X
Y

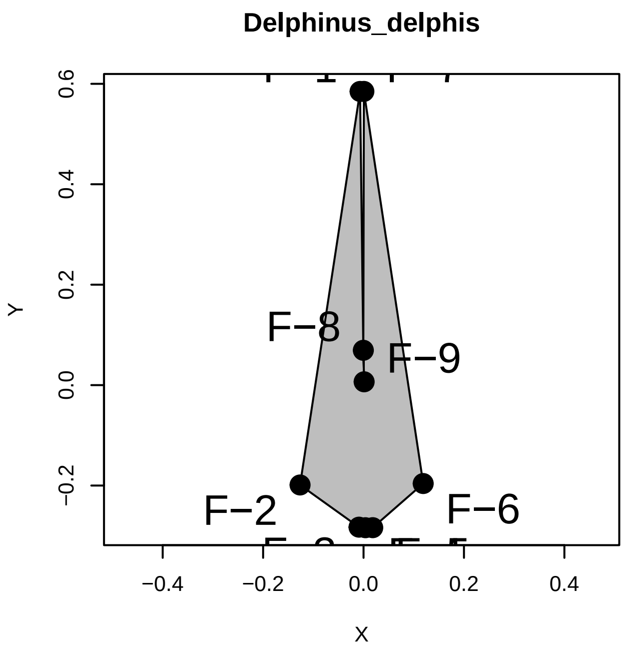
Delphinus_delphis
F−8
F−9
F−2
F−6
X
Y

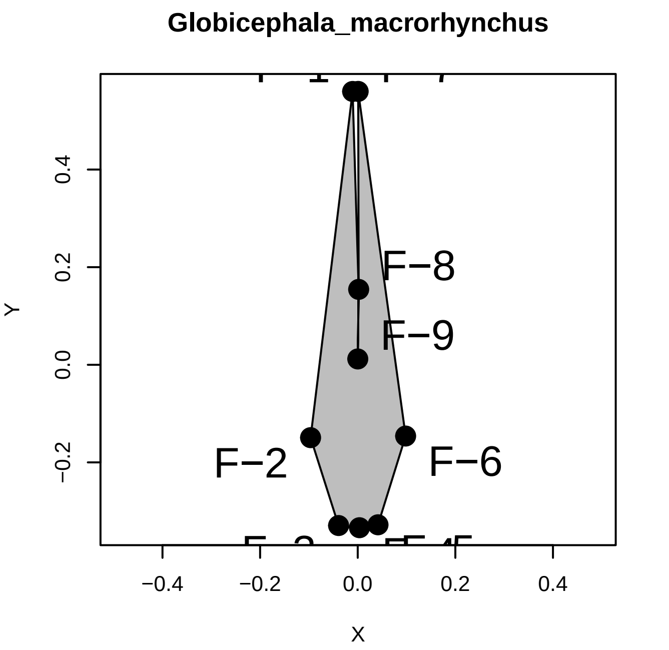
Globicephala_macrorhynchus
F−8
F−9
F−2
F−6
X
Y

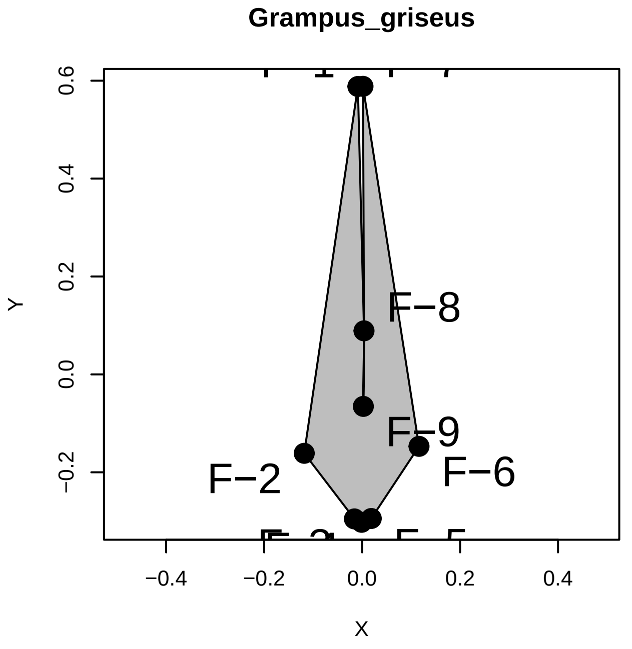
Grampus_griseus
F−8
F−9
F−2
F−6
X
Y

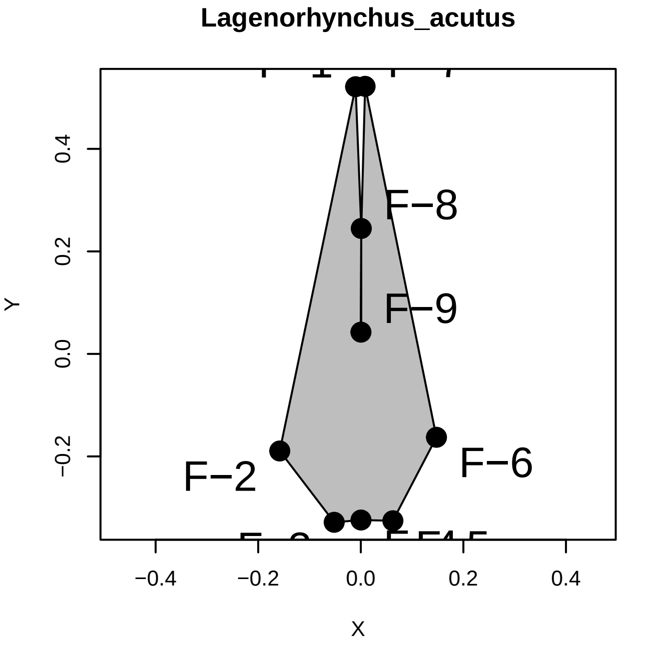
Lagenorhynchus_acutus
F−8
F−9
F−2
F−6
X
Y

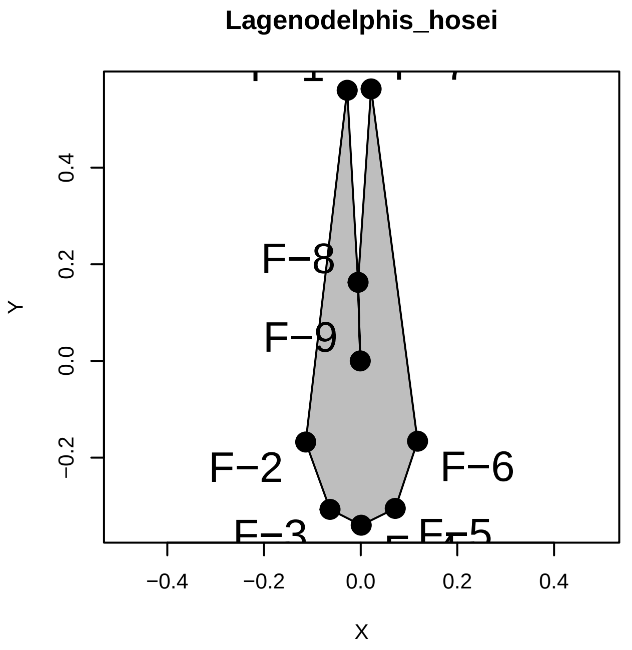
Lagenodelphis_hosei
F−8
F−9
F−2
F−6
X
Y

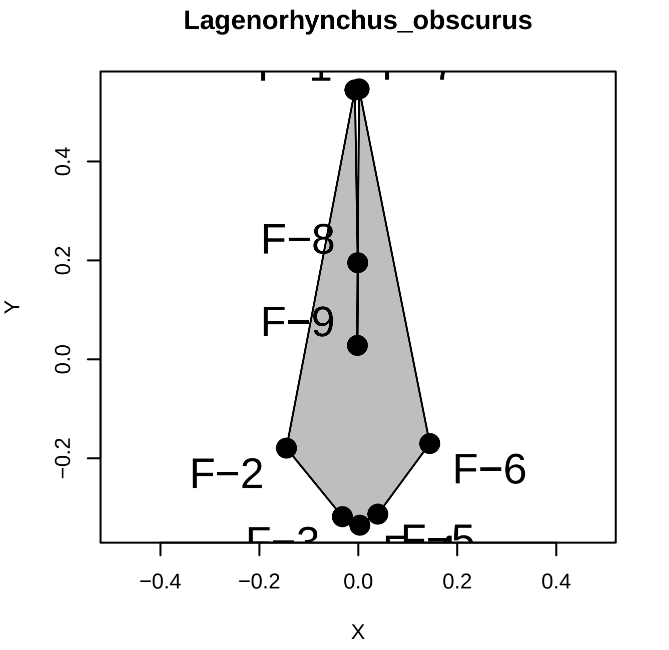
Lagenorhynchus_obscurus
F−8
F−9
F−2
F−6
X
Y

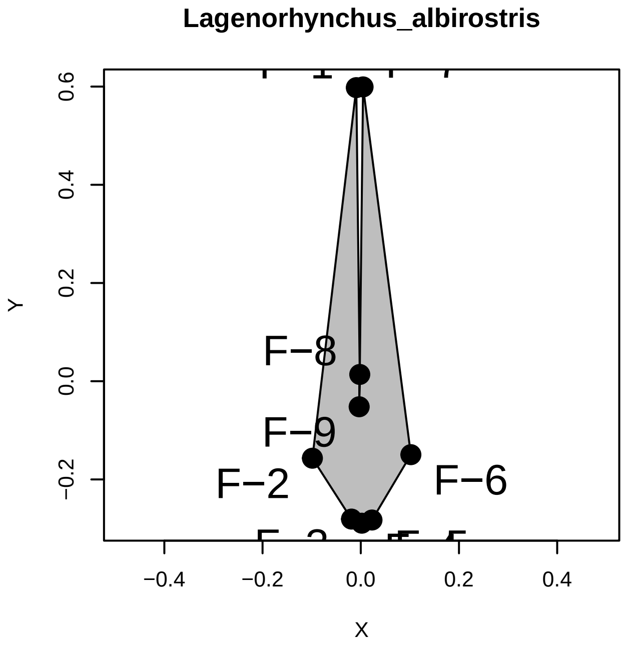
Lagenorhynchus_albirostris
F−8
F−9
F−2
F−6
X
Y

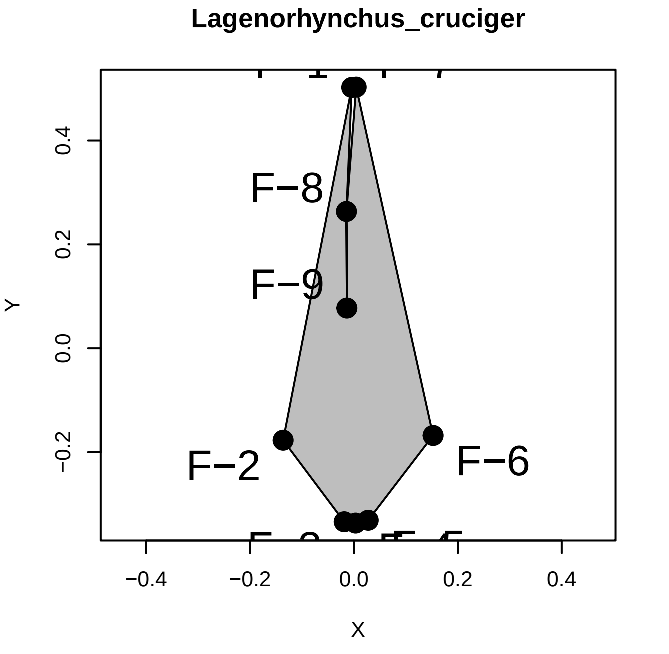
Lagenorhynchus_cruciger
F−8
F−9
F−2
F−6
X
Y

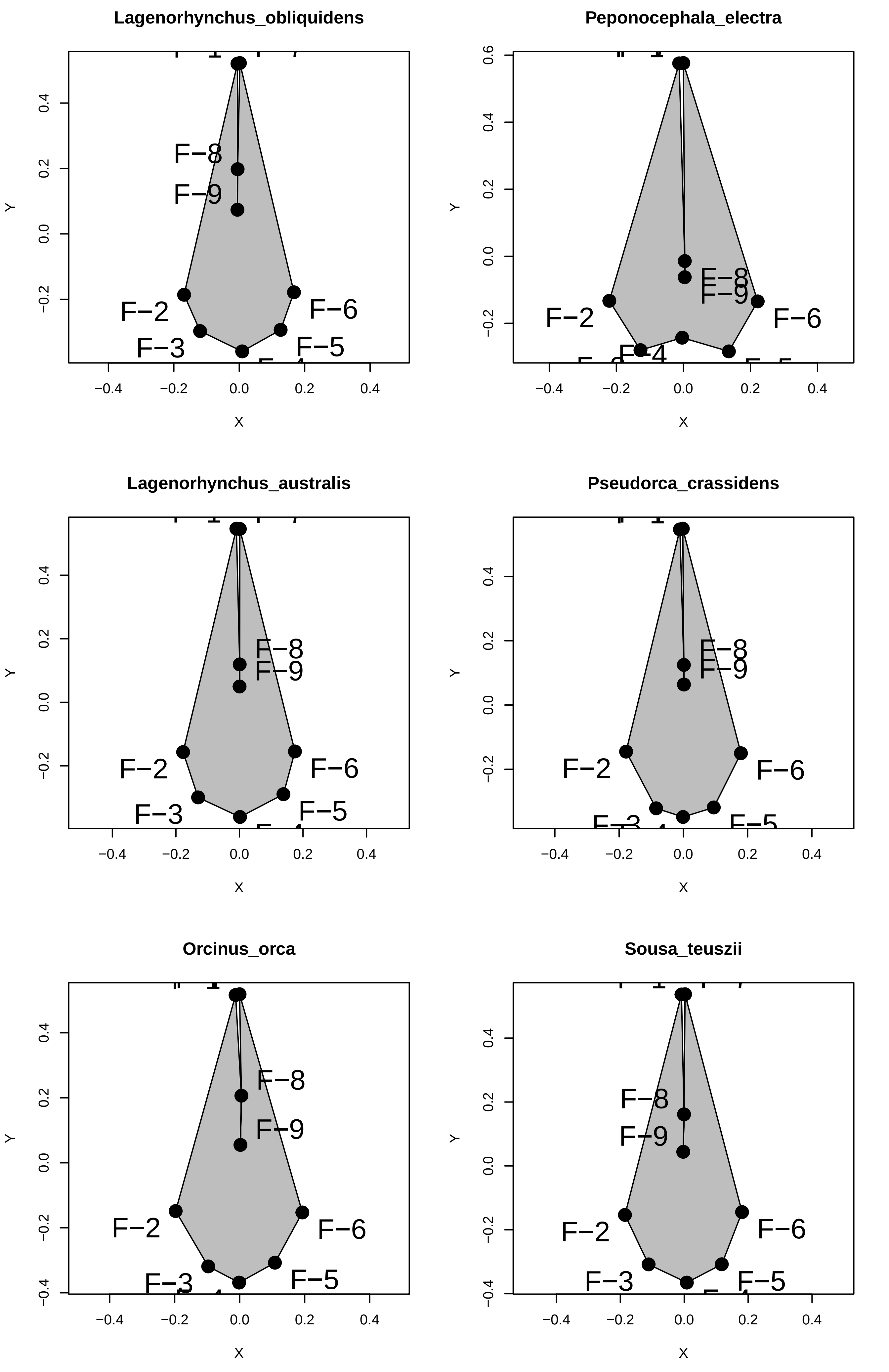
Lagenorhynchus_obliquidens
F−8
F−9
F−2
F−6
F−3
F−5
Peponocephala_electra
F−8
F−9
F−2
F−6
F−4
Lagenorhynchus_australis
F−8
F−9
F−2
F−6
F−3
F−5
Pseudorca_crassidens
F−8
F−9
F−2
F−6
F−5
Orcinus_orca
F−8
F−9
F−2
F−6
F−3
F−5
Sousa_teuszii
F−8
F−9
F−2
F−6
F−3
F−5
X
Y

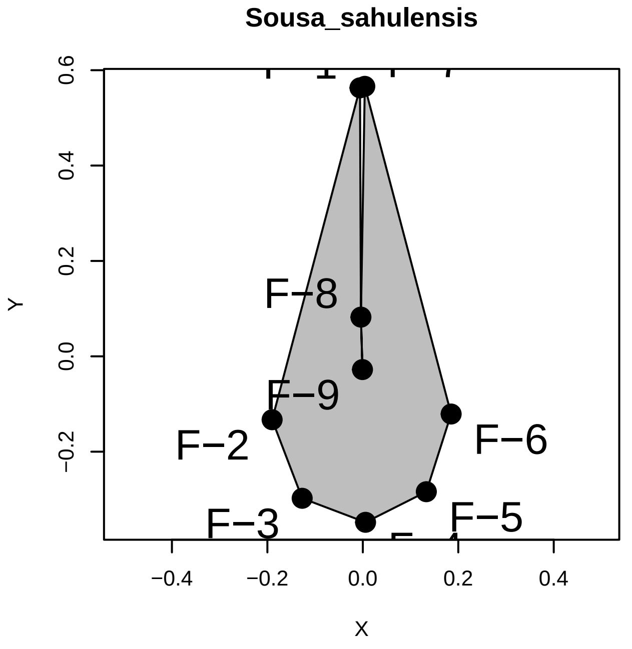
Sousa_sahulensis
F−8
F−9
F−2
F−6
F−3
F−5
0.6
0.4
0.2
0.0
−0.2
−0.4
−0.2
0.0
0.2
0.4
X
Y

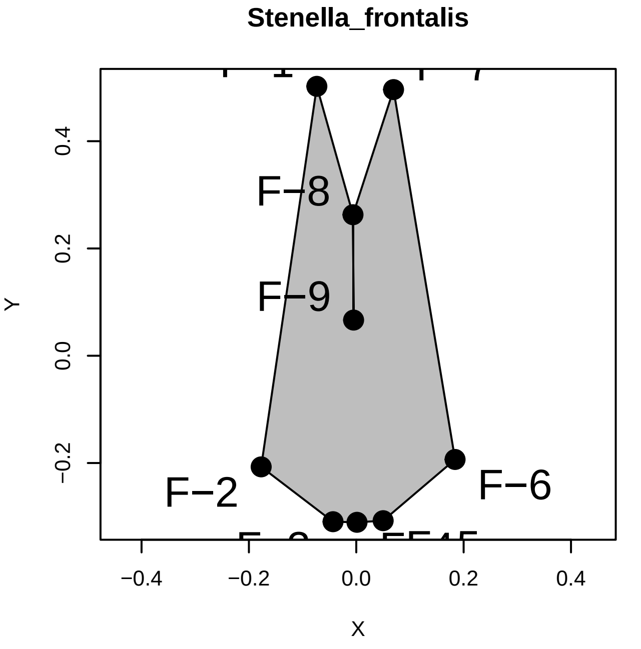
Stenella_frontalis
F−8
F−9
F−2
F−6
0.4
0.2
0.0
−0.2
−0.4
−0.2
0.0
0.2
0.4
X
Y

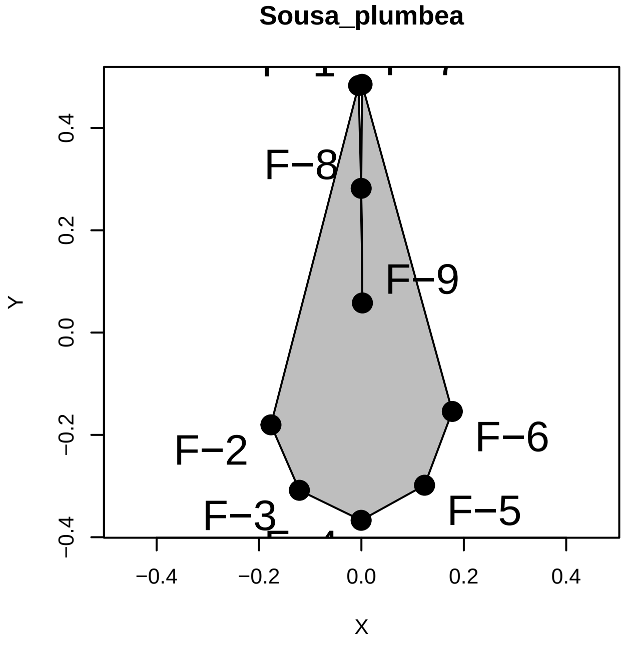
Sousa_plumbea
F−8
F−9
F−2
F−6
F−3
F−5
0.4
0.2
0.0
−0.2
−0.4
−0.4
−0.2
0.0
0.2
0.4
X
Y

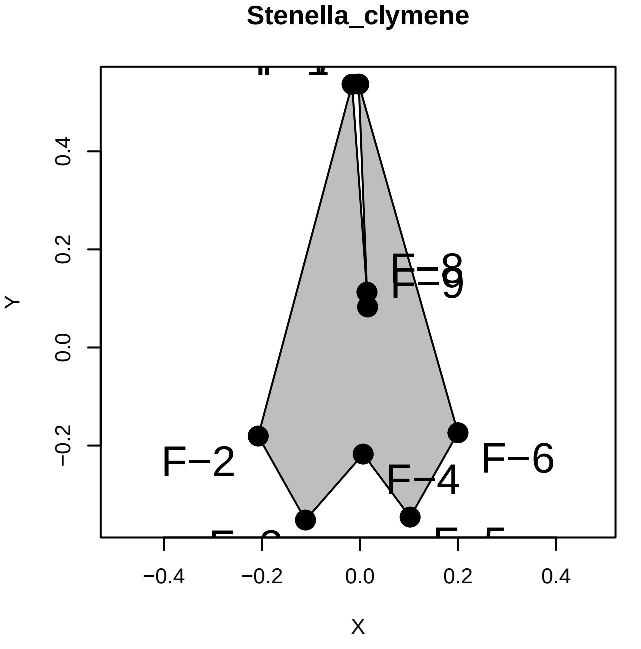
Stenella_clymene
F−8
F−9
F−2
F−6
F−4
0.4
0.2
0.0
−0.2
−0.4
−0.2
0.0
0.2
0.4
X
Y

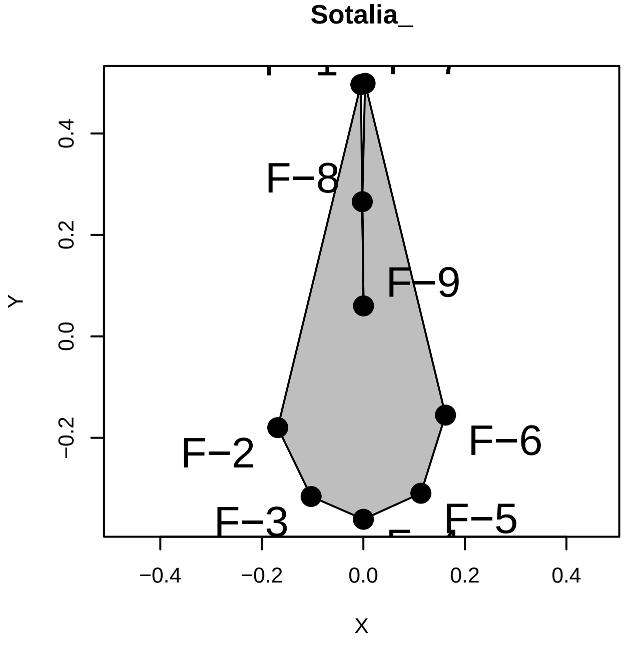
Sotalia_
F−8
F−9
F−2
F−6
F−3
F−5
0.4
0.2
0.0
−0.2
−0.4
−0.2
0.0
0.2
0.4
X
Y

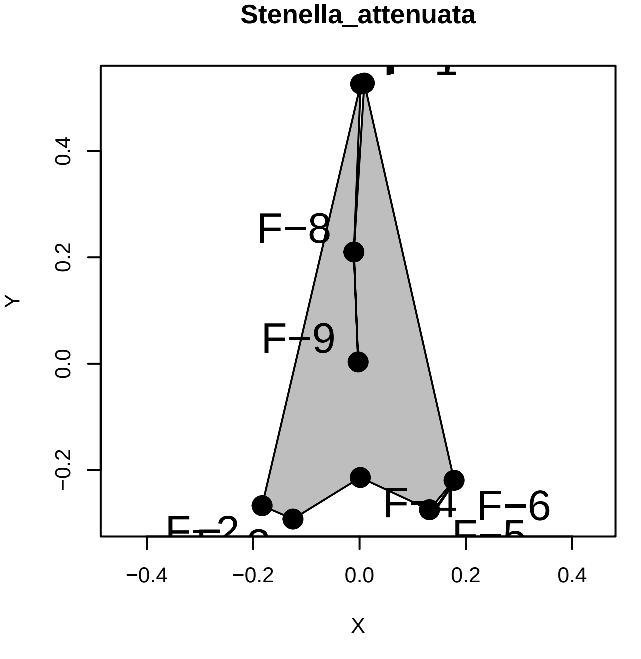
Stenella_attenuata
F−8
F−9
F−4
F−6
0.4
0.2
0.0
−0.2
−0.4
−0.2
0.0
0.2
0.4
X
Y

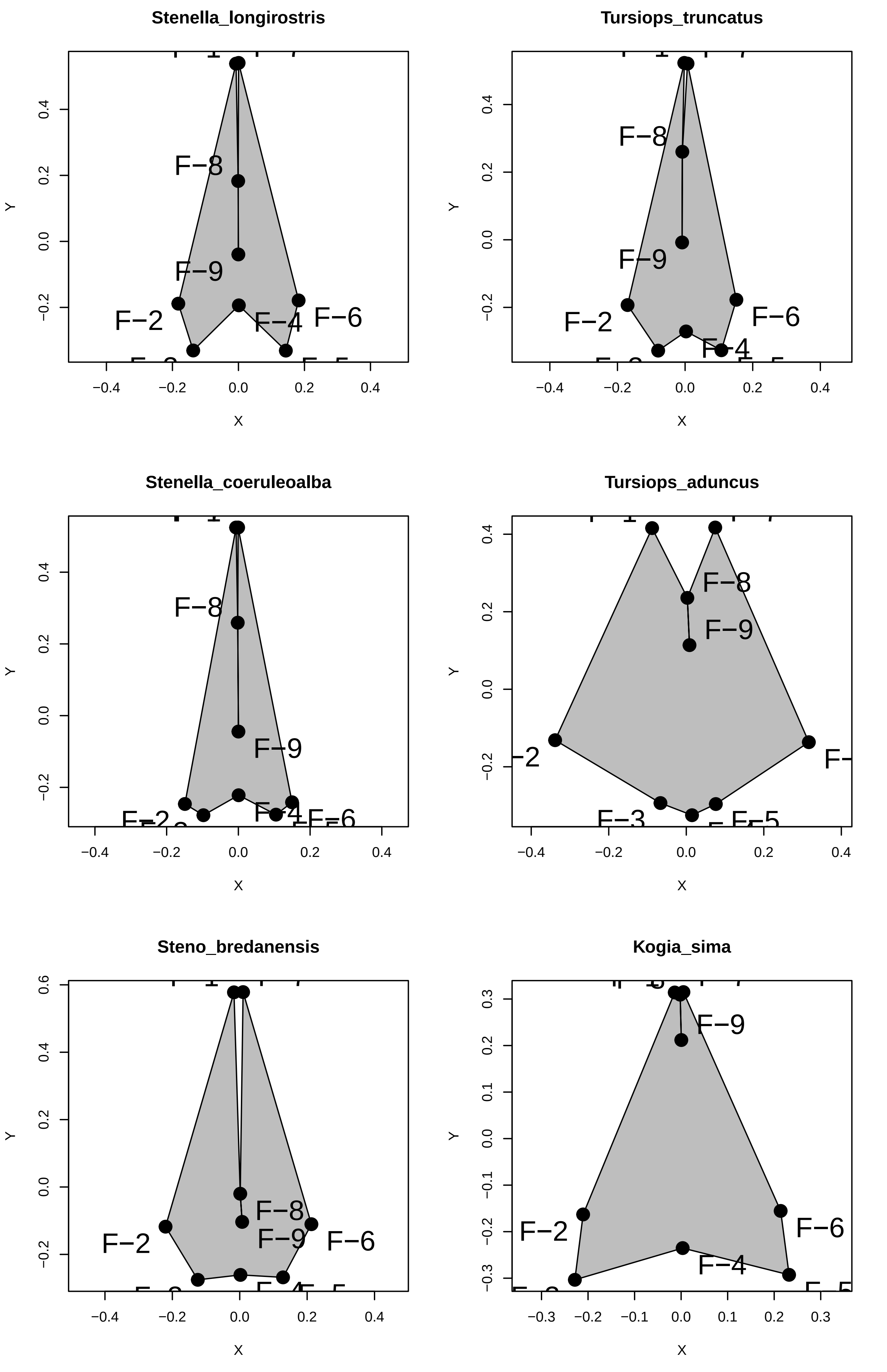
Stenella_longirostris
Tursiops_truncatus
Stenella_coeruleoalba
Tursiops_aduncus
Steno_bredanensis
Kogia_sima
F−2
F−4
F−6
F−8
F−9
F−3
F−5
X
Y
−0.4
−0.2
0.0
0.2
0.4
−0.3
−0.1
0.1
0.3
0.6

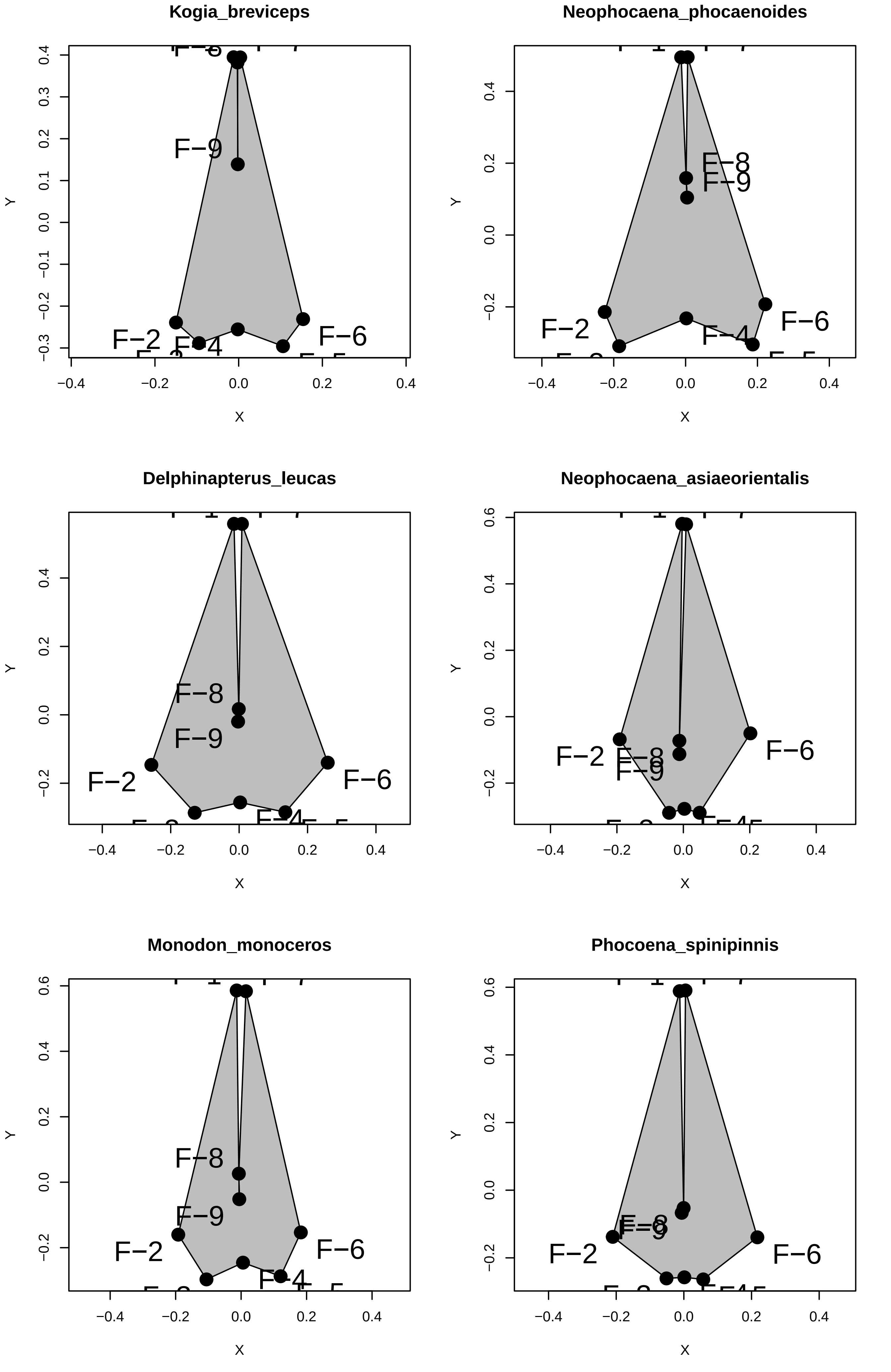

Kogia_breviceps
F−9
F−2
F−4
F−6
Neophocaena_phocaenoides
F−8
F−9
F−2
F−4
F−6
Delphinapterus_leucas
F−8
F−9
F−2
F−6
Neophocaena_asiaeorientalis
F−2
F−8
F−9
F−6
Monodon_monoceros
F−8
F−9
F−2
F−6
F−4
Phocoena_spinipinnis
F−2
F−6
X
Y

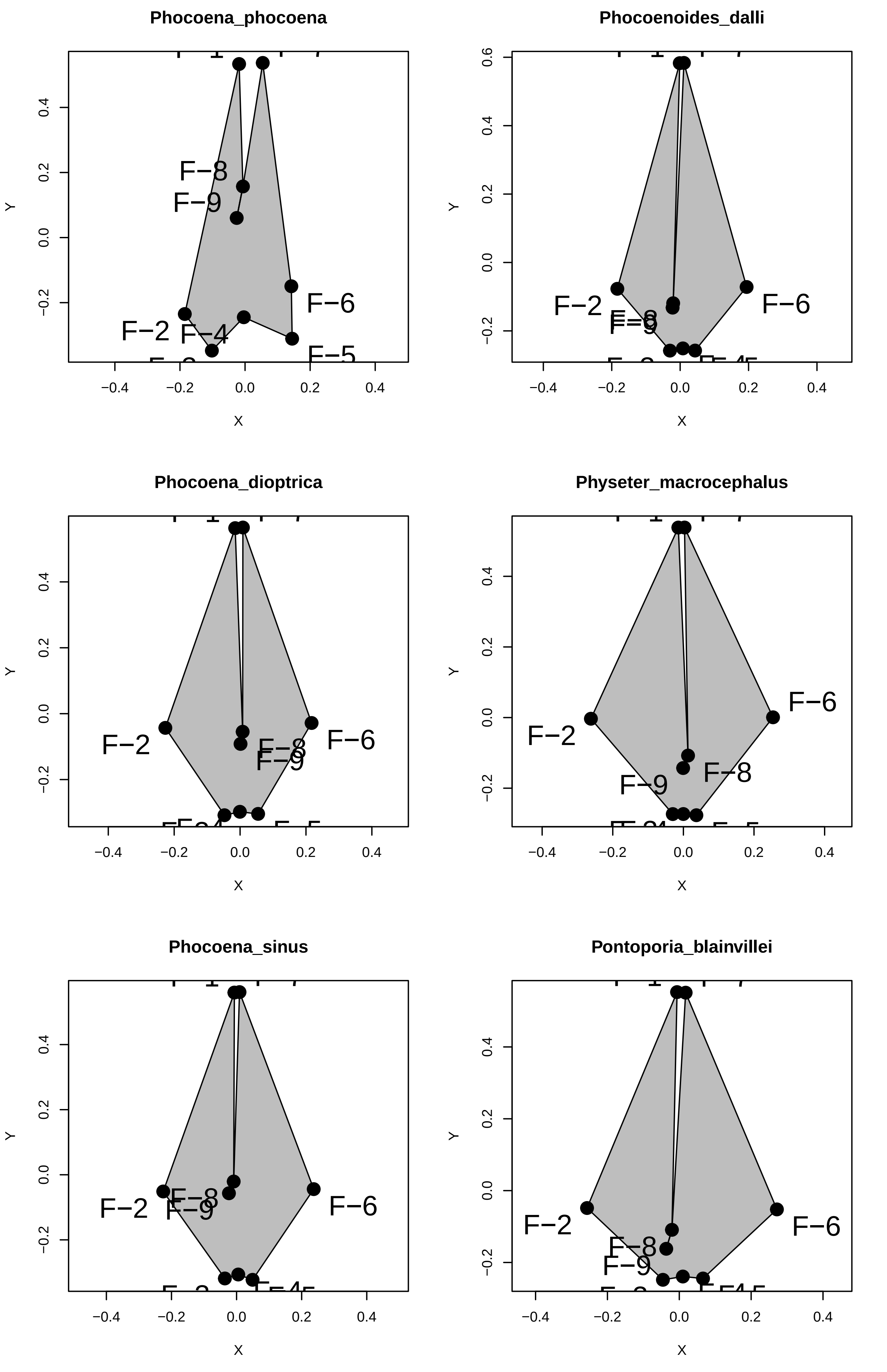
Phocoena_phocoena
F−8
F−9
F−6
F−2
F−4
F−5
Phocoenoides_dalli
F−2
F−6
Phocoena_dioptrica
F−2
F−6
F−8
F−9
Physeter_macrocephalus
F−6
F−2
F−8
F−9
Phocoena_sinus
F−2
F−8
F−9
F−6
Pontoporia_blainvillei
F−2
F−6
F−8
F−9
X
Y

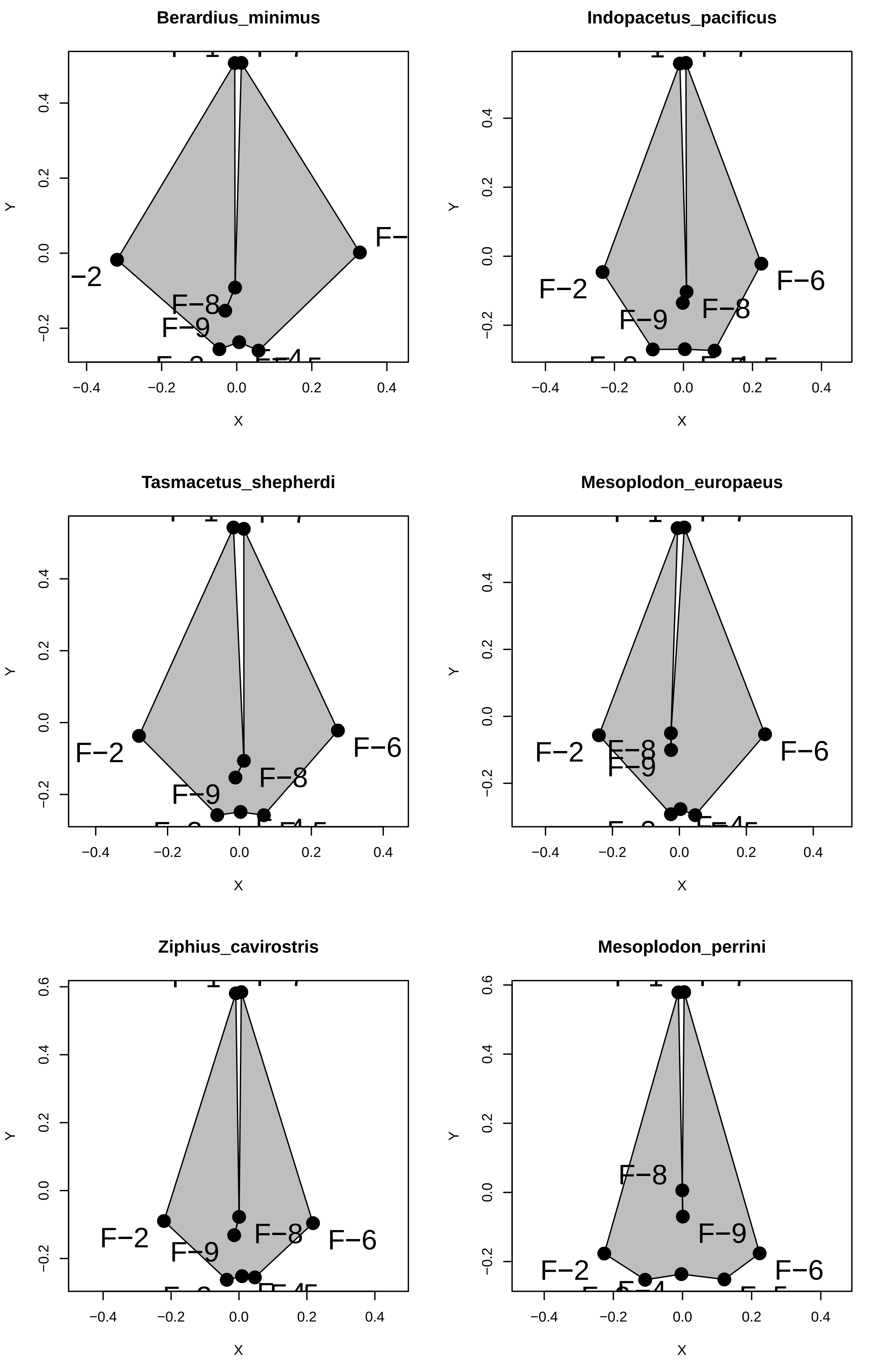

Berardius_minimus
−2
F−8
F−9
Indopacetus_pacificus
F−2
F−6
F−8
F−9
Tasmacetus_shepherdi
F−2
F−6
F−8
F−9
Mesoplodon_europaeus
F−2
F−8
F−9
F−6
Ziphius_cavirostris
F−2
F−8
F−6
F−9
Mesoplodon_perrini
F−8
F−9
F−2
F−6
X
Y

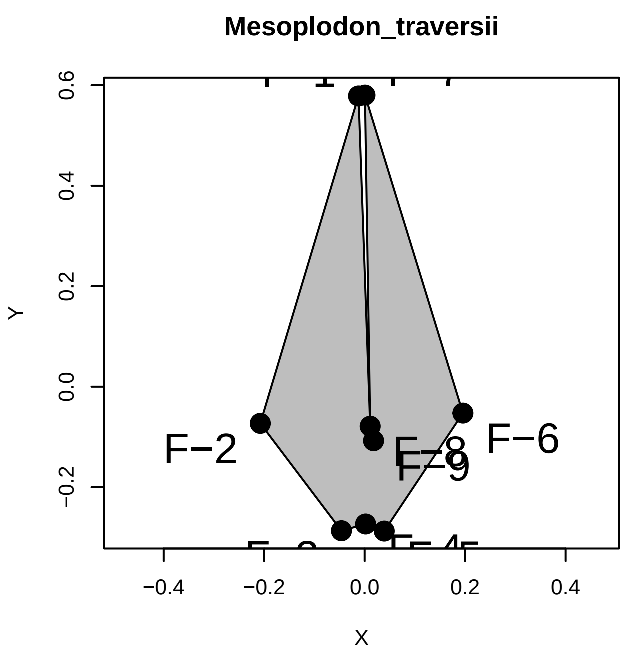
Mesoplodon_traversii
F−2
F−6
F−8
F−9
Y
X

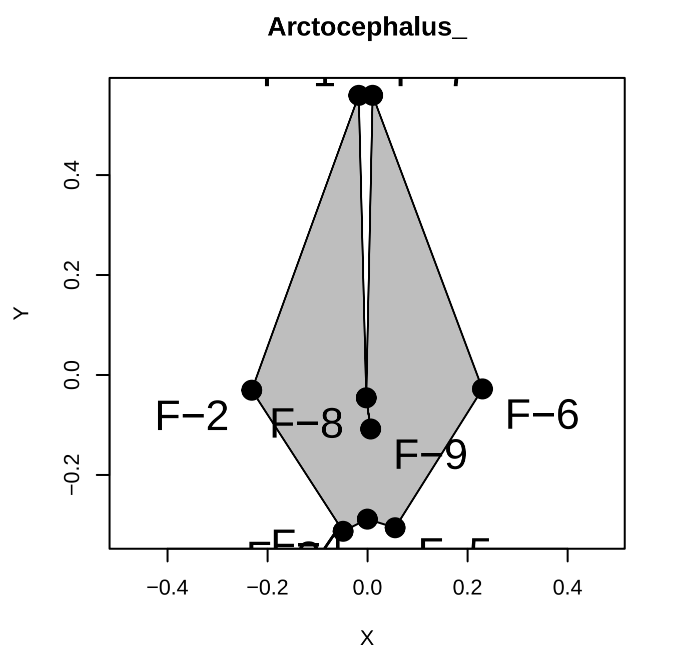
Arctocephalus_
F−2
F−8
F−6
F−9
Y
X

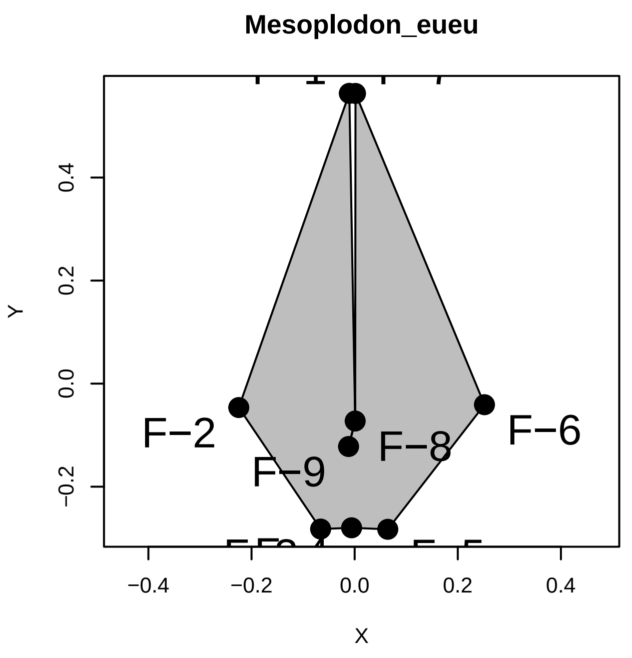
Mesoplodon_eueu
F−2
F−6
F−8
F−9
Y
X

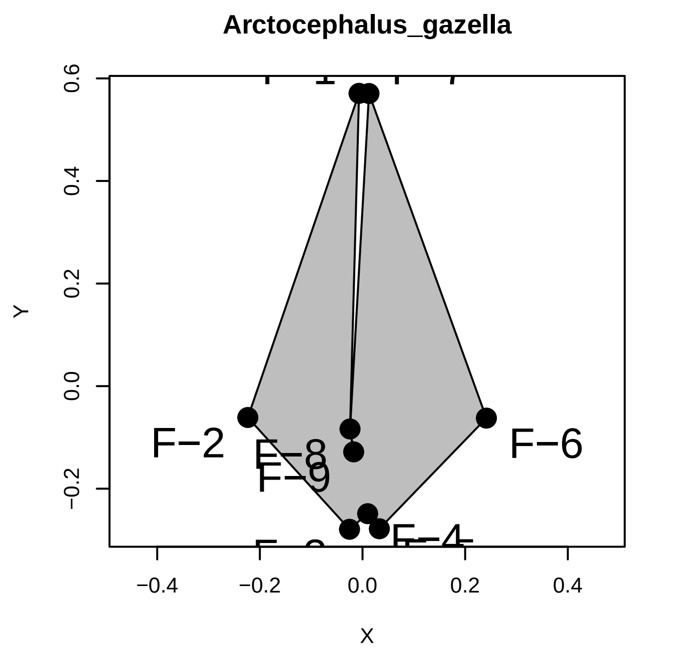
Arctocephalus_gazella
F−2
F−6
F−9
Y
X

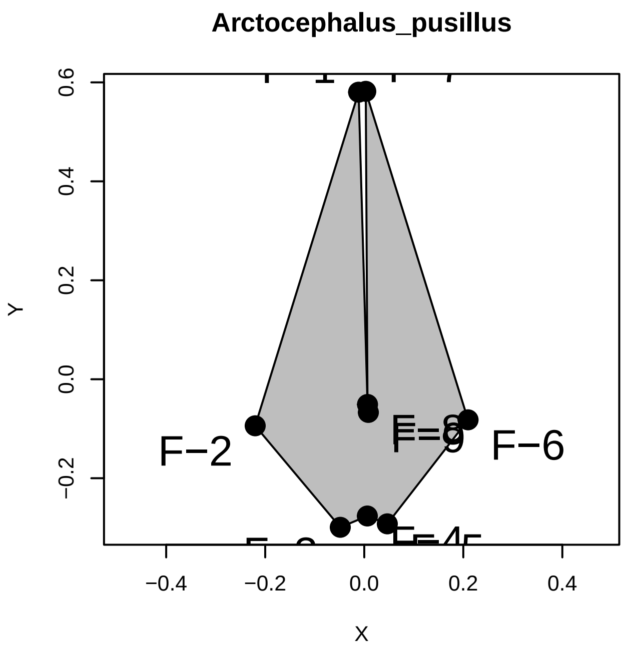
Arctocephalus_pusillus
F−2
F−6
Y
X

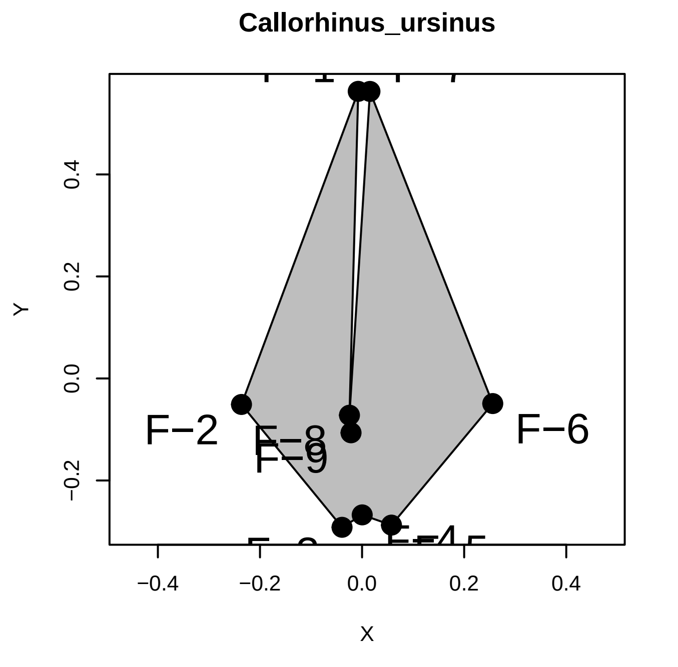
Callorhinus_ursinus
F−2
F−6
F−9
Y
X

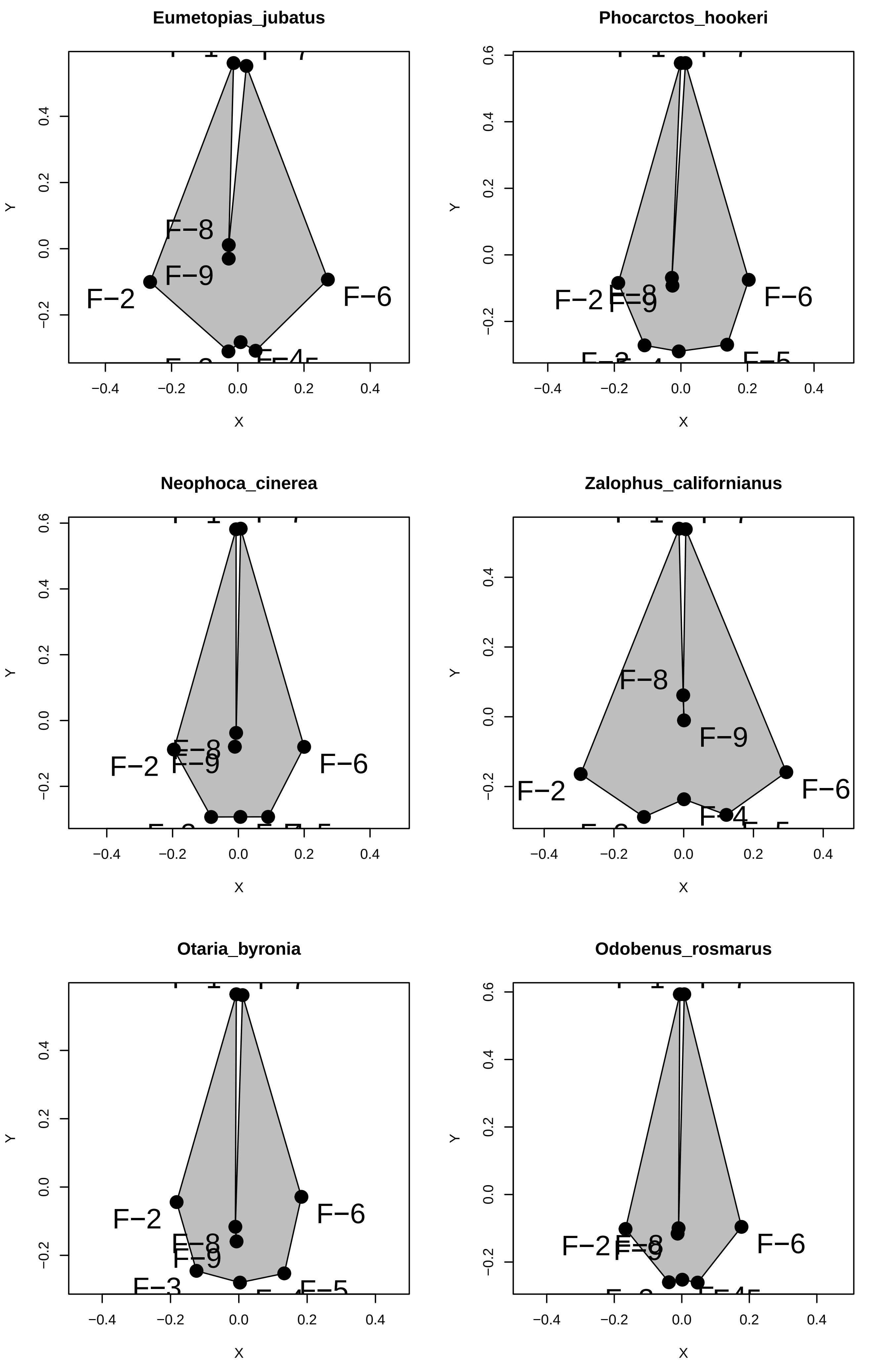

Eumetopias_jubatus
F−8
F−9
F−2
F−6
Phocarctos_hookeri
F−2
F−8
F−9
F−6
Neophoca_cinerea
F−8
F−9
F−2
F−6
Zalophus_californianus
F−8
F−9
F−2
F−6
F−4
Otaria_byronia
F−2
F−6
F−8
F−9
F−3
F−5
Odobenus_rosmarus
F−2
F−8
F−9
F−6
X
Y
−0.4
−0.2
0.0
0.2
0.4
0.6

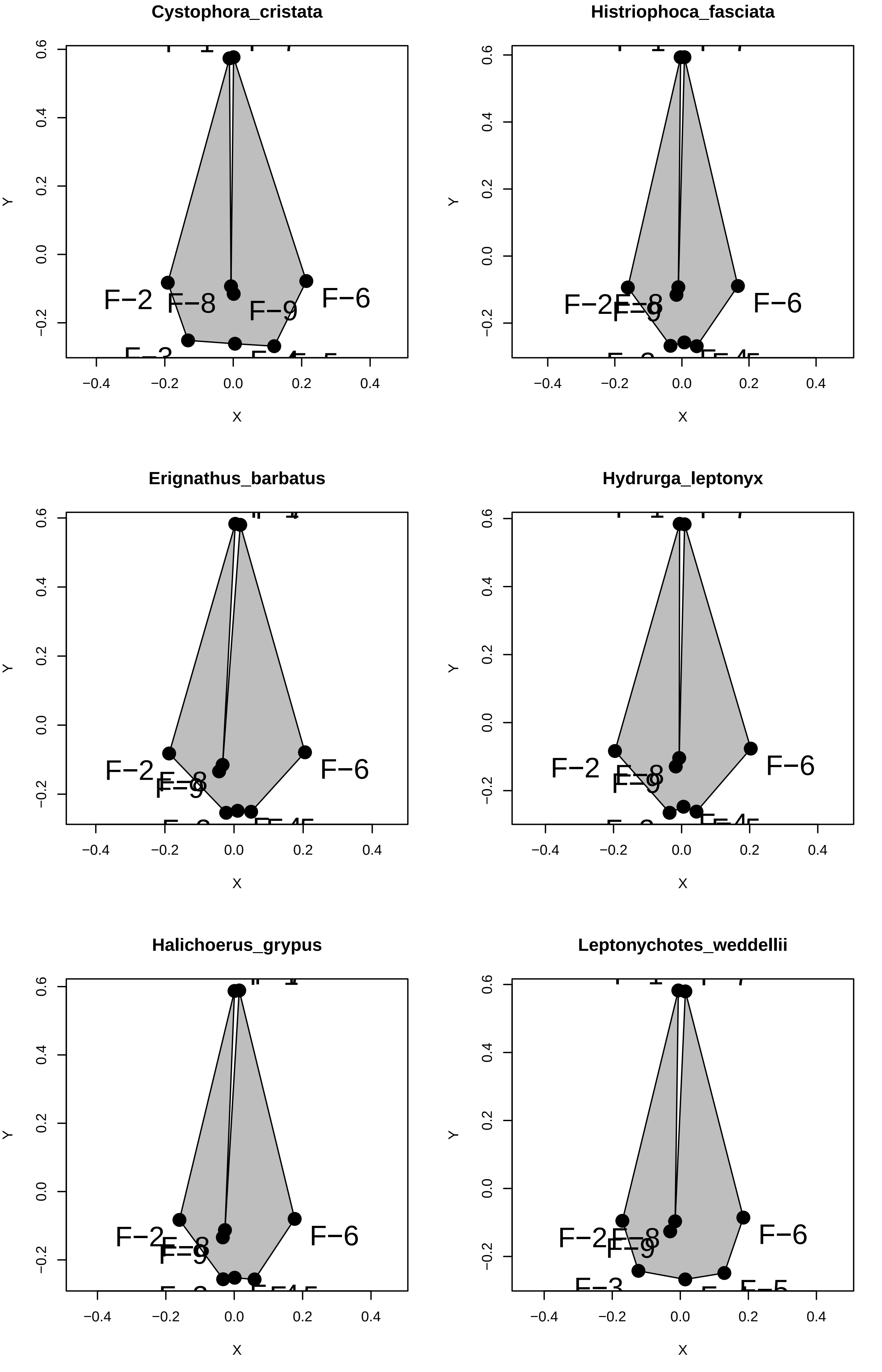
Cystophora_cristata
Histriophoca_fasciata
Erignathus_barbatus
Hydrurga_leptonyx
Halichoerus_grypus
Leptonychotes_weddellii
X
Y
F−2
F−3
F−5
F−6
F−8
F−9
0.6
0.4
0.2
0.0
−0.2
−0.4

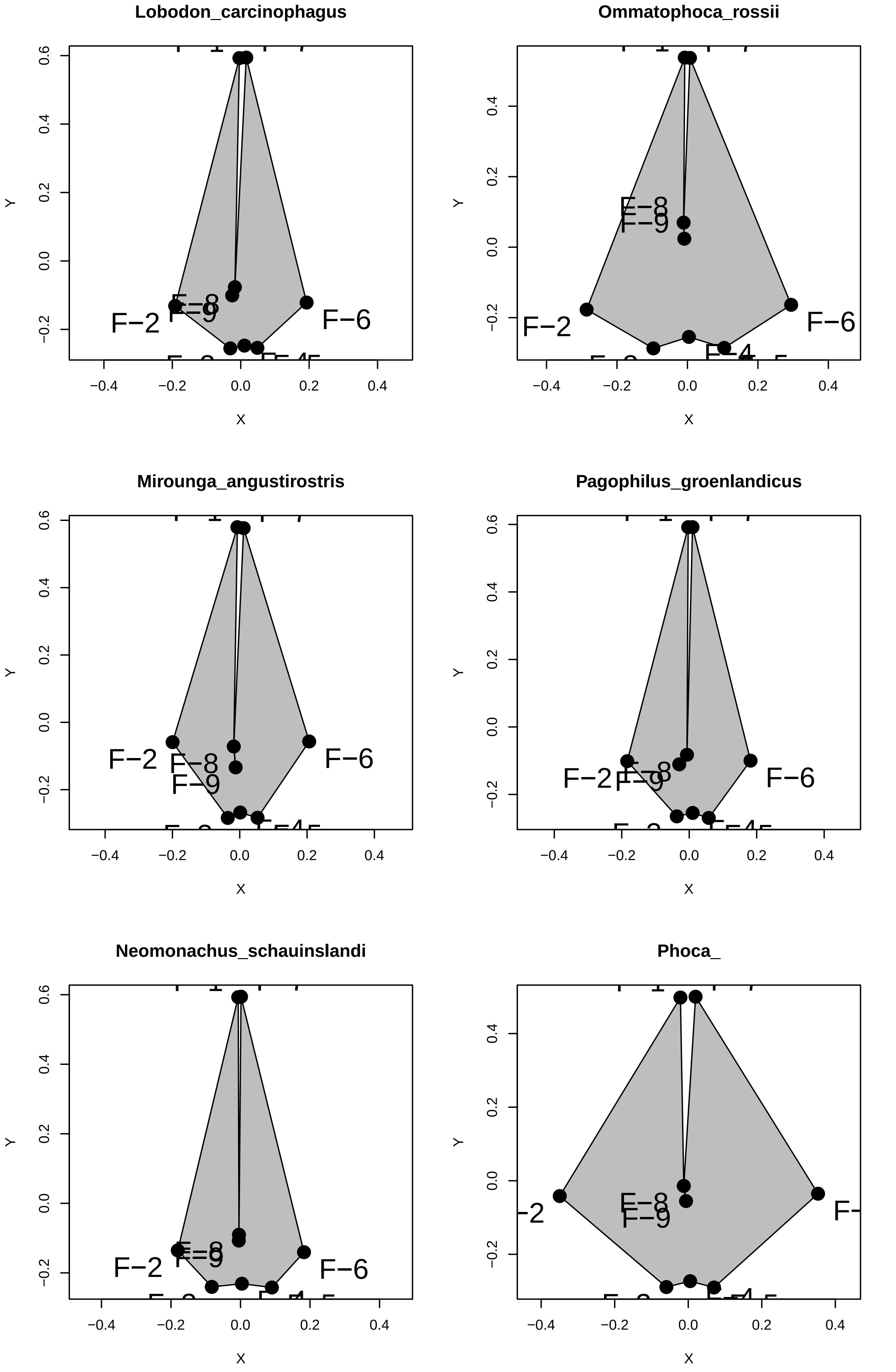
Lobodon_carcinophagus
Ommatophoca_rossii
Mirounga_angustirostris
Pagophilus_groenlandicus
Neomonachus_schauinslandi
Phoca_
F−2
F−6
F−8
F−9
X
Y

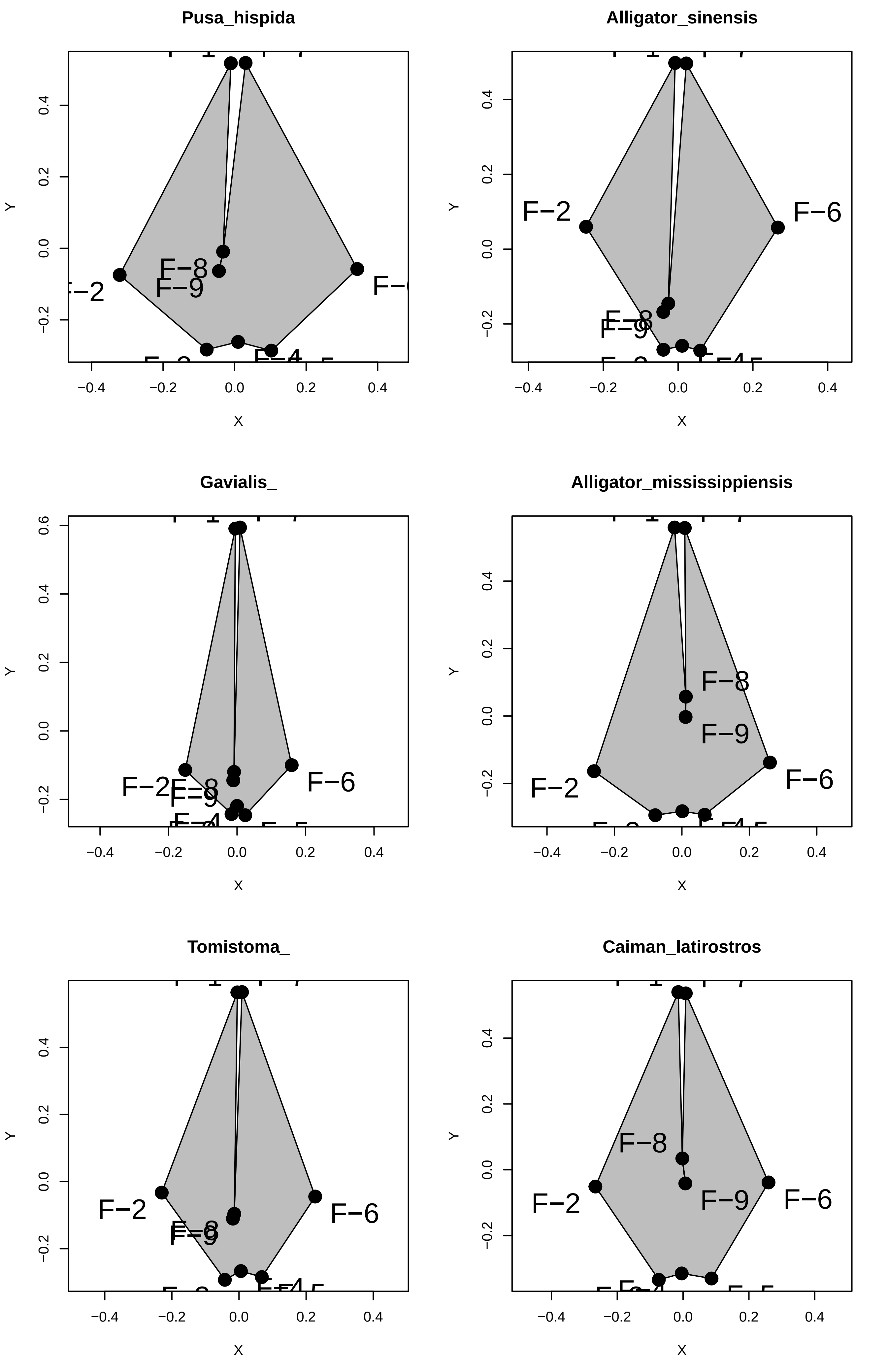

Pusa_hispida
F−8
F−9
Alligator_sinensis
F−2
F−6
Gavialis_
F−2
F−6
Alligator_mississippiensis
F−8
F−9
F−2
F−6
Tomistoma_
F−2
F−6
Caiman_latirostros
F−8
F−2
F−9
F−6
X
Y

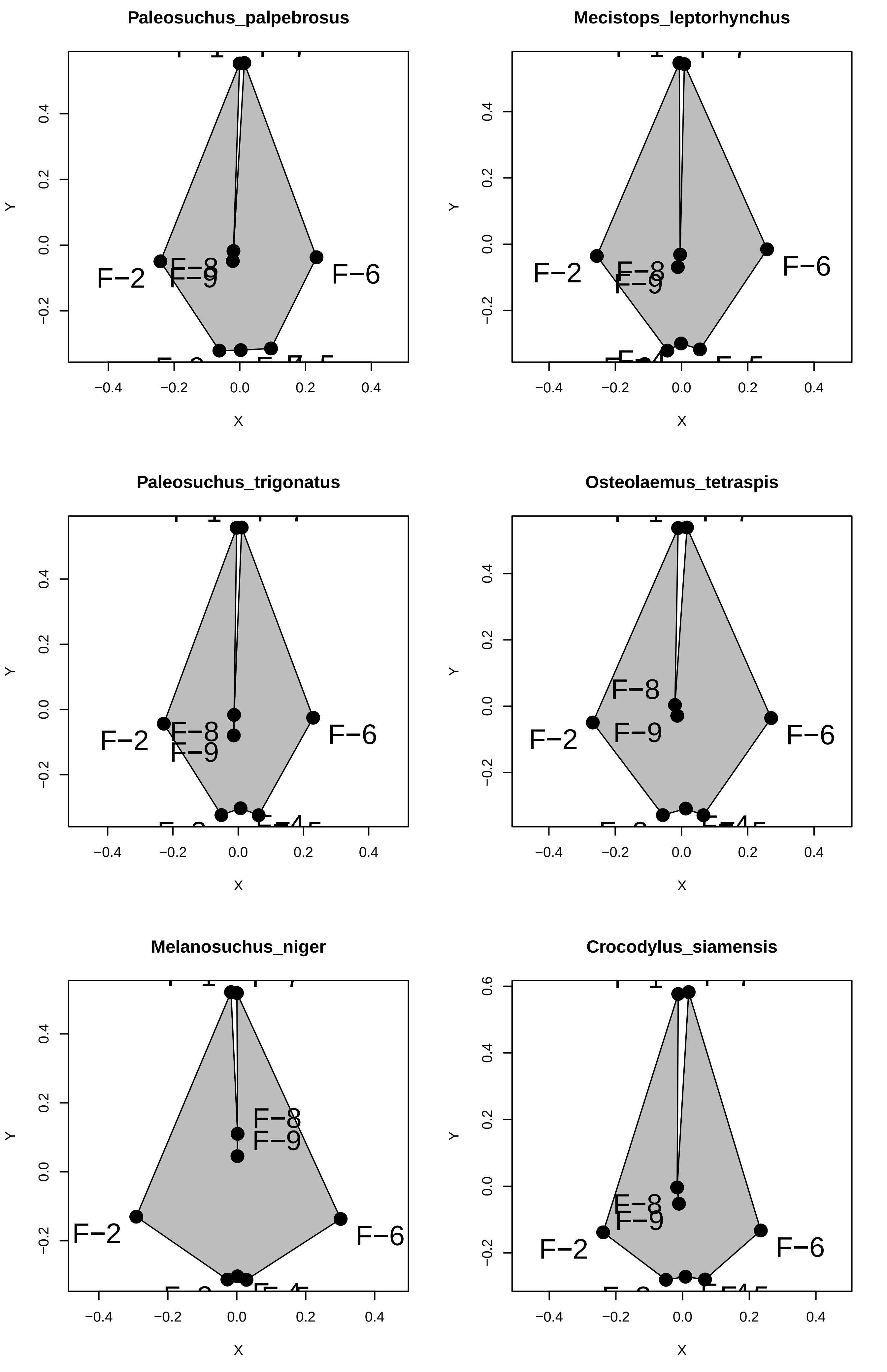
Paleosuchus_palpebrosus
Mecistops_leptorhynchus
Paleosuchus_trigonatus
Osteolaemus_tetraspis
Melanosuchus_niger
Crocodylus_siamensis
F−2
F−6
F−8
F−9
X
Y

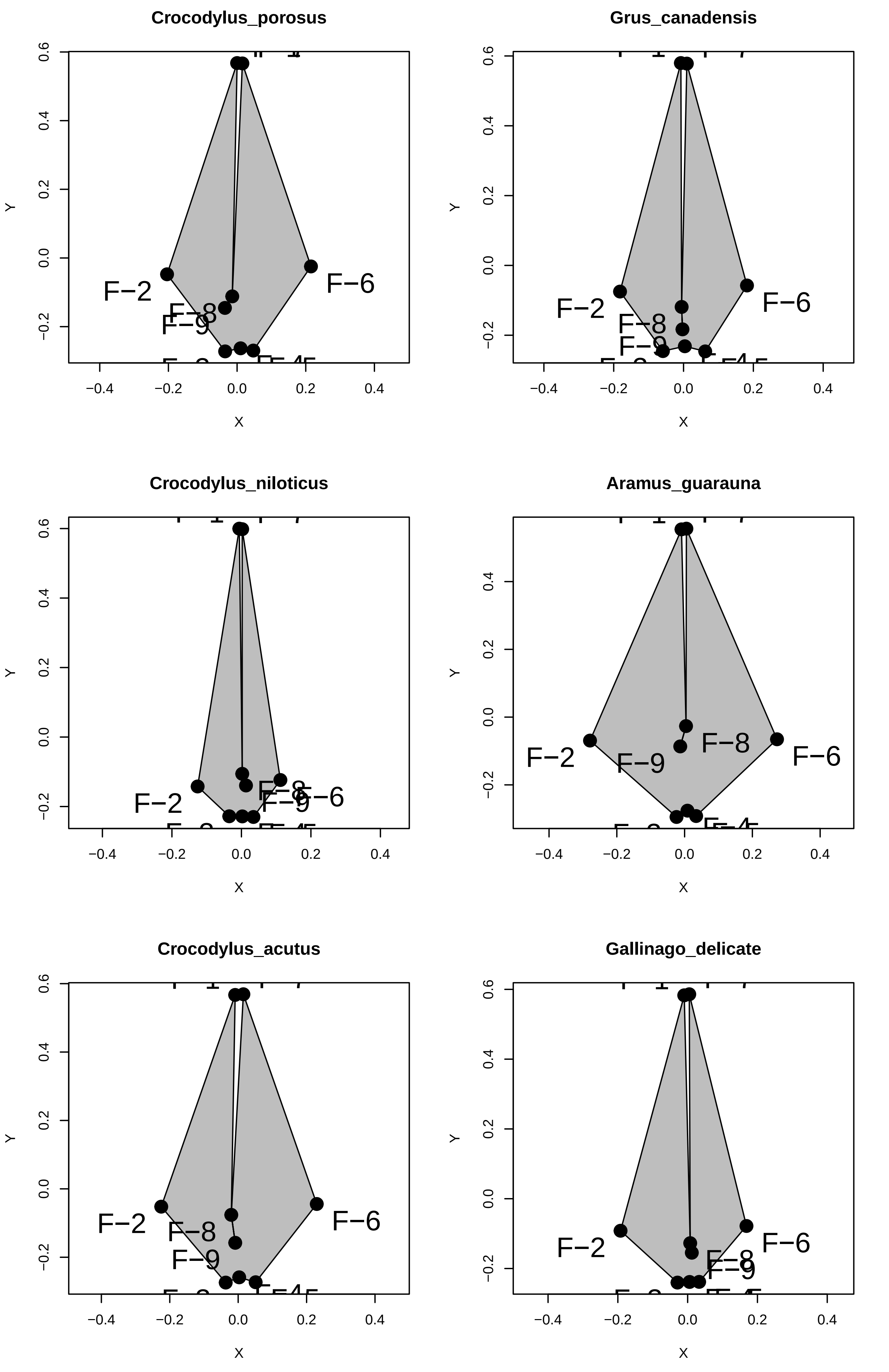
Crocodylus_porosus
Grus_canadensis
Crocodylus_niloticus
Aramus_guarauna
Crocodylus_acutus
Gallinago_delicate
F−2
F−6
F−8
F−9
X
Y

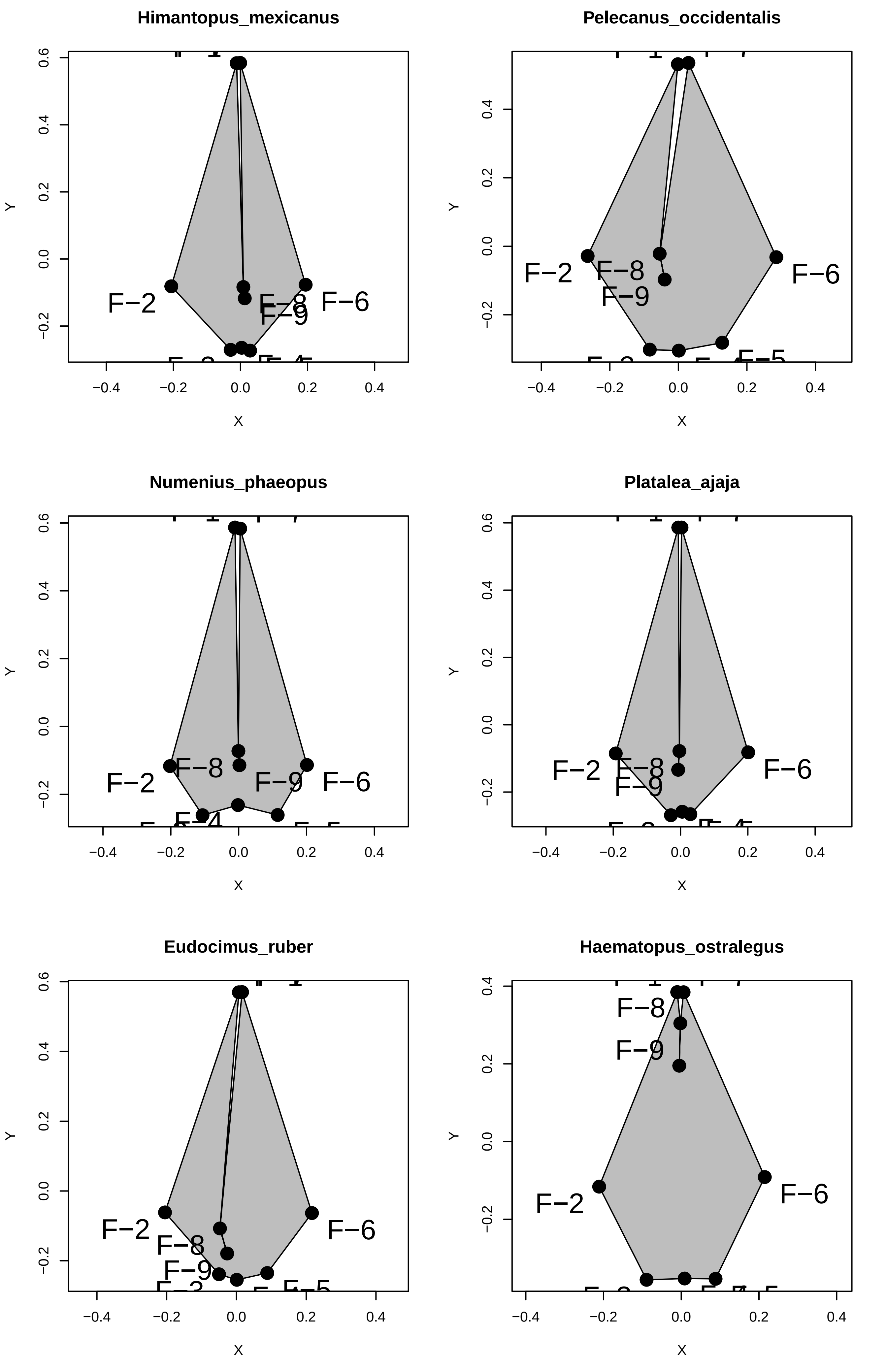
Himantopus_mexicanus
F−2
F−8
F−9
F−6
Pelecanus_occidentalis
F−2
F−8
F−9
F−6
F−5
Numenius_phaeopus
F−2
F−8
F−9
F−6
Platalea_ajaja
F−2
F−8
F−9
F−6
Eudocimus_ruber
F−2
F−8
F−9
F−6
Haematopus_ostralegus
F−8
F−9
F−2
F−6
X
Y

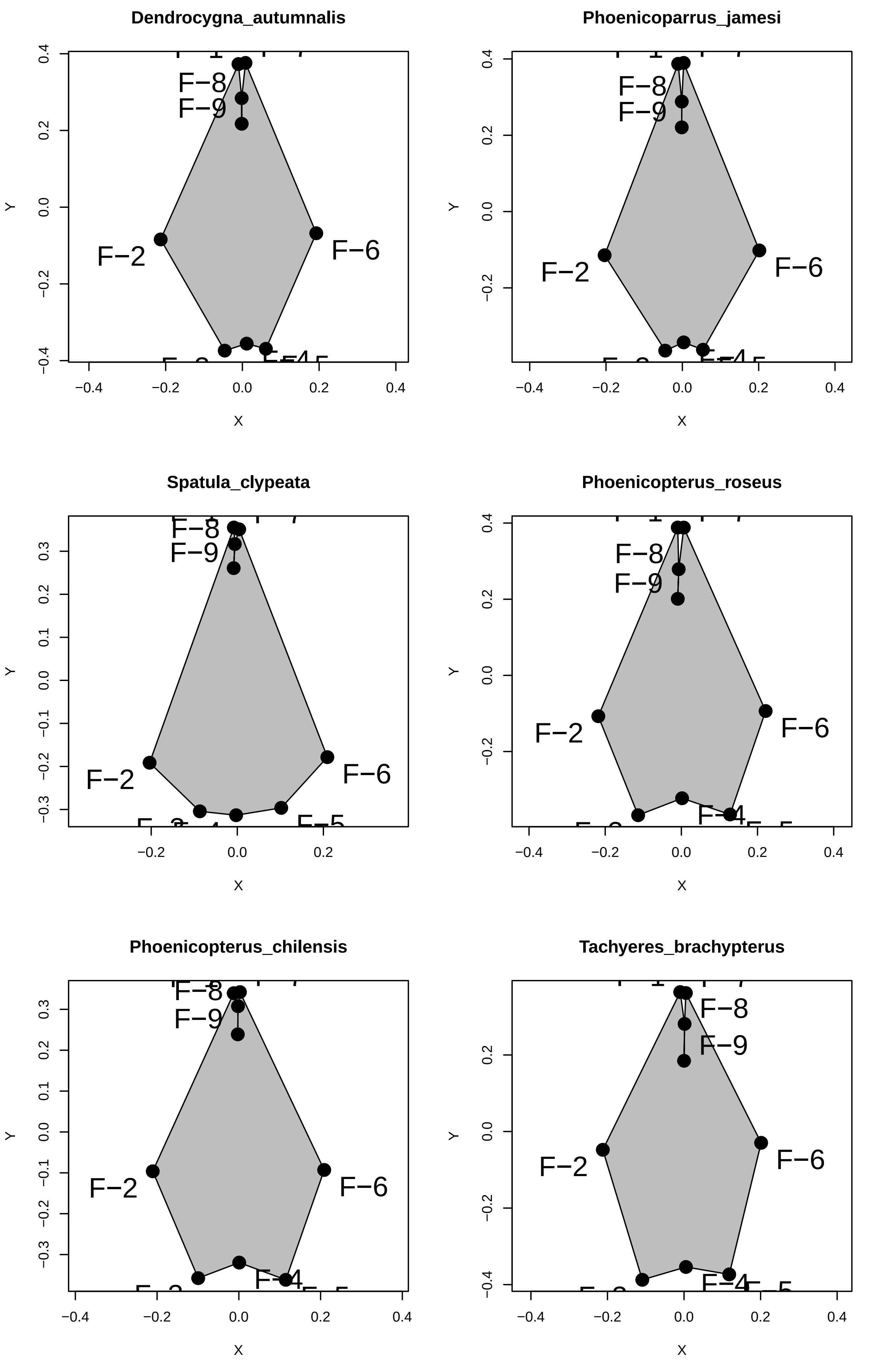

Dendrocygna_autumnalis
F−8
F−9
F−2
F−6
Phoenicoparrus_jamesi
F−8
F−9
F−2
F−6
Spatula_clypeata
F−8
F−9
F−2
F−6
F−5
Phoenicopterus_roseus
F−8
F−9
F−2
F−6
F−4
Phoenicopterus_chilensis
F−8
F−9
F−2
F−6
F−4
Tachyeres_brachypterus
F−8
F−9
F−2
F−6
F−4
X
Y

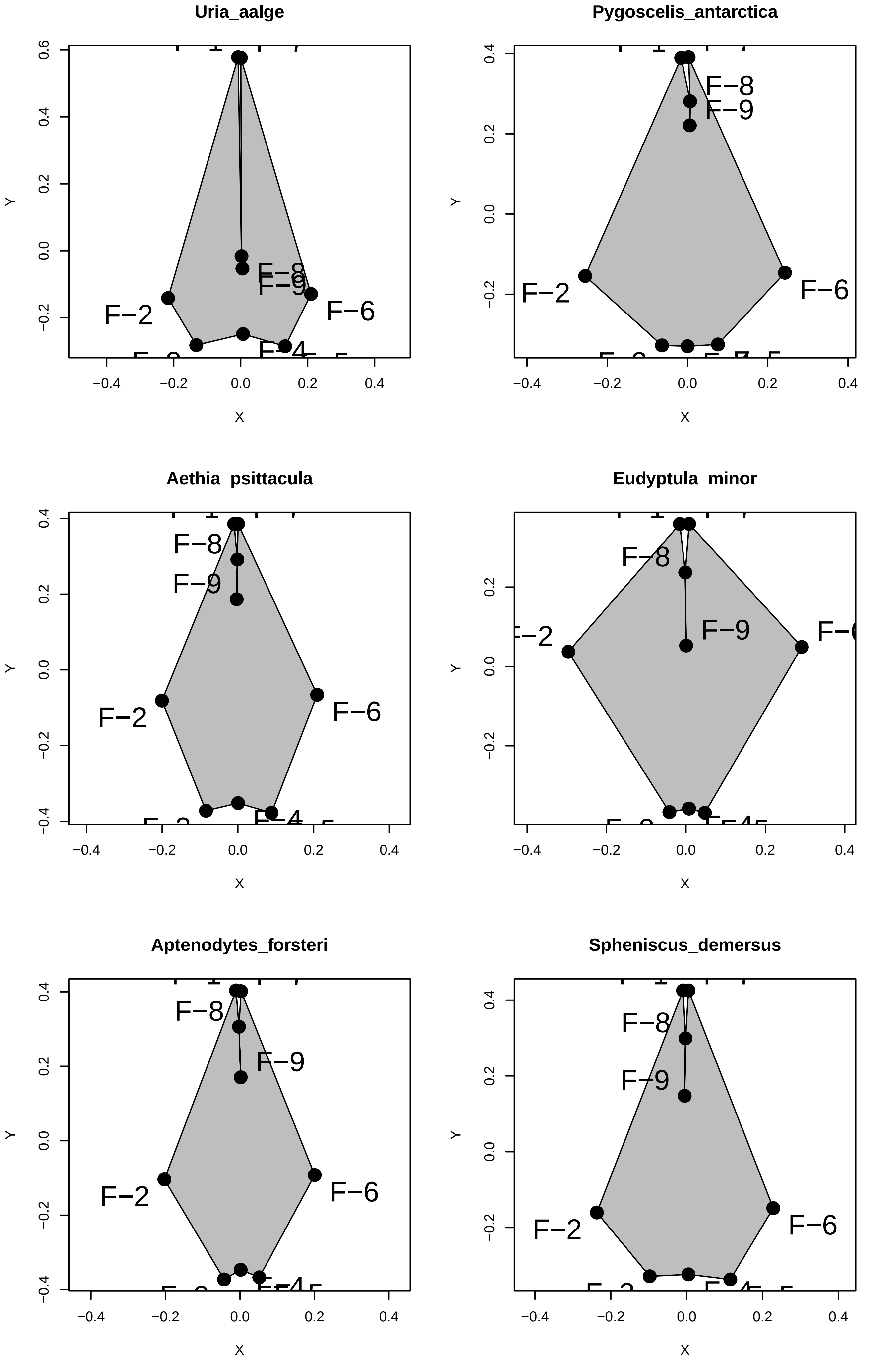
Uria_aalge
Pygoscelis_antarctica
Aethia_psittacula
Eudyptula_minor
Aptenodytes_forsteri
Spheniscus_demersus
F−2
F−6
F−8
F−9
X
Y

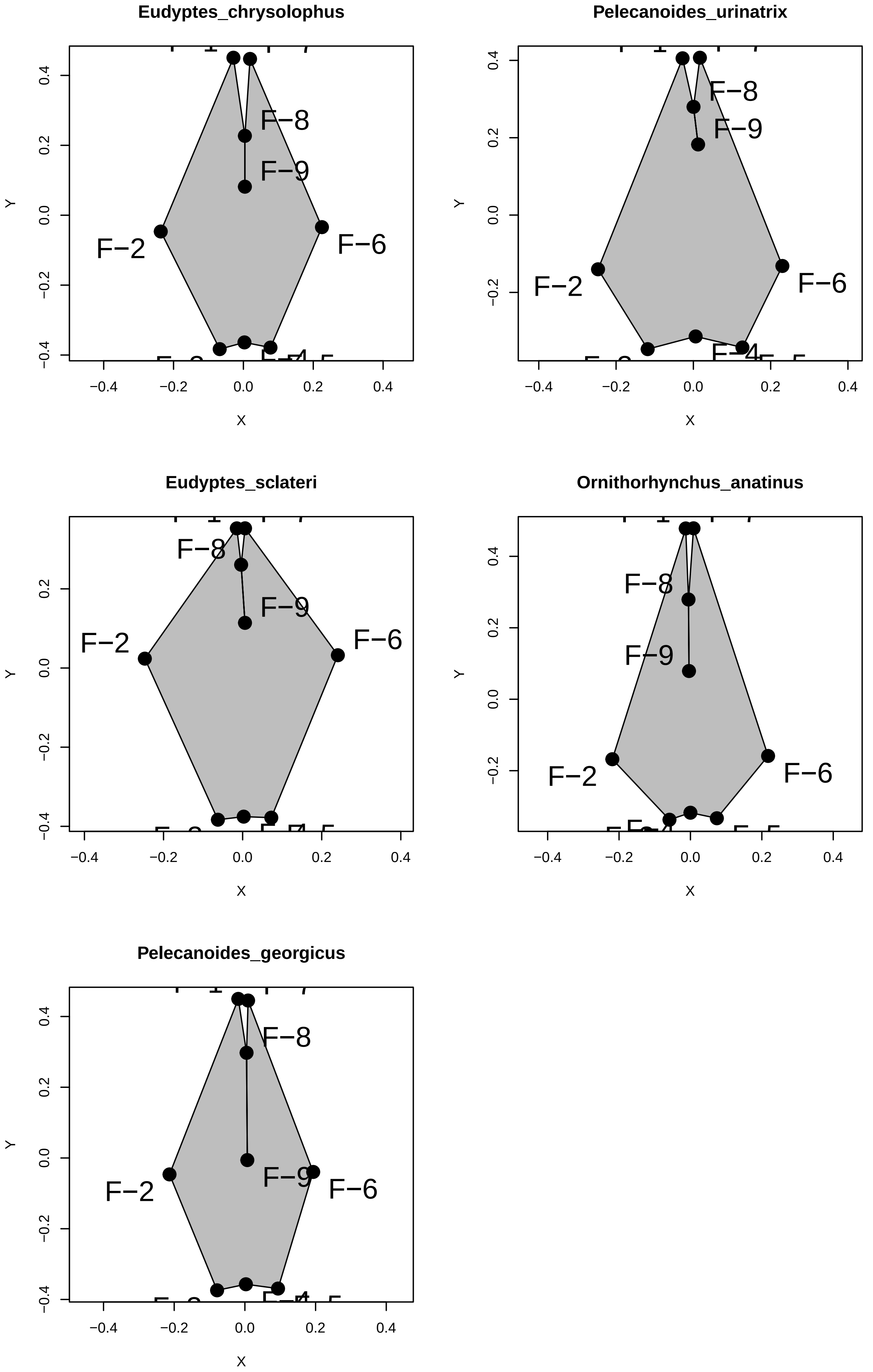
Eudyptes_chrysolophus
Pelecanoides_urinatrix
Eudyptes_sclateri
Ornithorhynchus_anatinus
Pelecanoides_georgicus
F−2
F−6
F−8
F−9
X
Y
−0.4
−0.2
0.0
0.2
0.4
