# Supplementary material for: Was Hupehsuchus a baleen whale-style filter feeder in the Early Triassic? A re-examination of the evidence
Source: PeerJ. 2025 Jul 4;13:e19666. doi: 10.7717/peerj.19666 (PMC12232927; doi:10.7717/peerj.19666)
Supplement: Supplemental Information 5 — The first column is for the new homologous set of landmarks taken from 3D cranial models. The second column is for the landmarks as defined by Fang et a l. (2023) but were placed by us on the 3D cranial models. The third column is the landmarks as published by Fang et al. (2023). [file peerj-13-19666-s005.pdf]

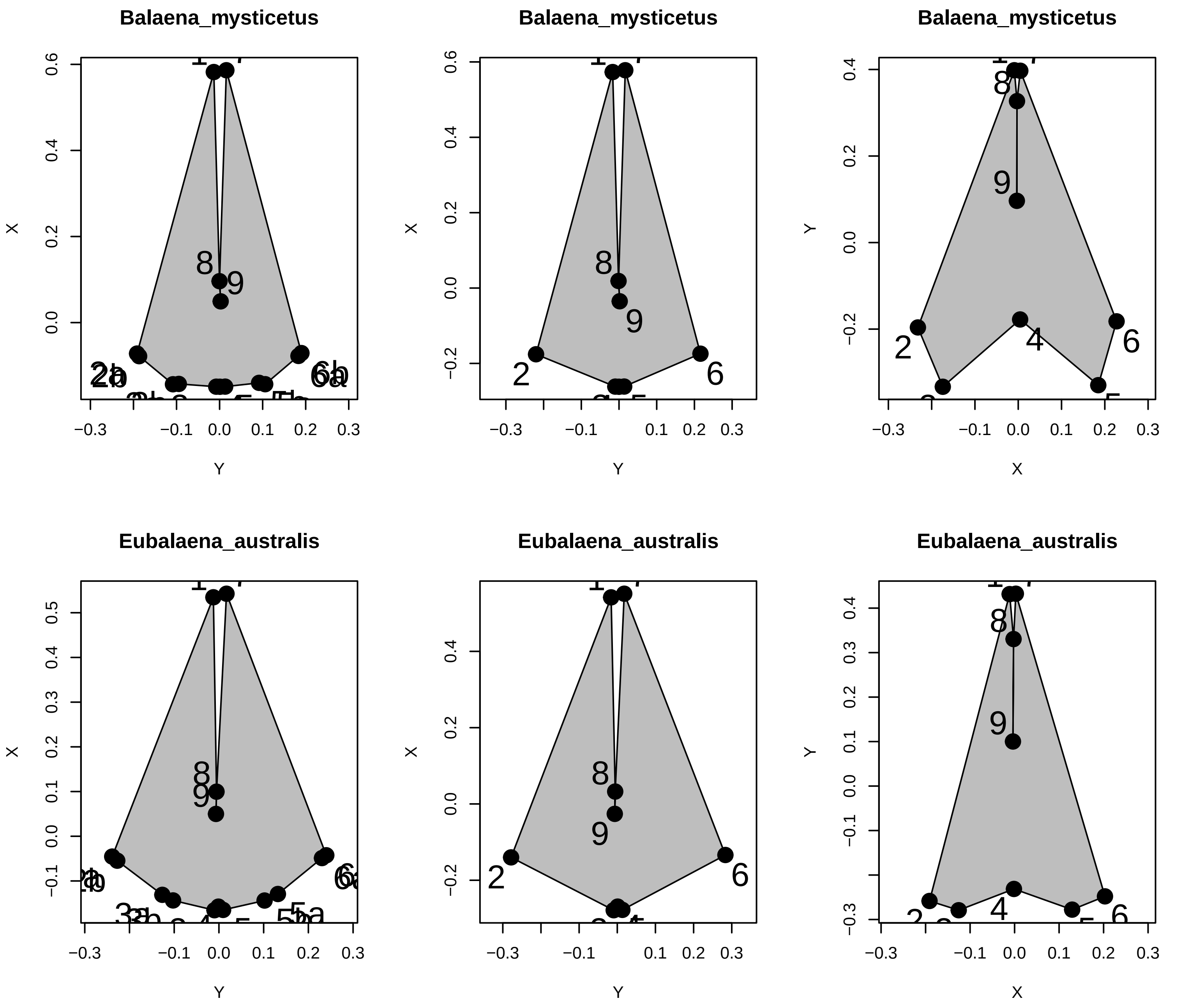

Balaena_mysticetus
Balaena_mysticetus
Balaena_mysticetus
Eubalaena_australis
Eubalaena_australis
Eubalaena_australis
X
Y
X
Y
Y
X
X
Y
X
Y
Y
X
1
7
8
9
2
4
6

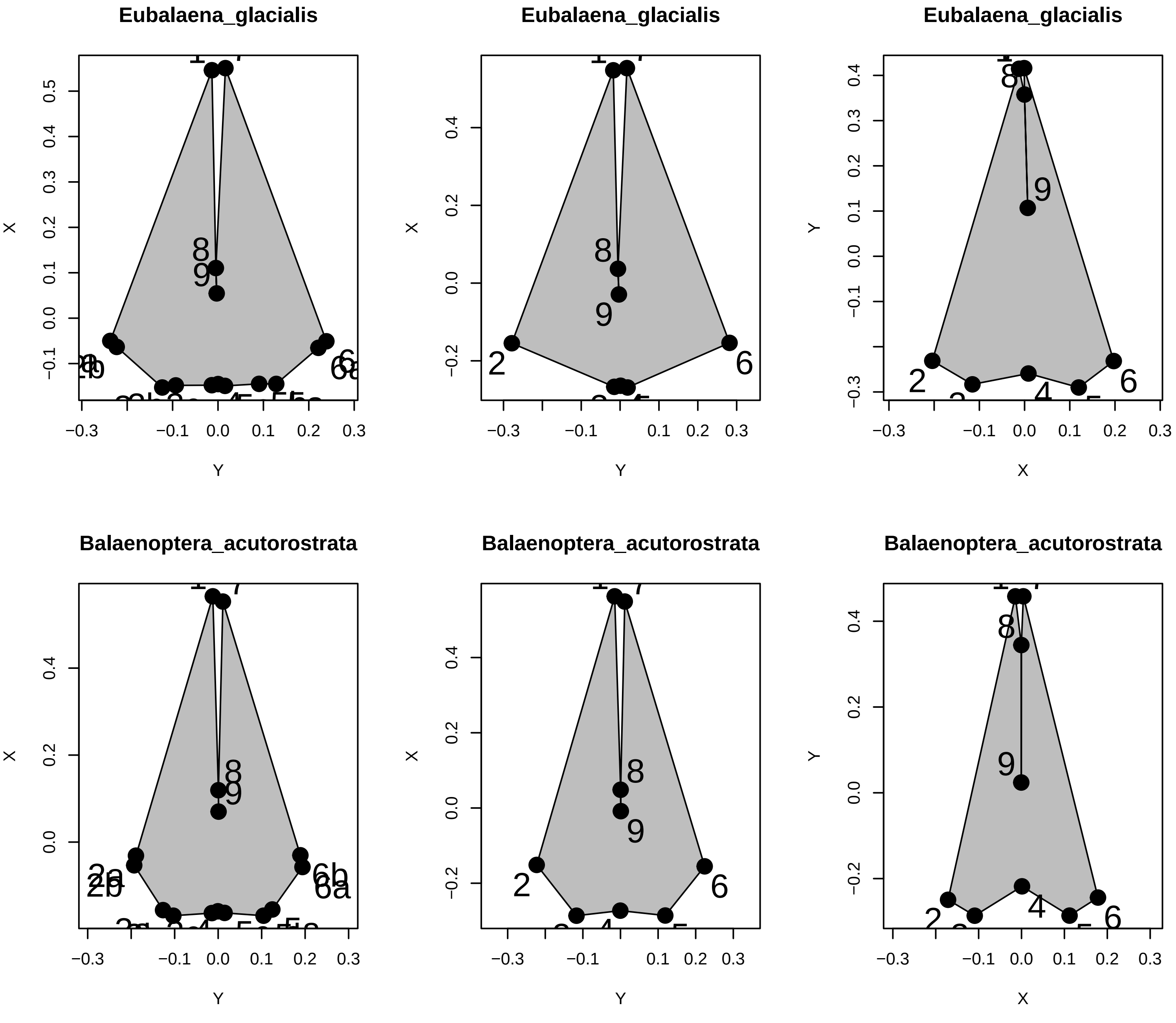
Eubalaena_glacialis
X
Y
Eubalaena_glacialis
X
Y
Eubalaena_glacialis
Y
X
Balaenoptera_acutorostrata
X
Y
Balaenoptera_acutorostrata
X
Y
Balaenoptera_acutorostrata
Y
X

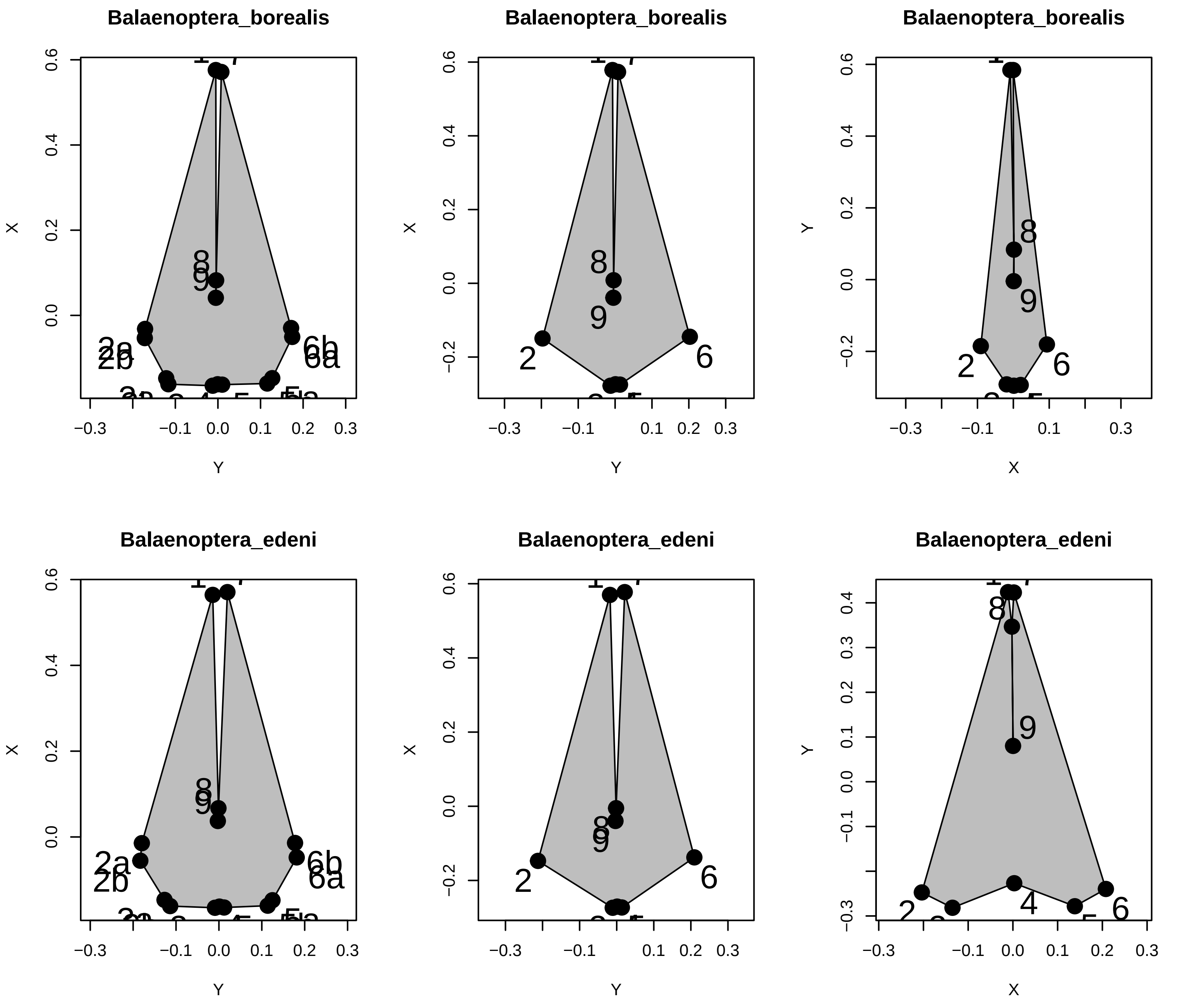
Balaenoptera_borealis
Balaenoptera_borealis
Balaenoptera_borealis
Balaenoptera_edeni
Balaenoptera_edeni
Balaenoptera_edeni
X
Y
X
Y
Y
X
X
Y
X
Y
Y
X

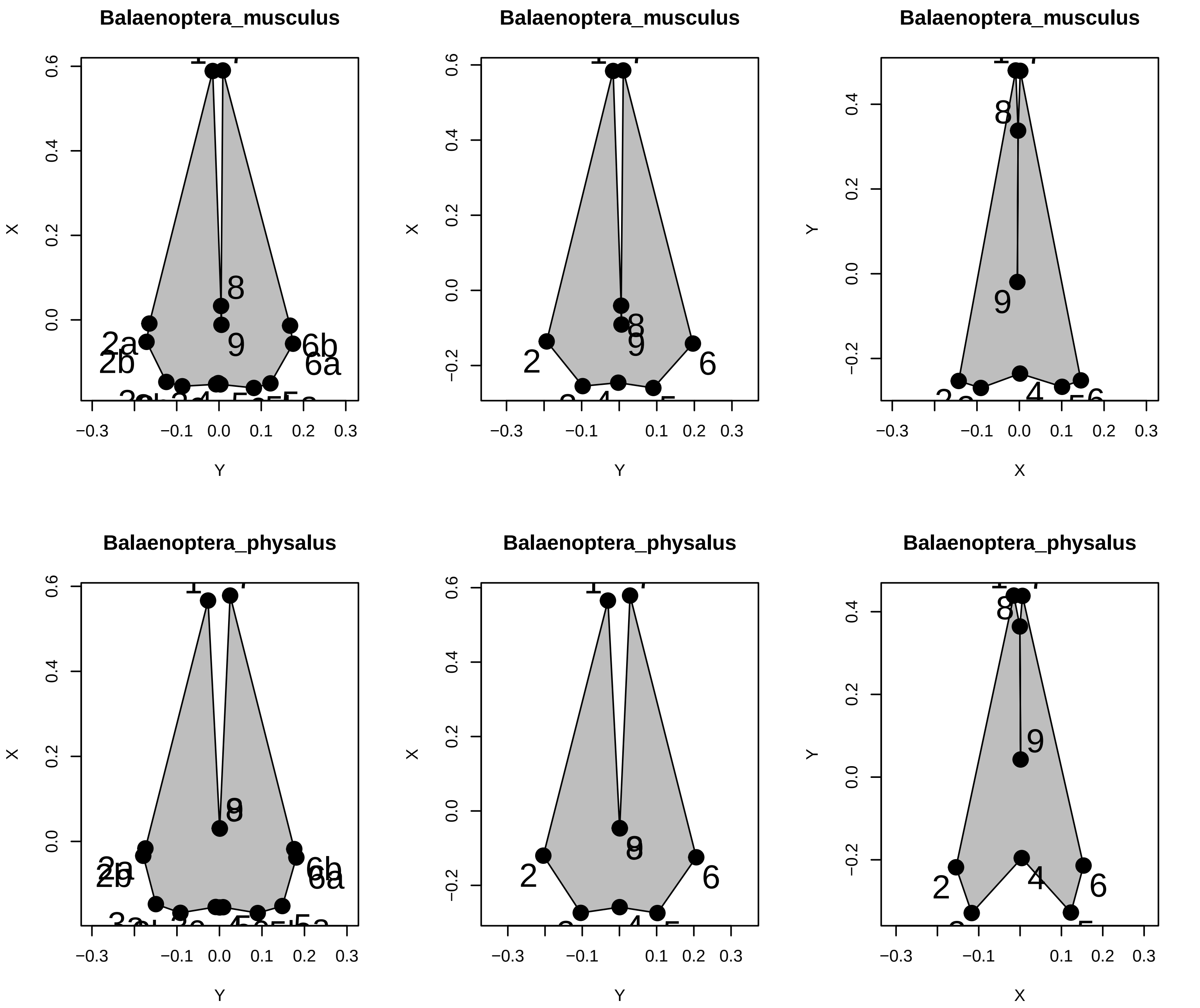
Balaenoptera_musculus
Balaenoptera_musculus
Balaenoptera_musculus
Balaenoptera_physalus
Balaenoptera_physalus
Balaenoptera_physalus
X
Y
X
Y
Y
X
X
Y
X
Y
Y
X
0.6
0.4
0.2
0.0
−0.2
−0.3
−0.1
0.1
0.2
0.3
2a
2b
6a
6b
8
9
2
6
4

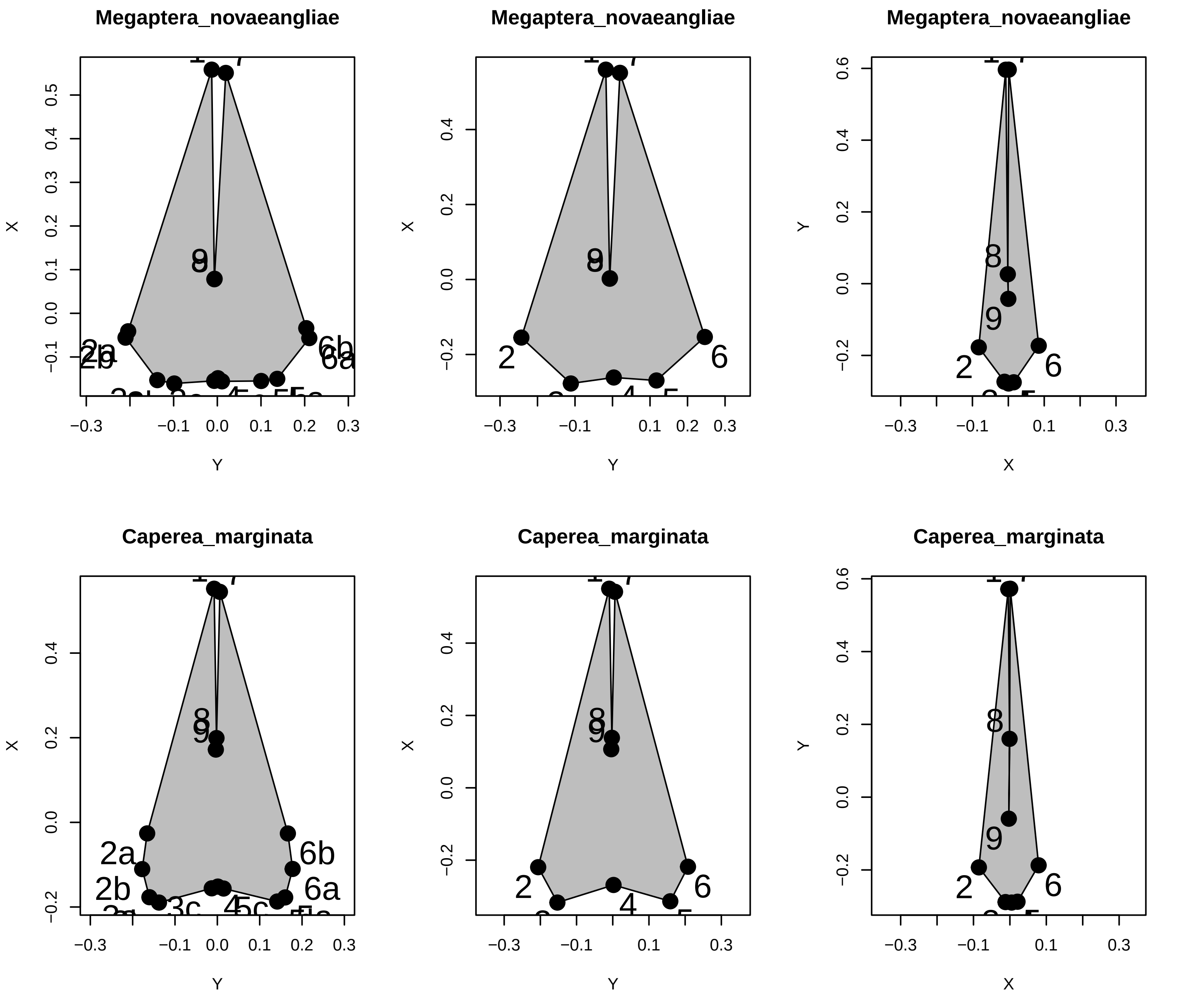
Megaptera_novaeangliae
Megaptera_novaeangliae
Megaptera_novaeangliae
Caperea_marginata
Caperea_marginata
Caperea_marginata
X
Y
2a
2b
6b
6a
9
2
6
8
4
2a
2b
6b
6a
3c
5c

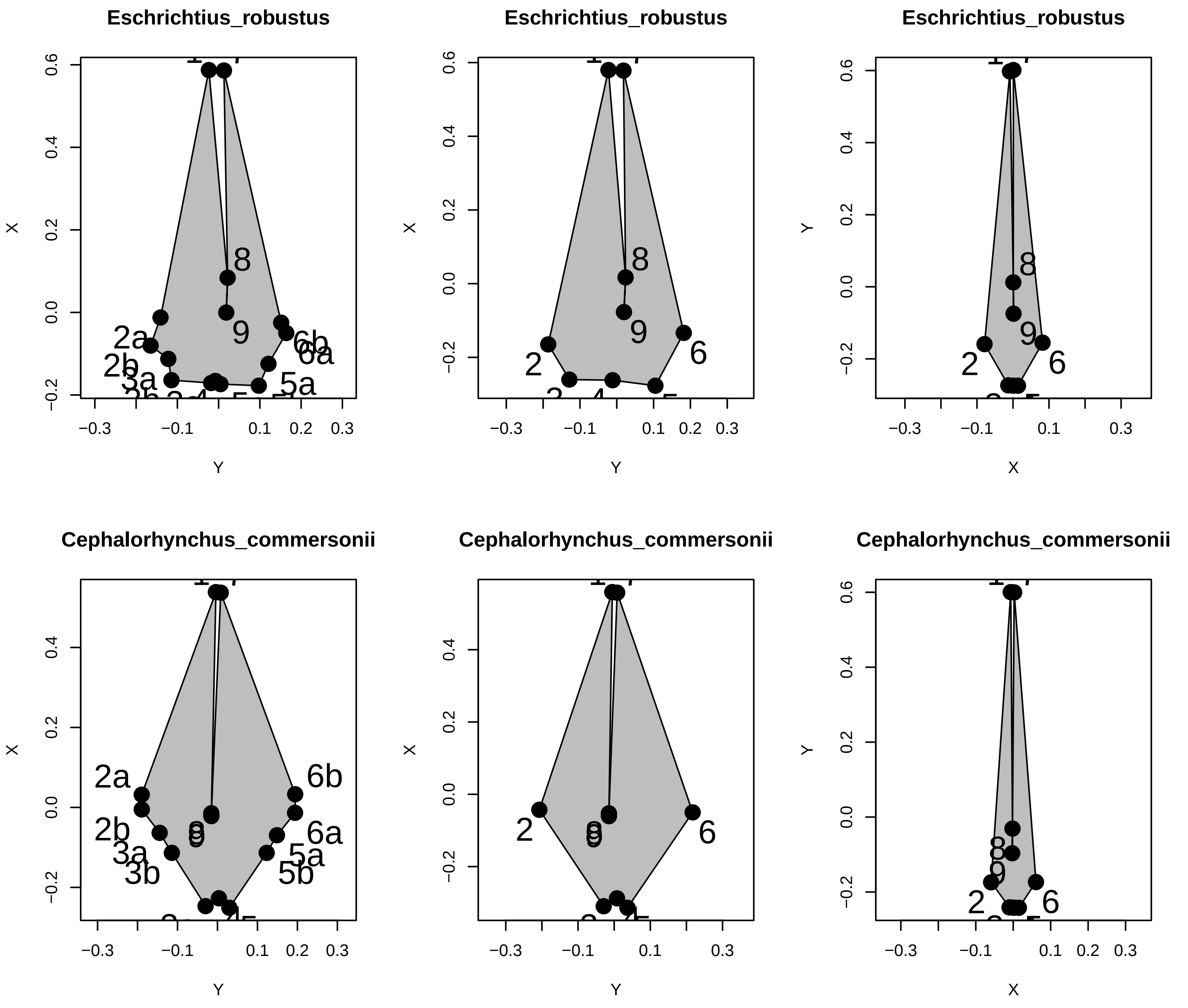
Eschrichtius_robustus
Eschrichtius_robustus
Eschrichtius_robustus
Cephalorhynchus_commersonii
Cephalorhynchus_commersonii
Cephalorhynchus_commersonii
X
Y
X
Y
Y
X
X
Y
X
Y
Y
X

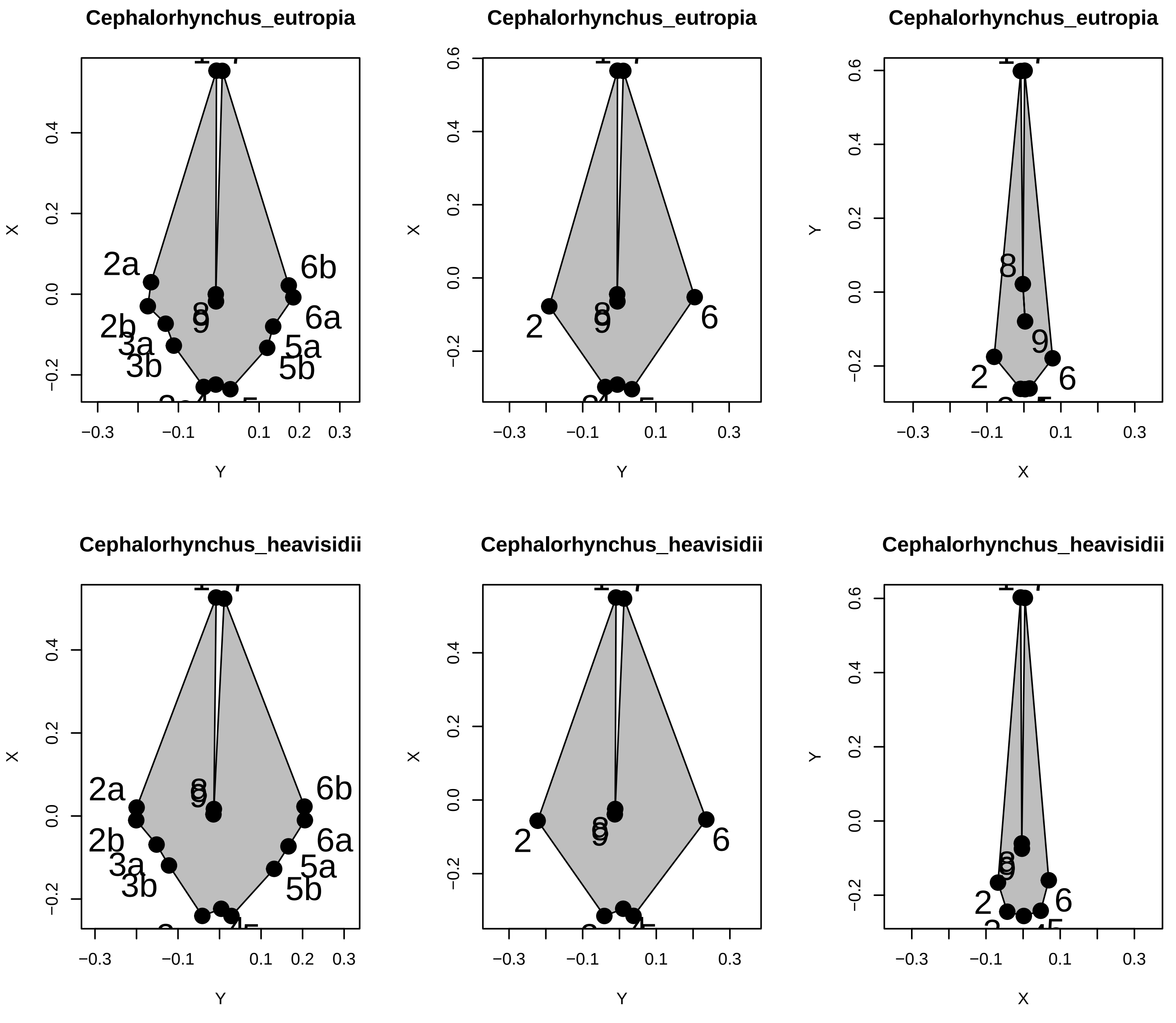

Cephalorhynchus_eutropia
Cephalorhynchus_eutropia
Cephalorhynchus_eutropia
Cephalorhynchus_heavisidii
Cephalorhynchus_heavisidii
Cephalorhynchus_heavisidii
X
Y
2a
2b
3a
3b
6b
6a
5a
5b
8
9
2
6
0.6
0.4
0.2
0.0
−0.2
−0.3
−0.1
0.1
0.2
0.3

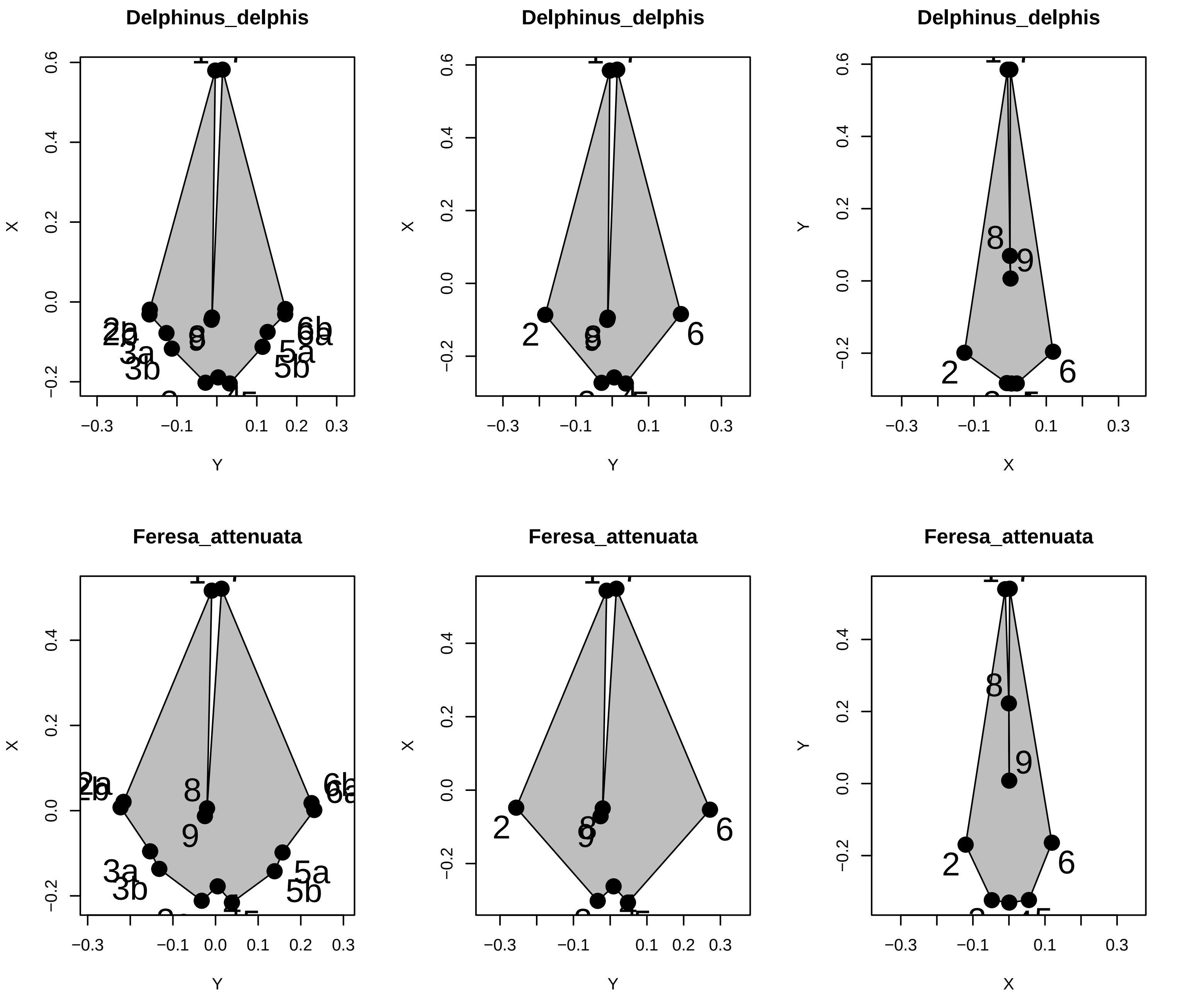
Delphinus_delphis
Delphinus_delphis
Delphinus_delphis
Feresa_attenuata
Feresa_attenuata
Feresa_attenuata
X
Y
2
6
8
9
2a
2b
3a
3b
5a
5b
6a
6b
0.6
0.4
0.2
0.0
−0.2
−0.3
−0.1
0.0
0.1
0.2
0.3

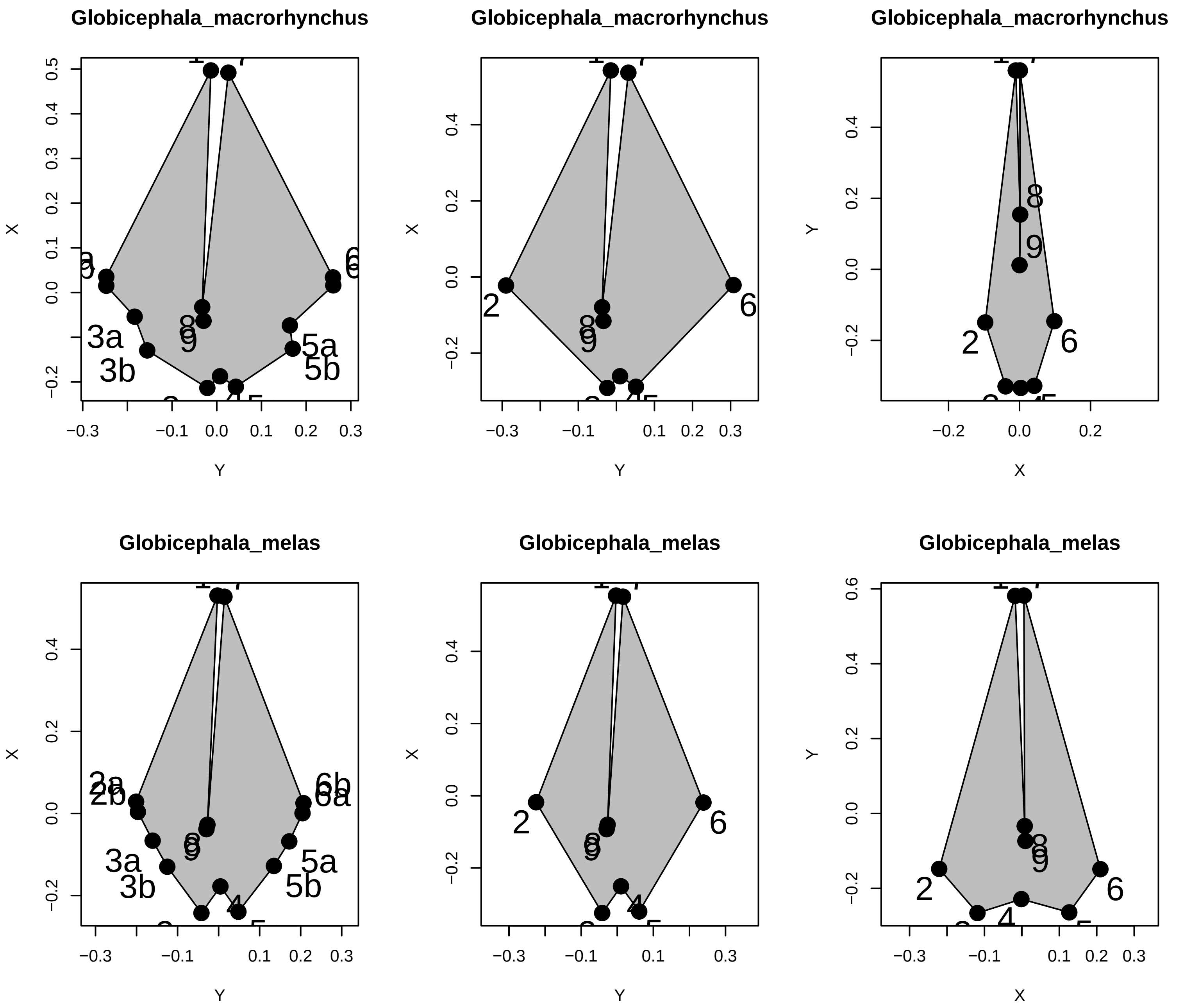
Globicephala_macrorhynchus
X
0.5
0.4
0.3
0.2
0.1
0.0
−0.2
−0.3
−0.1
0.0
0.1
0.2
0.3
Y
3a
3b
8
9
5a
5b
Globicephala_macrorhynchus
X
0.4
0.2
0.0
−0.2
−0.3
−0.1
0.1
0.2
0.3
Y
2
6
8
9
Globicephala_macrorhynchus
Y
0.4
0.2
0.0
−0.2
−0.2
0.0
0.2
X
8
9
2
6
Globicephala_melas
X
0.4
0.2
0.0
−0.2
−0.3
−0.1
0.1
0.2
0.3
Y
2a
2b
6b
6a
8
9
3a
3b
4
5a
5b
Globicephala_melas
X
0.4
0.2
0.0
−0.2
−0.3
−0.1
0.1
0.3
Y
2
6
8
9
4
Globicephala_melas
Y
0.6
0.4
0.2
0.0
−0.2
−0.3
−0.1
0.1
0.2
0.3
X
8
9
2
6
4

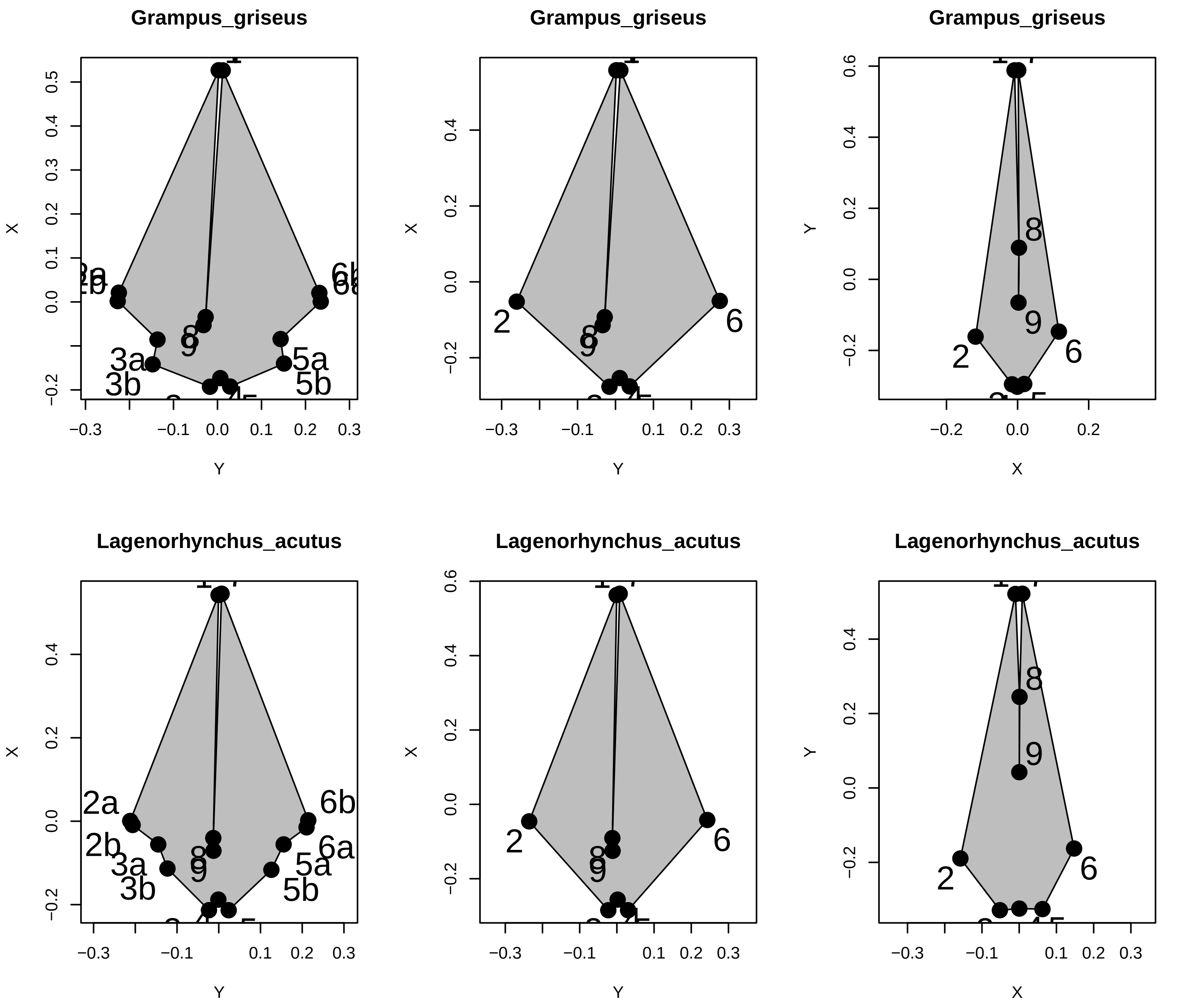
Grampus_griseus
Grampus_griseus
Grampus_griseus
Lagenorhynchus_acutus
Lagenorhynchus_acutus
Lagenorhynchus_acutus
X
Y
2a
2b
3a
3b
5a
5b
6b
6a
8
9
2
6
−0.3
−0.1
0.0
0.1
0.2
0.3
−0.2
0.4
0.5
0.6

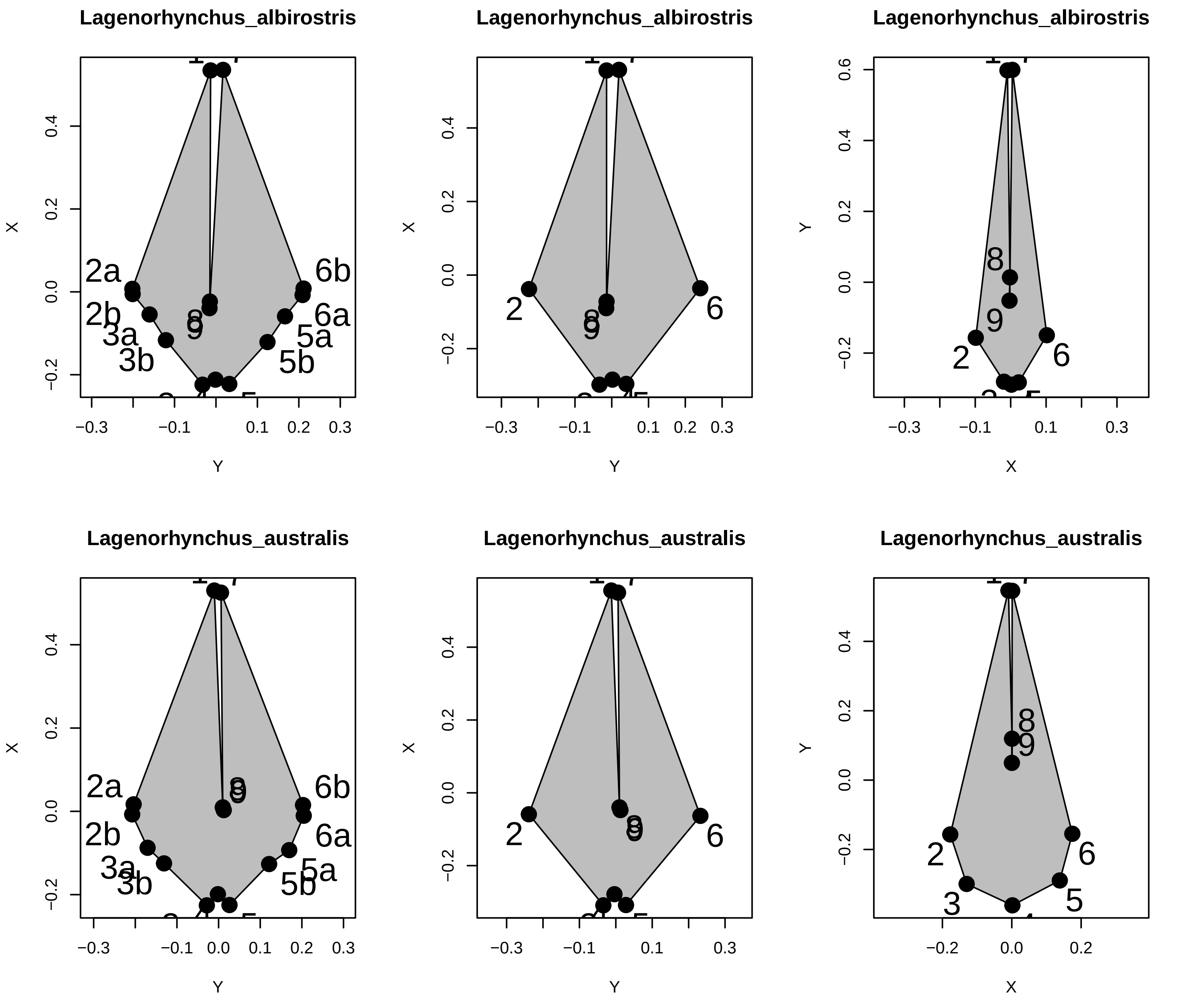
Lagenorhynchus_albirostris
Lagenorhynchus_albirostris
Lagenorhynchus_albirostris
Lagenorhynchus_australis
Lagenorhynchus_australis
Lagenorhynchus_australis
X
Y
2a
2b
3a
3b
6b
6a
5a
5b
8
9
2
6
−0.3
−0.1
0.1
0.2
0.3
−0.2
0.0
0.2
0.4
0.6
3
5

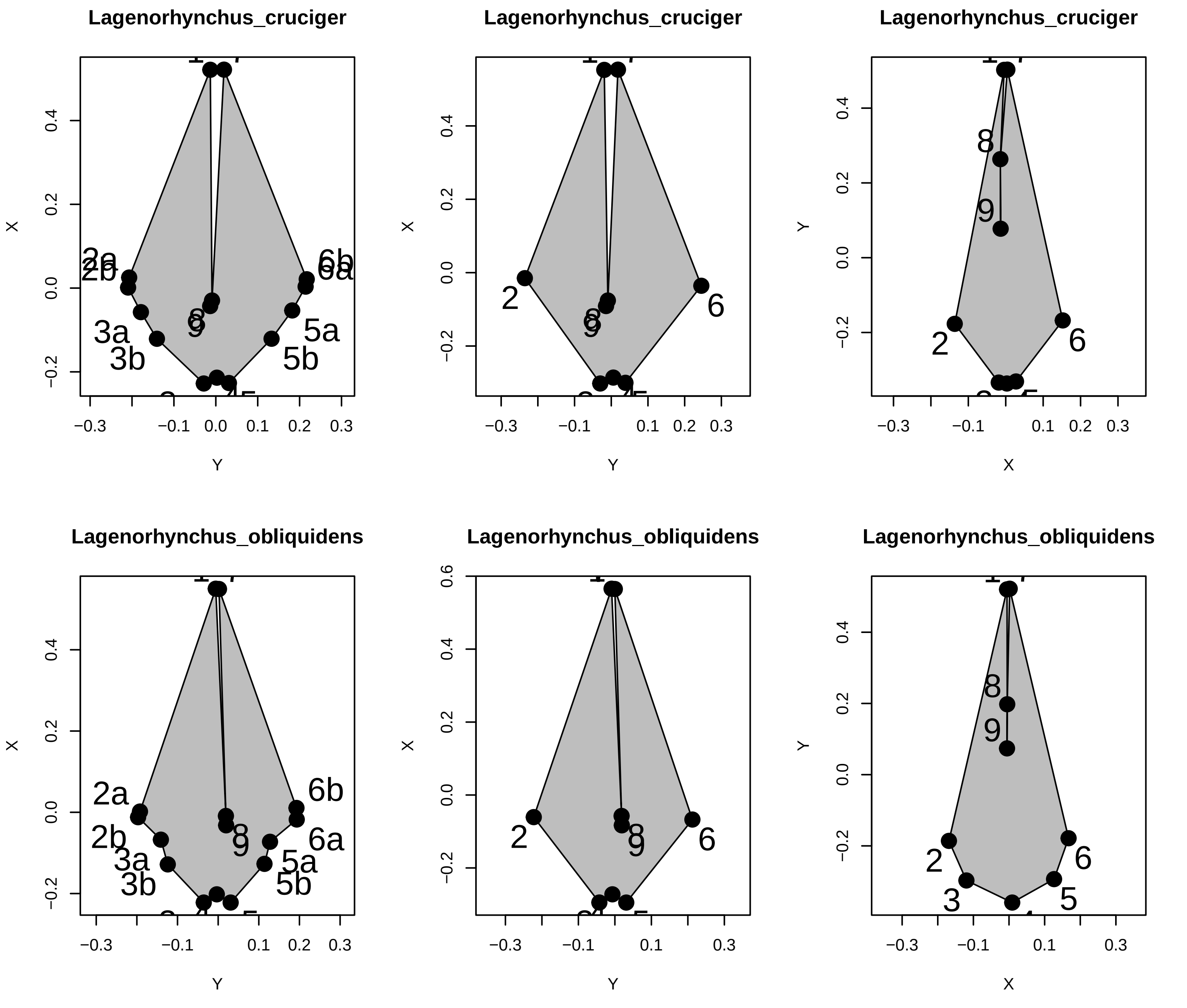
Lagenorhynchus_cruciger
Lagenorhynchus_cruciger
Lagenorhynchus_cruciger
Lagenorhynchus_obliquidens
Lagenorhynchus_obliquidens
Lagenorhynchus_obliquidens
X
Y
X
Y
Y
X
X
Y
X
Y
Y
X

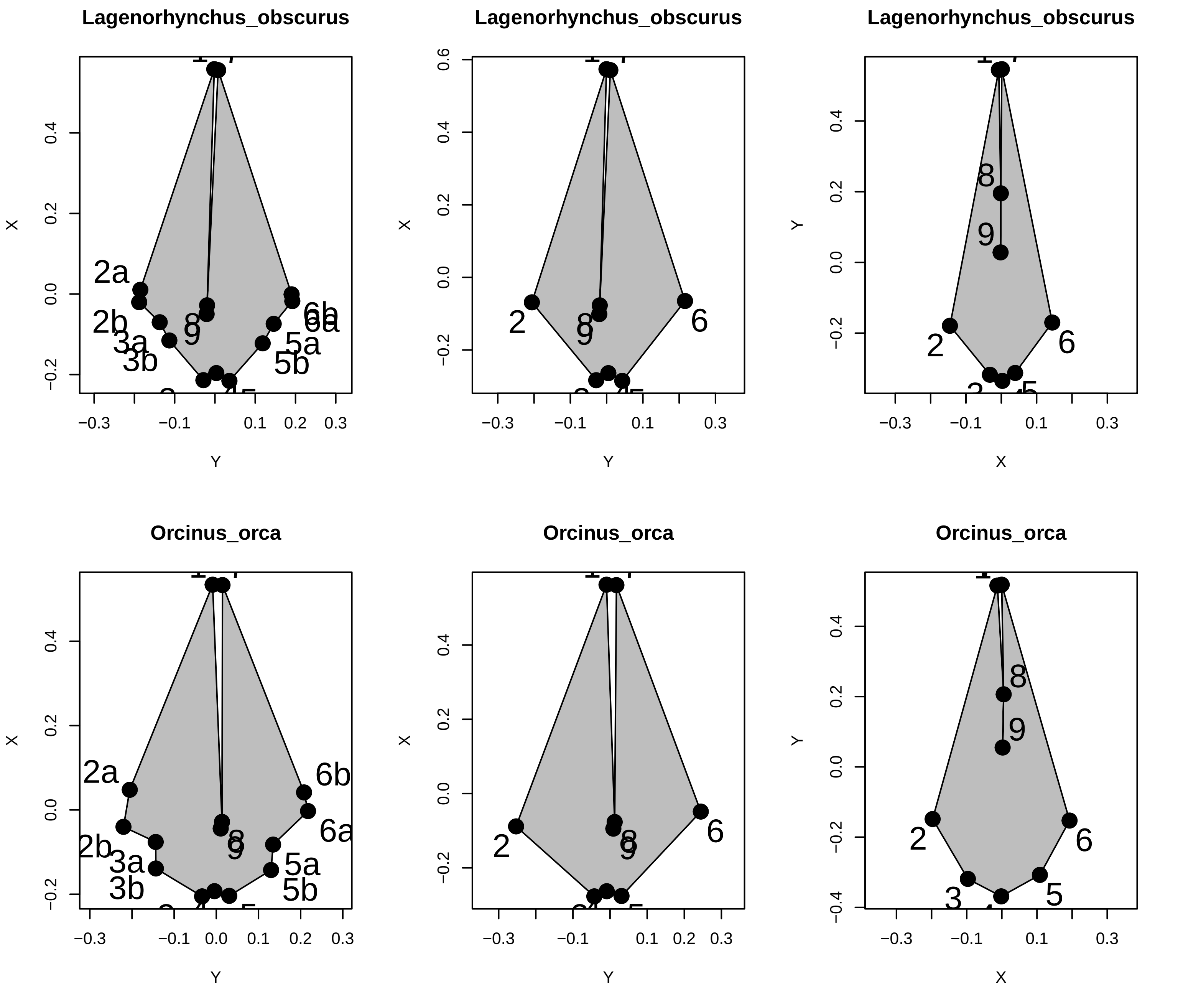

Lagenorhynchus_obscurus
Lagenorhynchus_obscurus
Lagenorhynchus_obscurus
Orcinus_orca
Orcinus_orca
Orcinus_orca
X
Y
2a
2b
3a
3b
5a
5b
6a
6b
8
9
2
6
3
5

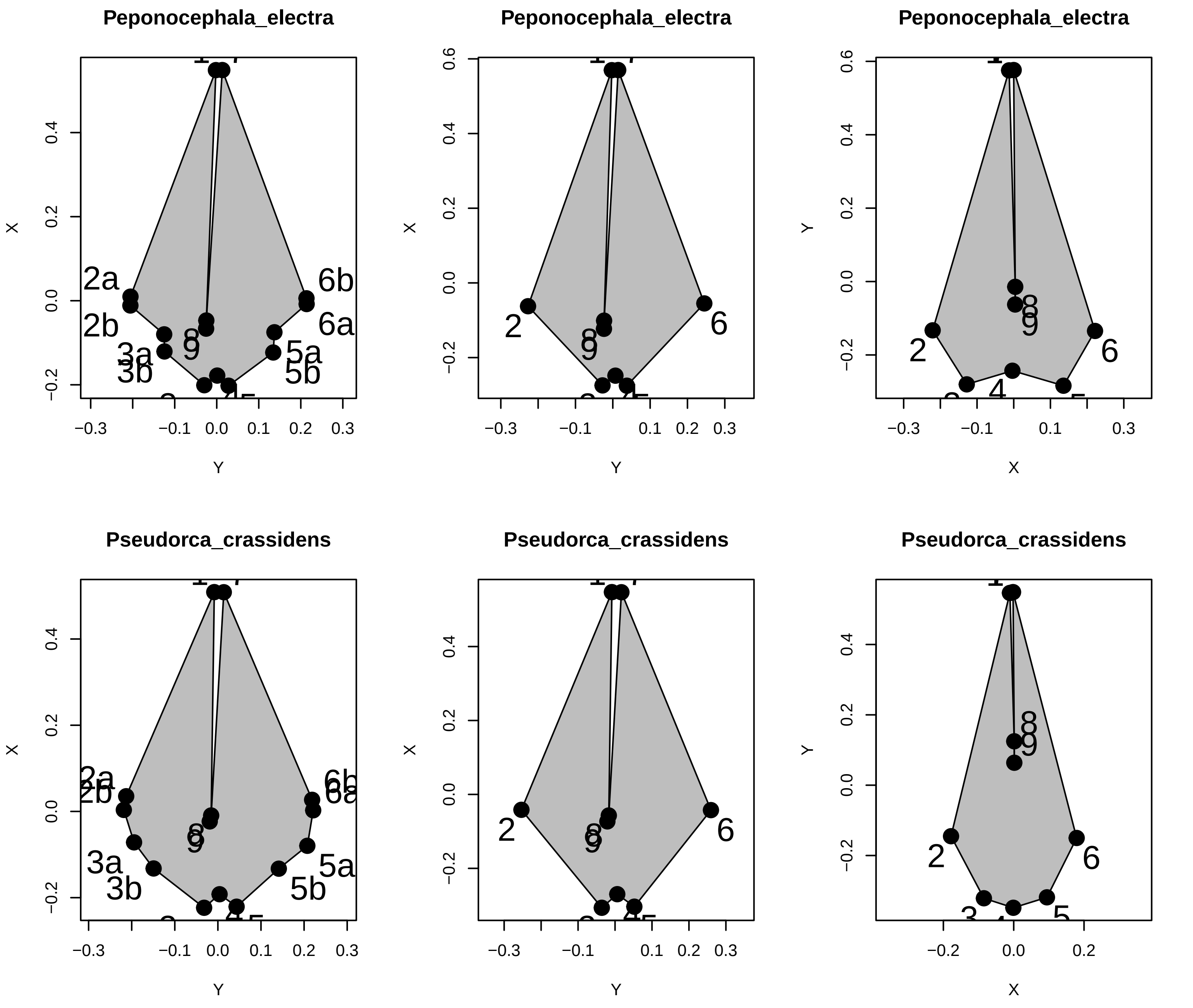
Peponocephala_electra
Peponocephala_electra
Peponocephala_electra
Pseudorca_crassidens
Pseudorca_crassidens
Pseudorca_crassidens
X
Y
Y
X
2a
2b
3a
3b
6b
6a
5a
5b
8
9
2
6
4
0.6
0.4
0.2
0.0
−0.2
−0.3
−0.1
0.0
0.1
0.2
0.3

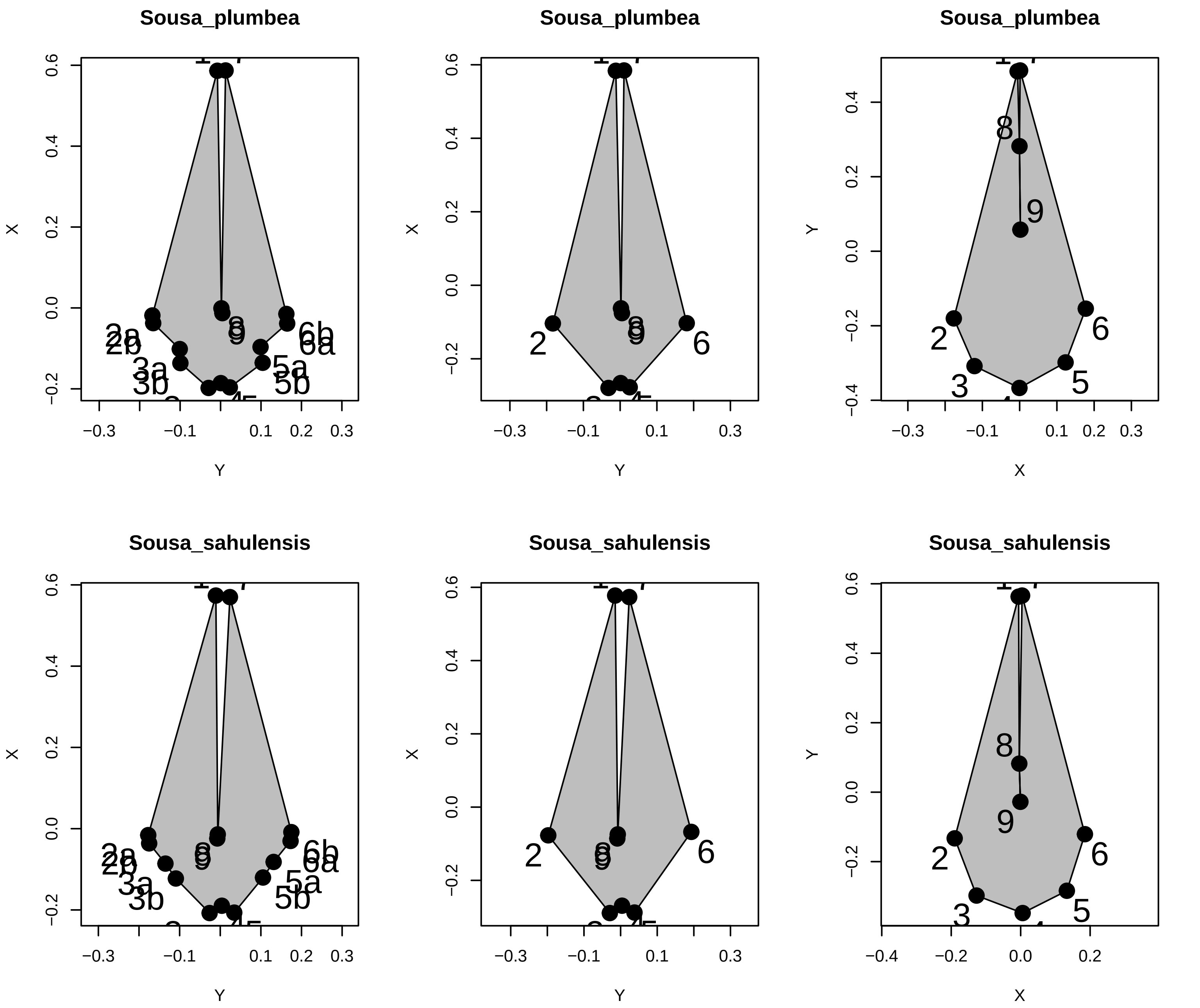
Sousa_plumbea
Sousa_plumbea
Sousa_plumbea
Sousa_sahulensis
Sousa_sahulensis
Sousa_sahulensis
X
Y
2a
2b
3a
3b
5a
5b
6a
6b
8
9
2
3
5
6
−0.3
−0.1
0.1
0.2
0.3
−0.4
−0.2
0.0
0.2
0.4
0.6

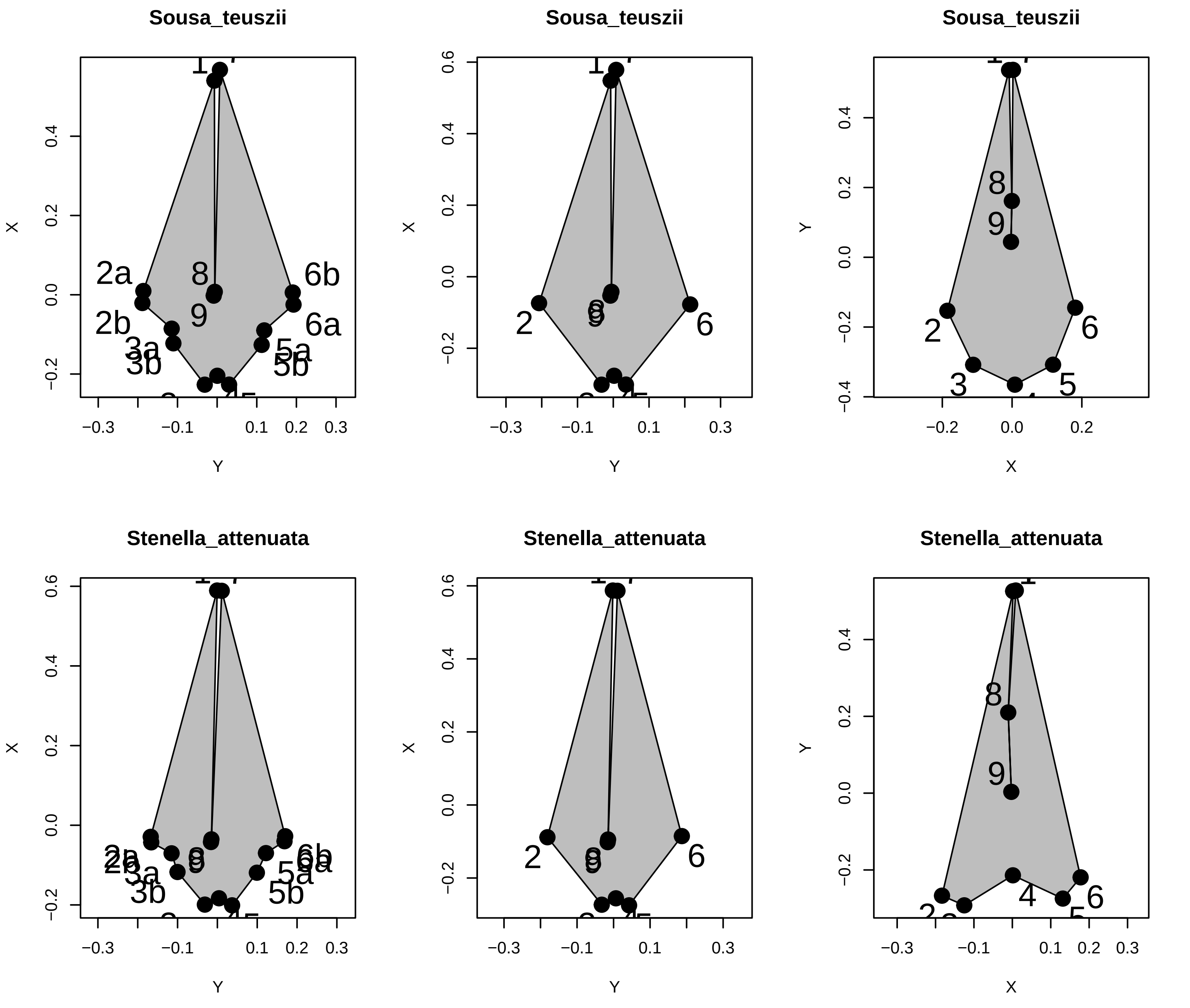
Sousa_teuszii
X
0.4
0.2
0.0
−0.2
Y
−0.3
−0.1
0.1
0.2
0.3
2a
2b
3a
3b
8
9
6b
6a
5a
5b
Sousa_teuszii
X
0.6
0.4
0.2
0.0
−0.2
Y
−0.3
−0.1
0.1
0.3
2
6
8
9
Sousa_teuszii
Y
0.4
0.2
0.0
−0.2
−0.4
X
−0.2
0.0
0.2
8
9
2
6
3
5
Stenella_attenuata
X
0.6
0.4
0.2
0.0
−0.2
Y
−0.3
−0.1
0.1
0.2
0.3
3a
3b
5a
5b
Stenella_attenuata
X
0.6
0.4
0.2
0.0
−0.2
Y
−0.3
−0.1
0.1
0.3
2
6
8
Stenella_attenuata
Y
0.4
0.2
0.0
−0.2
X
−0.3
−0.1
0.1
0.2
0.3
8
9
4
6

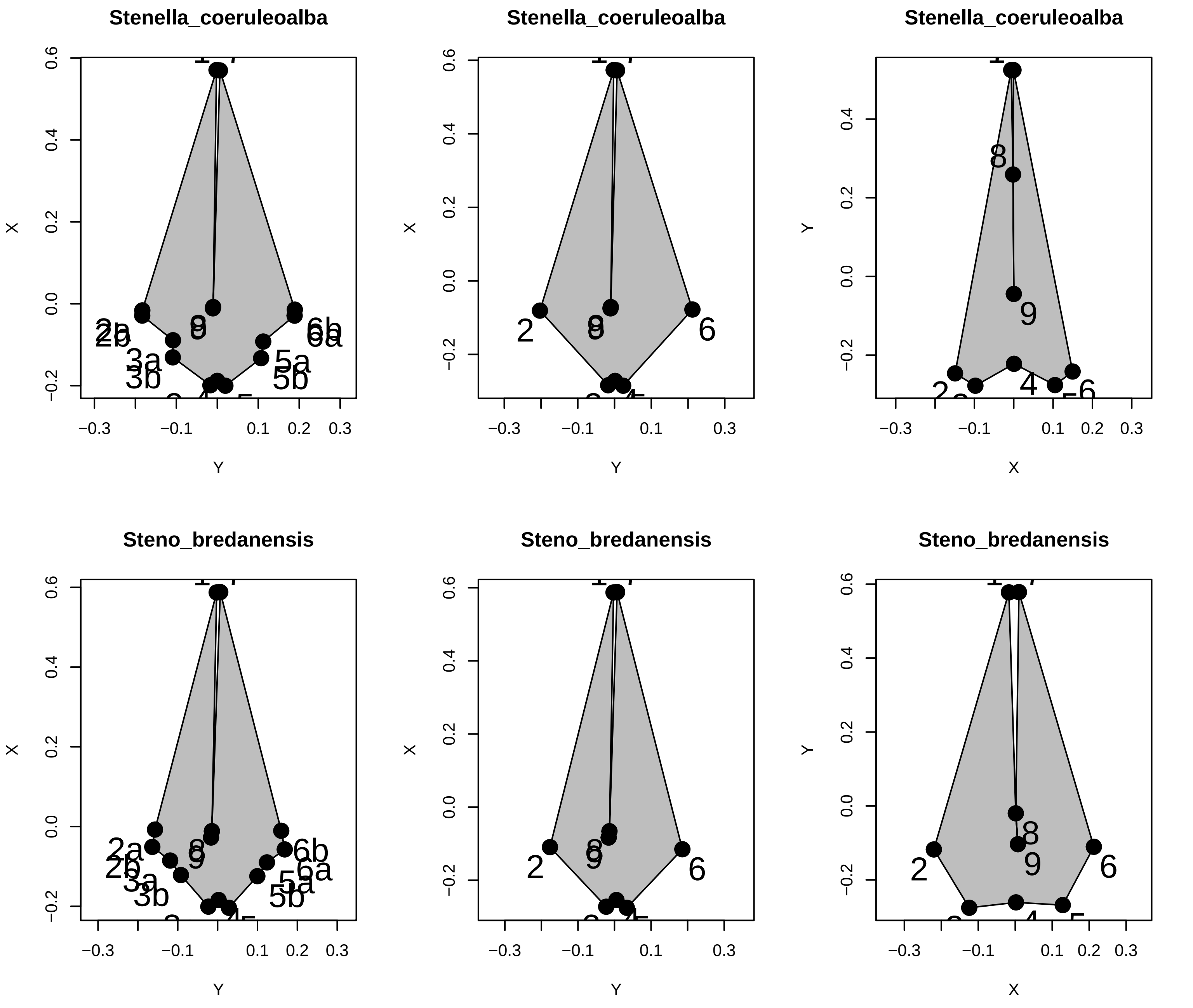

Stenella_coeruleoalba
Stenella_coeruleoalba
Stenella_coeruleoalba
Steno_bredanensis
Steno_bredanensis
Steno_bredanensis
X
Y
X
Y
Y
X
X
Y
X
Y
Y
X
2a
2b
3a
3b
8
9
6b
6a
5a
5b
2
6
4
9
6

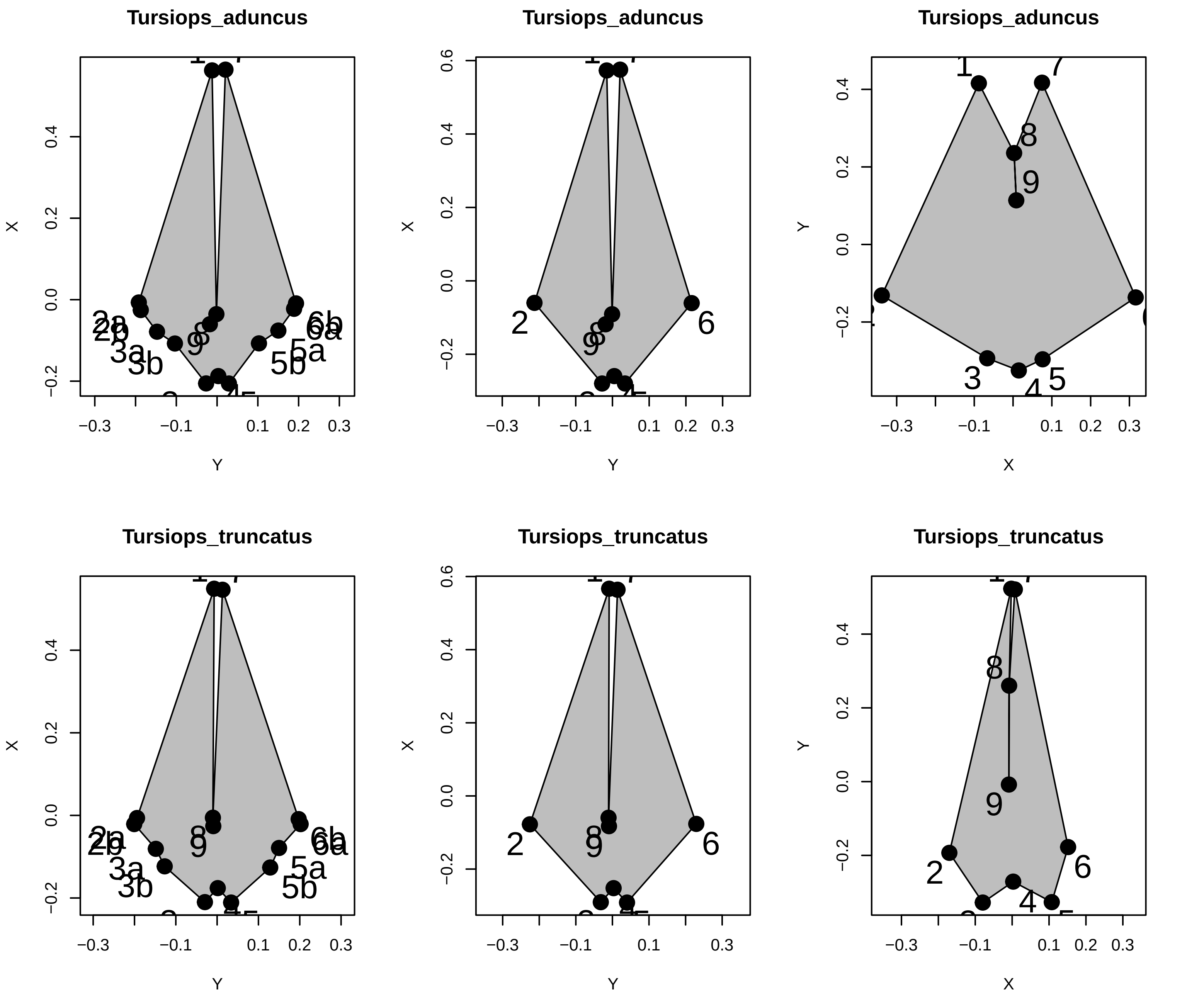
Tursiops_aduncus
Tursiops_aduncus
Tursiops_aduncus
Tursiops_truncatus
Tursiops_truncatus
Tursiops_truncatus
X
Y
2a
2b
3a
3b
5a
5b
6a
6b
1
2
3
4
5
6
7
8
9
−0.3
−0.1
0.1
0.2
0.3
−0.2
0.0
0.2
0.4
0.6

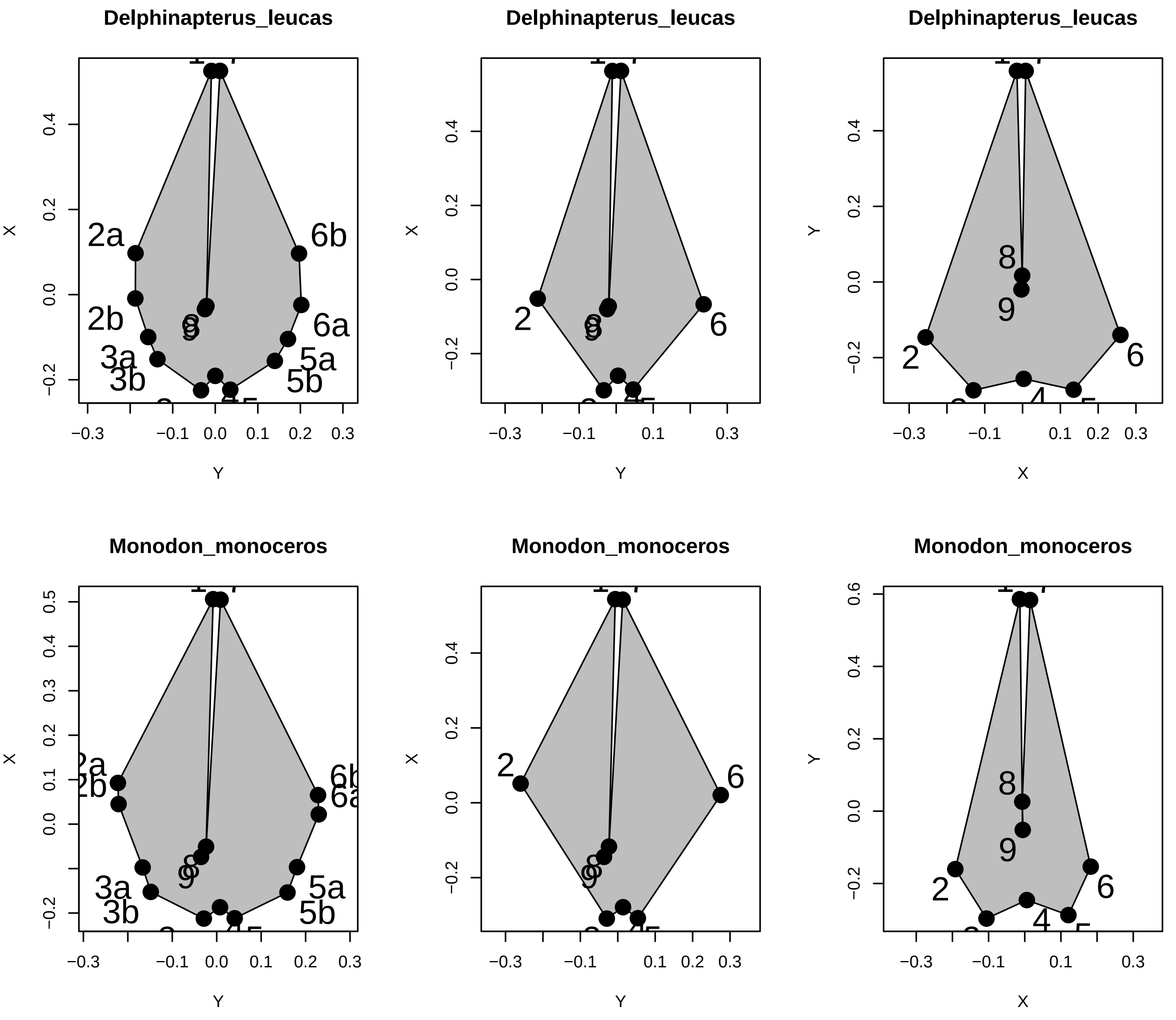

Delphinapterus_leucas
Delphinapterus_leucas
Delphinapterus_leucas
Monodon_monoceros
Monodon_monoceros
Monodon_monoceros
X
Y
2a
2b
3a
3b
6b
6a
5a
5b
2
6
8
9
−0.3
−0.1
0.0
0.1
0.2
0.3
−0.2
0.4
0.5

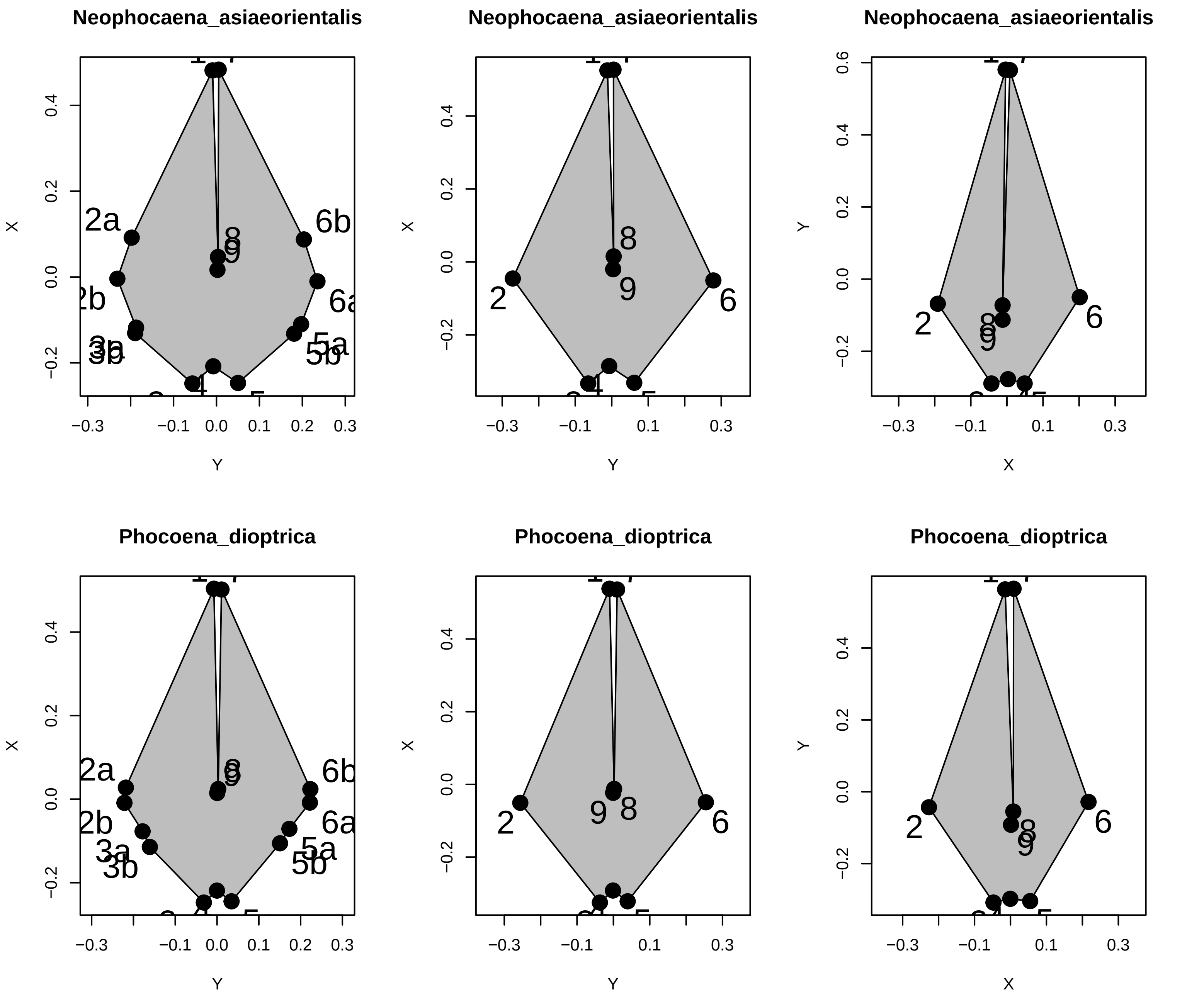

Neophocaena_asiaeorientalis
Neophocaena_asiaeorientalis
Neophocaena_asiaeorientalis
X
Y
0.4
0.2
0.0
−0.2
0.6
−0.3
−0.1
0.0
0.1
0.2
0.3
2a
2b
3a
3b
6b
6a
5a
5b
8
9
2
6
Phocoena_dioptrica
Phocoena_dioptrica
Phocoena_dioptrica

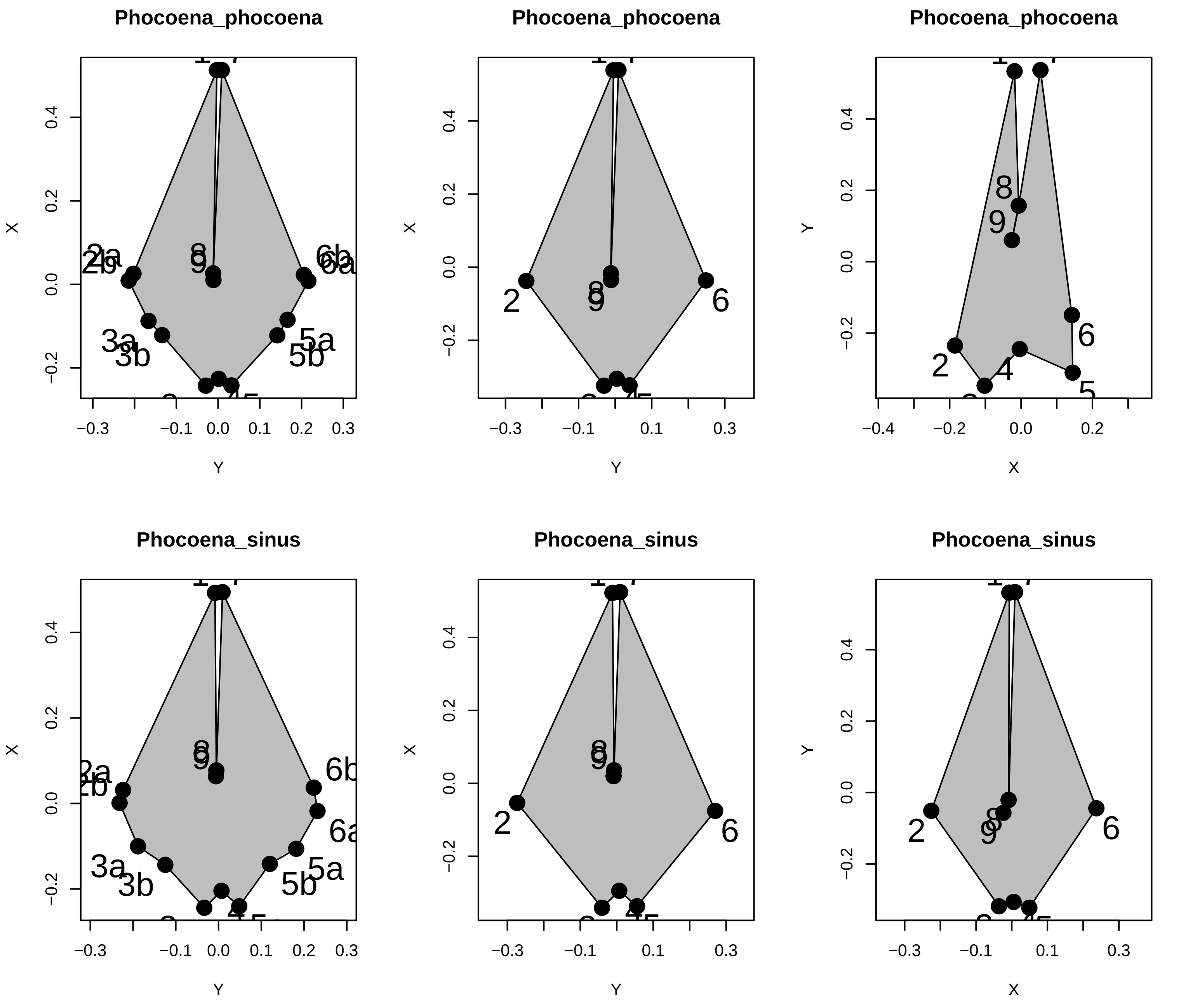

Phocoena_phocoena
Phocoena_phocoena
Phocoena_phocoena
Phocoena_sinus
Phocoena_sinus
Phocoena_sinus
X
Y
2a
2b
3a
3b
5a
5b
6a
6b
2
4
5
6
8
9

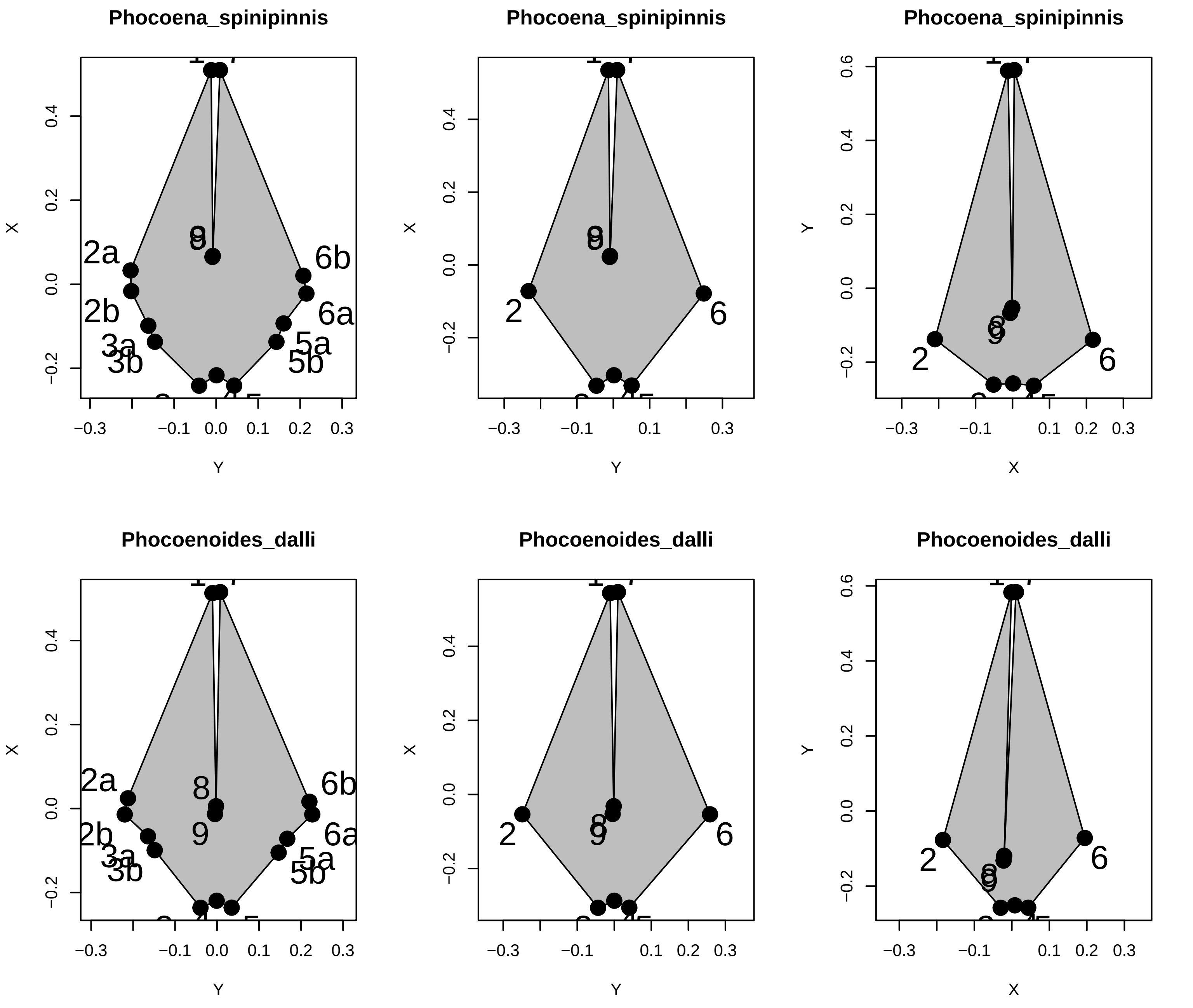
Phocoena_spinipinnis
Phocoena_spinipinnis
Phocoena_spinipinnis
Phocoenoides_dalli
Phocoenoides_dalli
Phocoenoides_dalli
X
Y
2a
2b
3a
3b
5a
5b
6a
6b
8
9
2
6
−0.3
−0.1
0.0
0.1
0.2
0.3
−0.2
0.4
0.6

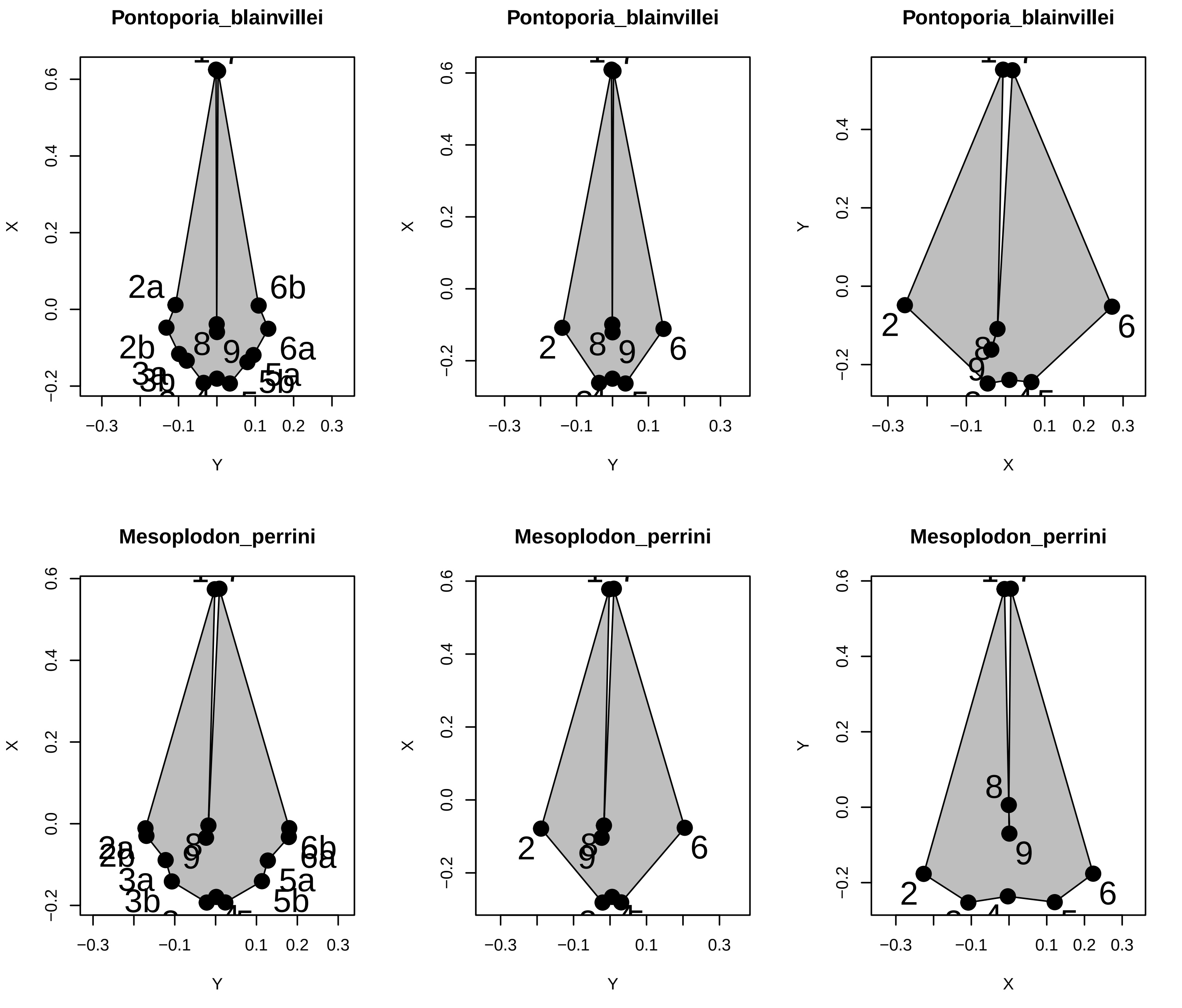

Pontoporia_blainvillei
X
Y
2a
6b
2b
6a
8
9
3a
5a
3b
5b
Pontoporia_blainvillei
X
Y
2
8
9
6
Pontoporia_blainvillei
Y
X
2
6
8
9
Mesoplodon_perrini
X
Y
2a
2b
6b
6a
8
9
3a
5a
3b
5b
Mesoplodon_perrini
X
Y
2
8
9
6
Mesoplodon_perrini
Y
X
8
9
2
6

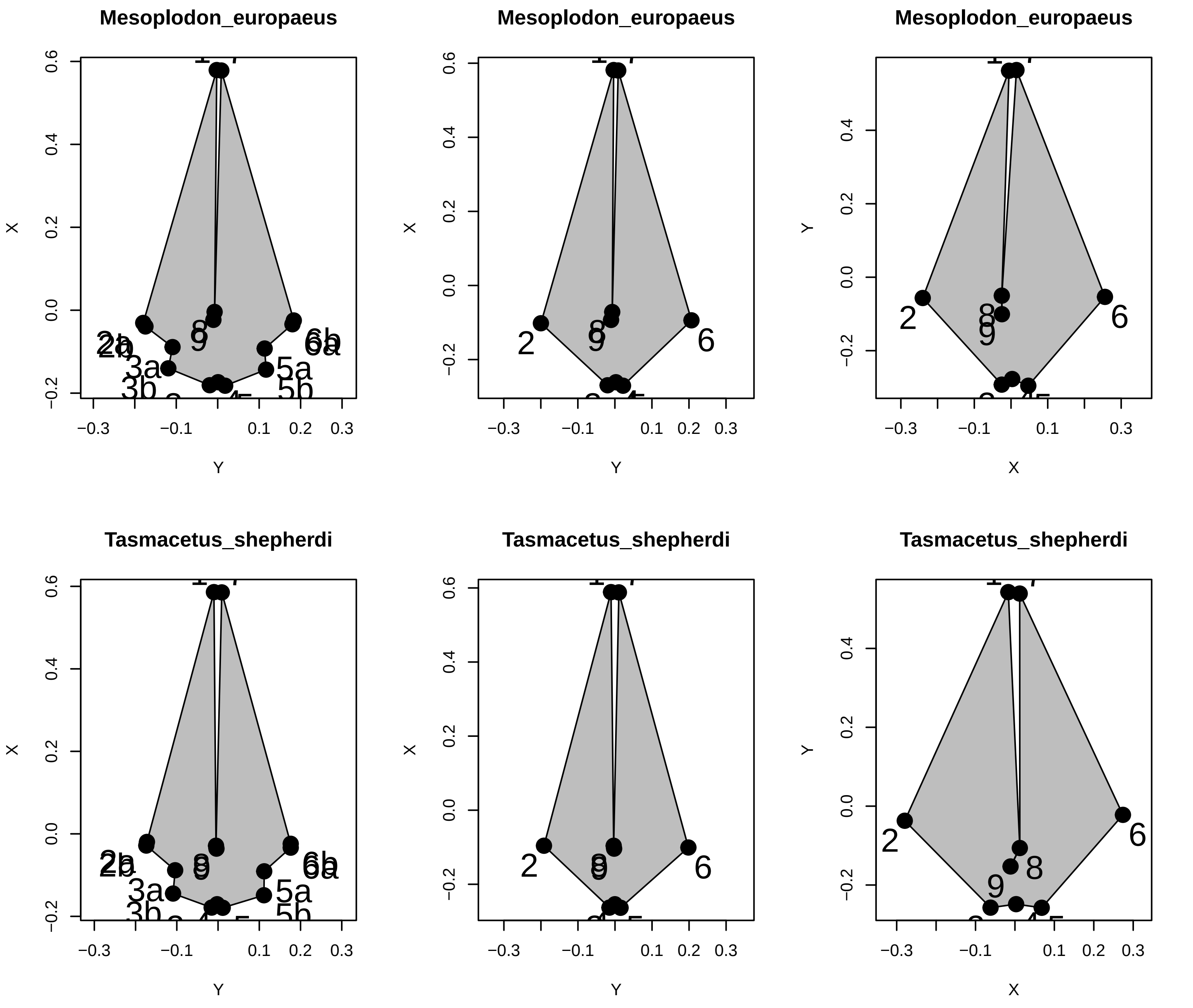

Mesoplodon_europaeus
Mesoplodon_europaeus
Mesoplodon_europaeus
Tasmacetus_shepherdi
Tasmacetus_shepherdi
Tasmacetus_shepherdi
X
Y
−0.2
0.0
0.2
0.4
0.6
−0.3
−0.1
0.1
0.2
0.3
2a
2b
3a
3b
5a
5b
6a
6b
2
6
8
9

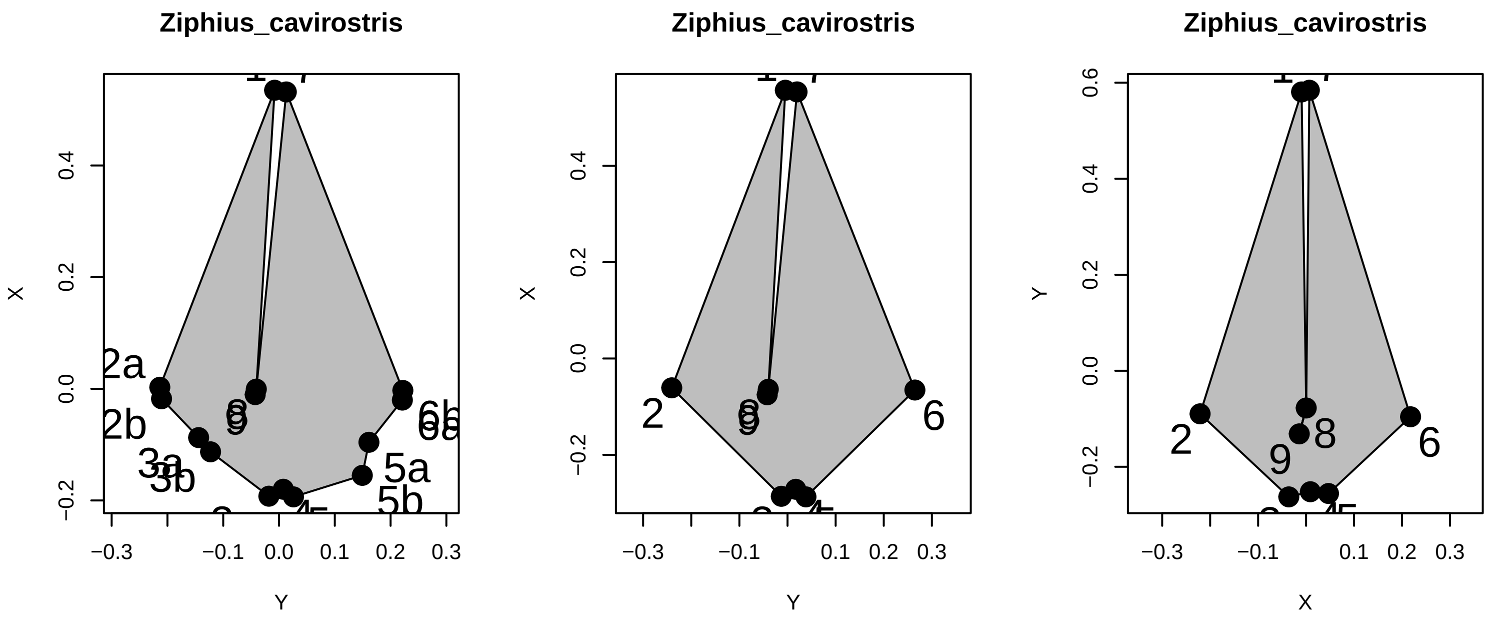
Ziphius_cavirostris
Ziphius_cavirostris
Ziphius_cavirostris
X
Y
X
Y
Y
X
0.4
0.2
0.0
−0.2
0.6
−0.3
−0.1
0.1
0.2
0.3
2a
2b
3a
3b
5a
5b
2
6
8
9
